# Supplementary material for: Healthcare resource utilisation and costs associated with a heart failure diagnosis: a retrospective, population-based cohort study in Sweden
Source: BMJ Open. 2021 Oct 18;11(10):e053806. doi: 10.1136/bmjopen-2021-053806 (PMC8527145; doi:10.1136/bmjopen-2021-053806)
Supplement: Supplementary data [file bmjopen-2021-053806supp001.pdf]

## Supporting information

### Secondary healthcare costs: pharmacotherapy (the Uppsala County cohort)

In the cohort of patients from the Uppsala County with incident patients with heart failure (HF) during the period of 2010–2012 (N=3,452), the overall drug use (HF-related drugs and other pharmacotherapies) in the year after diagnosis was associated with a mean cost of Swedish krona (SEK) 10,727 (equivalent to €1,126) per patient per year. In general, these costs remained stable for up to 4 years after the diagnosis of HF. However, at year 5, an increase was observed in the mean cost to SEK 12,760 (€1,340) per patient per year. The costs of HF-specific therapy (drugs listed in Table S2) constituted 13% of the total drug costs in the first year after HF diagnosis (mean costs per patient per year at years 1 and 5 after HF diagnosis were SEK 1,395 [€146] and 1,404 [€147], respectively).

**Table S1** List of ICD-10 codes

| **Comorbidity/risk factor** | **ICD-10 code** |
|---|---|
| Hypertension | I10–I13, I15 |
| Atrial fibrillation | I48 |
| Angina or MI | I20–I22 |
| Diabetes | E10–E14 |
| Cancer | C00–C97 |
| Cerebrovascular disease | I60–I69 |
| Dyslipidaemia | E78 |
| Anaemia | D50–D64 |
| COPD | J40–J44, J47 |
| Aortic insufficiency/regurgitation | I06, I35, Q231 |
| Dementia | F00–F07 |
| Chronic kidney disease | N18 |
| Depression | F32–F33 |
| Mitral insufficiency/regurgitation | I051, I340, Q233 |
| Peripheral artery disease | I739 |
| **Definitions for calculation of CCI** | |
| MI | I21–I22, I252 |
| Congestive heart failure | I43, I50, I099, I110, I130, I132, I255, I420, I425–I429, P290 |
| Peripheral vascular disease | I70–I71, I731, I738–I739, I771, I790, I792, K551, K558–K559, Z958–Z959 |

| | |
|---|---|
| Cerebrovascular disease | G45–G46, I60–I69, H340 |
| Dementia | F00–F03, G30, F051, G311 |
| Chronic pulmonary disease | J40–J47, J60–J67, I278–I279, J684, J701, J703 |
| Connective tissue disease-rheumatic disease | M05–M06, M315, M32–M34, M351, M353, M360 |
| Peptic ulcer disease | K25–K28 |
| Mild liver disease | B18, K73–K74, K700–K703, K709, K713–K715, K717, K760, K762–K764, K768–K769, Z944 |
| Diabetes without complications | E100–E101, E106, E108–E111, E116, E118–E121, E126, E128–E131, E136, E138–E141, E146, E148–E149 |
| Diabetes with complications | E102–E105, E107, E112–E115, E117, E122–E125, E127, E132–E135, E137, E142–E145, E147 |
| Paraplegia and hemiplegia | G81–G82, G041, G114, G801–G802, G830–G834, G839 |
| Renal disease | N18–N19, N052–N057, N250, I120, I131, N032–N037, Z490–Z492, Z940, Z992 |
| Cancer | C00–C76, C81–C97 |
| Moderate to severe liver disease | K704, K711, K721, K729, K765–K767, I850, I859, I864, I982 |
| Metastatic carcinoma | C77–C80 |
| AIDS/HIV | B20–B22, B24 |

AIDS/HIV, human immunodeficiency virus/acquired immunodeficiency syndrome; CCI, Charlson comorbidity index; COPD, chronic obstructive pulmonary disease; ICD-10, International Classification of Diseases and Related Health Problems, Tenth Revision; MI, myocardial infarction.

**Table S2** Heart failure-related drugs (according to the European Society of Cardiology 2012 guidelines[9])

| Drug/drug class | Anatomical Therapeutic Chemical code |
|---|---|
| Angiotensin-converting enzyme inhibitor | C09A, C09B |
| Angiotensin receptor blocker | C09C, C09D |
| Beta blocker | C07 |
| Mineralocorticoid receptor antagonist | C03DA01-C03DA04 |
| Ivabradine (sinus node $I_f$ channel inhibitor) | C01EB17 |
| Diuretics | C03 |
| Digoxin | C01AA05 |
| Nitrates and nitrites | C01DA |

**Table S3** Sub-distribution hazard model of the proportion of incident patients with HF from the Uppsala and Västerbotten counties with a second HF diagnosis in 2010–2012 who had all-cause hospitalizations within 3 years and cumulative incidence function estimates for the proportion of hospitalized patients within 0.5, 1, and 3 years after index date

| **Characteristic** | **N=4,648** | **Cumulative incidence function of all-cause hospitalizations, % (95% CI)** | | | **Sub-distribution HR (95% CI)** | ***P* value** |
|---|---|---|---|---|---|---|
| | | **0.5 years** | **1 year** | **3 years** | | |
| Age group, years | | | | | | |
| 18–54 (reference) | 275 | 51.6 (45.6–57.4) | 58.2 (52.1–63.8) | 69.5 (63.0–75.0) | 1 | |
| 55–64 | 457 | 50.3 (45.6–54.8) | 60.4 (55.7–64.7) | 75.2 (70.1–79.5) | 1.07 (0.88–1.29) | 0.50 |
| 65–74 | 1,051 | 50.5 (47.5–53.5) | 60.7 (57.7–63.6) | 75.8 (72.7–78.6) | 1.11 (0.93–1.31) | 0.24 |
| 75–84 | 1,592 | 46.2 (43.8–48.7) | 57.4 (54.9–59.8) | 77.3 (74.7–79.8) | 1.06 (0.90–1.25) | 0.51 |
| ≥85 | 1,273 | 43.4 (40.6–46.1) | 53.7 (51.0–56.4) | 72.4 (69.5–75.0) | 0.97 (0.82–1.16) | 0.77 |
| Sex | | | | | | |
| Female (reference) | 2,143 | 46.3 (44.2–48.4) | 55.6 (53.5–57.7) | 74.5 (72.2–76.6) | 1 | |
| Male | 2,505 | 47.8 (45.9–49.8) | 59.1 (57.1–61.0) | 75.5 (73.4–77.4) | 1.04 (0.97–1.12) | 0.28 |

| | | | | | | |
|---|---|---|---|---|---|---|
| Diagnosis setting | | | | | | |
| Primary care (reference) | 1,118 | 43.7 (40.8–46.6) | 56.1 (53.1–58.9) | 79.9 (76.7–82.8) | 1 | |
| Secondary care | 3,528 | 48.2 (46.5–49.8) | 57.9 (56.3–59.5) | 73.6 (71.8–75.2) | 0.93 (0.86–1.01) | 0.07 |
| Unknown | 2 | N/A | N/A | N/A | 1.80 (1.15–2.82) | 0.0098 |
| HF phenotype | | | | | | |
| HFrEF (reference) | 609 | 56.5 (52.4–60.3) | 64.7 (60.8–68.4) | 80.5 (76.5–83.9) | 1 | |
| HFpEF | 1,106 | 54.9 (51.9–57.8) | 63.7 (60.8–66.5) | 76.4 (73.4–79.0) | 0.91 (0.81–1.03) | 0.14 |
| Unknown LVEF | 2,933 | 42.3 (40.5–44.1) | 53.6 (51.8–55.4) | 73.6 (71.6–75.4) | 0.77 (0.70–0.86) | <0.0001 |
| NT-proBNP, pg/mL | | | | | | |
| 0–1,000 (reference) | 912 | 43.0 (39.7–46.2) | 55.2 (51.9–58.3) | 76.4 (73.0–79.6) | 1 | |
| 1,001–3,000 | 1,283 | 47.3 (44.6–50.0) | 58.6 (55.9–61.3) | 78.0 (75.2–80.6) | 1.06 (0.97–1.17) | 0.21 |
| >3,000 | 1,601 | 54.5 (52.0–56.9) | 63.7 (61.3–66.0) | 76.4 (73.9–78.7) | 1.19 (1.07–1.31) | 0.0007 |
| Missing | 852 | 37.6 (34.3–40.8) | 46.6 (43.2–49.9) | 66.2 (62.5–69.7) | 0.84 (0.75–0.94) | 0.0028 |

CI, confidence interval; HF, heart failure; HFpEF, HF with preserved ejection fraction; HFrEF, HF with reduced ejection fraction; HR, hazard ratio; LVEF, left ventricular ejection fraction; NT-proBNP, N-terminal pro-B-type natriuretic peptide.

**Note:** Multivariate sub-distribution hazard model with death as competing risk used to obtain the sub-distribution HRs. Cumulative incidence function was used to estimate percentage of patients with all-cause hospitalizations in 0.5, 1, and 3 years after index date.

**Table S4** Costs associated with primary care contacts for incident patients with HF between 2010 and 2012 in the Uppsala County

| | Mean (SD) costs per patient per year in SEK | | | | |
|---|---|---|---|---|---|
| | **Year 1 (N=3,453)** | **Year 2 (N=2,705)** | **Year 3 (N=1,846)** | **Year 4 (N=891)** | **Year 5 (N=166)** |
| Total costs | 10,347 (12,685) | 9,521 (11,974) | 9,490 (12,225) | 9,754 (12,755) | 10,869 (16,857) |
| Family physician visits | 5,432 (8,634) | 4,718 (7,723) | 4,668 (7,652) | 4,641 (7,693) | 5,153 (7,714) |
| Nurse visits | 4,050 (7,251) | 4,068 (7,475) | 4,100 (7,546) | 4,388 (8,107) | 4,992 (11,685) |
| Blood tests | 866 (1,254) | 736 (1,143) | 722 (1,133) | 726 (1,128) | 724 (1,034) |

HF, heart failure; SD, standard deviation; SEK, Swedish krona.

**Notes:** Primary care costs (available only for the Uppsala County cohort) per visit or per test were calculated based on published price lists. All costs are in SEK 2015 values (1 SEK = €0.105).



Boman K, *et al. BMJ Open* 2022; 11:e053806. doi: 10.1136/bmjopen-2021-053806
