# Supplementary material for: Healthcare resource utilisation and costs associated with a heart failure diagnosis: a retrospective, population-based cohort study in Sweden
Source: BMJ Open. 2021 Oct 18;11(10):e053806. doi: 10.1136/bmjopen-2021-053806 (PMC8527145; doi:10.1136/bmjopen-2021-053806)
Supplement: Author's manuscript [file bmjopen-2021-053806.draft_revisions.pdf]

BMJ Open is committed to open peer review. As part of this commitment we make the peer review history of every article we publish publicly available.

When an article is published we post the peer reviewers' comments and the authors' responses online. We also post the versions of the paper that were used during peer review. These are the versions that the peer review comments apply to.

The versions of the paper that follow are the versions that were submitted during the peer review process. They are not the versions of record or the final published versions. They should not be cited or distributed as the published version of this manuscript.

BMJ Open is an open access journal and the full, final, typeset and author-corrected version of record of the manuscript is available on our site with no access controls, subscription charges or pay-per-view fees (http://bmjopen.bmj.com).

If you have any questions on BMJ Open's open peer review process please email info.bmjopen@bmj.com

BMJ Open

Healthcare resource utilization and costs associated with a heart failure diagnosis: a retrospective, population-based cohort study in Sweden.

Journal:	*BMJ Open*
Manuscript ID	bmjopen-2021-053806
Article Type:	Original research
Date Submitted by the Author:	24-May-2021
Complete List of Authors:	Boman, Kurt; Umea University Department of Public Health and Clinical Medicine, Medicine<br>Lindmark, Krister; Umea University, Dept of public health and clinical medicine and Heart Centre<br>Stålhammar, Jan; Uppsala University, Dept of Public Health and Caring Sciences<br>Olofsson, Mona<br>Costa-Scharplatz, Madlaina<br>Fonseca, Ana Filipa; Novartis Pharma AG<br>Johansson, Stina; IQVIA<br>Heller, Vincent; IQVIA<br>Törnblom, Michael<br>Wikström, Gerhard; Uppsala University Hospital, Institute of Medical Sciences
Keywords:	Heart failure < CARDIOLOGY, Adult cardiology < CARDIOLOGY, HEALTH ECONOMICS

SCHOLARONE™
Manuscripts

I, the Submitting Author has the right to grant and does grant on behalf of all authors of the Work (as defined in the below author licence), an exclusive licence and/or a non-exclusive licence for contributions from authors who are: i) UK Crown employees; ii) where BMJ has agreed a CC-BY licence shall apply, and/or iii) in accordance with the terms applicable for US Federal Government officers or employees acting as part of their official duties; on a worldwide, perpetual, irrevocable, royalty-free basis to BMJ Publishing Group Ltd ("BMJ") its licensees and where the relevant Journal is co-owned by BMJ to the co-owners of the Journal, to publish the Work in this journal and any other BMJ products and to exploit all rights, as set out in our licence.

The Submitting Author accepts and understands that any supply made under these terms is made by BMJ to the Submitting Author unless you are acting as an employee on behalf of your employer or a postgraduate student of an affiliated institution which is paying any applicable article publishing charge ("APC") for Open Access articles. Where the Submitting Author wishes to make the Work available on an Open Access basis (and intends to pay the relevant APC), the terms of reuse of such Open Access shall be governed by a Creative Commons licence – details of these licences and which Creative Commons licence will apply to this Work are set out in our licence referred to above.

Other than as permitted in any relevant BMJ Author's Self Archiving Policies, I confirm this Work has not been accepted for publication elsewhere, is not being considered for publication elsewhere and does not duplicate material already published. I confirm all authors consent to publication of this Work and authorise the granting of this licence.

For peer review only

Healthcare resource utilization and costs associated with a heart failure diagnosis: a retrospective, population-based cohort study in Sweden.

Kurt Boman,[1] Krister Lindmark,[2] Jan Stålhammar,[3] Mona Olofsson,[1] Madlaina Costa-Scharplatz,[4] Ana Filipa Fonseca,[5] Stina Johansson,[6] Vincent Heller,[6] Michael Törnblom,[6] Gerhard Wikström[7]

[1]Research Unit, Medicine-Geriatric, Skellefteå County Hospital, Department of Public Health and Clinical Medicine, Umeå University, Umeå, Sweden; [2]Department of Public Health and Clinical Medicine and Heart Centre, Umeå University Hospital, Umeå, Sweden; [3]Department of Public Health and Caring Sciences, Family Medicine and Preventive Medicine, Uppsala University, Uppsala, Sweden; [4]Novartis Sweden AB, Stockholm, Sweden; [5]Novartis Pharma AG, Basel, Switzerland; [6]IQVIA, Solna, Sweden; [7]Institute for Medical Sciences, Uppsala University, Uppsala, Sweden

Corresponding author

Kurt Boman

Department of Public health and Clinical Medicine,

Umeå University, SE-901 87

Umea, Sweden

Sweden

Tel: +46910771113

Fax: +4691010274

Email: Kurt.Boman@regionvasterbotten.se

Abstract

For peer review only

Objectives: To examine healthcare resource use (HRU) and costs among heart failure (HF) patients using population data from Sweden.

Design: Retrospective, non-interventional cohort study

Setting: Two cohorts were identified from linked national health registers (cohort 1, 2005–2014) and electronic medical records (cohort 2, 2010–2012; primary/secondary care patients from Uppsala and Västerbotten).

Participants Patients (aged ≥18 years) with primary or secondary diagnoses of HF (≥2 ICD-10 classification) during the identification period of January 2005 to March 2015 were included.

Outcome measures: HRU across the HF phenotypes was assessed with logistic regression. Costs were estimated based on diagnosis-related group codes and general price lists.

Results: Total annual costs of secondary care of prevalent HF increased from SEK 6.23 (€0.60) to 8.86 (€0.85) billion between 2005 and 2014. Of 4,648 incident patients, HF phenotype was known for 1,715: reduced ejection fraction [HFrEF]: 64.5%, preserved ejection fraction [HFpEF]: 35.5%. Within 1 year of HF diagnosis, the proportion of patients hospitalized was only marginally higher for HFrEF vs HFpEF (all-cause: 64.7% vs 63.7%, hazard ratio [HR] 0.91, P=0.1404; cardiovascular disease related: 61.1% vs 60.9%, HR 0.93, P=0.2769). Frequency of hospitalizations and outpatient visits per patient declined after the first year. All-cause secondary care costs in the first year were SEK 122,758 (€12,890)/patient/year, with HF-specific care accounting for 69% of the costs. Overall, 10% of the most expensive population (younger; predominantly male; more likely to have comorbidities) incurred ~40% of total secondary care costs.

For peer review only

Conclusions: HF-associated costs and HRU are high, especially during the first year of diagnosis. This is driven by high hospitalizations rates. Understanding the profile of resource-intensive patients being at younger age, male sex, and high CCI scores and NT-proBNP levels at the time of the HF diagnosis is most likely a sign of more severe disease and thus more interventions taking place.

Key words: Heart failure, resource use, cost, HF phenotypes, real-world

Strengths and limitations of this study

- The study provides comprehensive information from two county regions in Sweden on the increase in number of newly diagnosed HF patients, resource use across the HF phenotypes and total annual costs of secondary care of prevalent HF in Sweden.
- The study provides new insights about the profile of resource-intensive patients at the time of the HF diagnosis, which can support guidance in resource allocation and improve patient management.
- A retrospective identification of patients with confirmed HF despite the requirement for at least two HF diagnoses is challenging.
- Primary and secondary care resource use and costs were available only for the Uppsala Region cohort, which limited the direct comparison with the data from the Västerbotten Region where only secondary care resources were available.
- Our data reflect the real-world costs associated with a diagnosis of HF, despite an acknowledged limitation in the true validation of the diagnosis due to lack of echocardiogram records for the majority of patients.

Introduction

For peer review only

Heart failure (HF) represents a major public health challenge in developed countries and is associated with high healthcare resource consumption[1] with cardiovascular diseases (CVD) as one of the leading cause of mortality in Sweden.[2] HF places a considerable burden on the healthcare systems,[3, 4] and approximately 20% of patients discharged from hospitals after an HF admission were readmitted within 30 days between 2010 and 2013.[5] Inpatient HF care accounts for the largest proportion of healthcare costs,[6] and that the total annual direct cost related to HF is Swedish krona 2.6 billion, corresponding to approximately 2% of the total Swedish healthcare budget.[7]

An ageing population, increasing number of comorbidities, and changed treatment patterns necessitate the examination of healthcare resource use and costs. Therefore, we investigated trends in total healthcare costs from 2005 to 2014 among prevalent and incident patients with HF.[8] Based on the frequency and duration of hospitalizations, number of outpatient visits and associated direct costs of these events, and costs associated with primary care and prescription medications. Patient characteristics in the context of resource use were analyzed to determine which individuals incurred the greatest resource and secondary care costs. With the growing importance of HF with preserved ejection fraction (HFpEF), this investigation goes a step further than other Swedish studies[3, 4] to present new data comparing resource use and costs among patients according to HF phenotype.

Methods

Study design

This was a retrospective, non-interventional cohort study using longitudinal patient-level data for patients with HF in Sweden.[9] Data were collected from electronic medical records (EMRs) from primary and secondary care and from local echocardiography registries in the counties of Uppsala (two hospitals and 46 primary care centres [PCCs]) and Västerbotten (three hospitals and 37 PCCs). EMR data were linked via unique identifiers issued by the

For peer review only

National Board of Health and Welfare (NBHW) to data from national health registers, including the National Patient Register (NPR). Data were pseudonymized before the linked database was released to the research group, and ethical approval was obtained from the regional Ethical Review Board in Uppsala, Sweden (2015-045) before data extraction. No informed consent was required for this retrospective, pseudonymized study.

Patients

Patients (aged ≥18 years) with primary or secondary diagnoses of HF during the identification period of January 2005 to March 2015 were included. Analyzing data from patients treated in both primary and secondary care by linking NPR data with EMR data presents the challenge of distinct HF diagnosis specificity across the two settings. Two HF diagnoses were required to reduce the uncertainty surrounding HF diagnosis in primary care and increase overall diagnosis specificity. Diagnosis of HF was based on the International Classification of Diseases and Related Health Problems, tenth revision (ICD-10) diagnostic codes of I50, I42.0, I42.6, I42.7, I42.9, I11.0, I13.0, or I13.2 for the primary or secondary diagnoses (Table S1).

The national cohort comprised prevalent patients with HF registered in the NPR from secondary care between January 2005 and December 2014, while the regional cohort (Uppsala and Västerbotten) included incident patients with HF diagnoses registered in EMRs from either primary or secondary care between January 2010 and March 2015.

The Pygargus Customized eXtraction Program (CXP 3.0) extracted data from EMRs from Uppsala and Västerbotten and subsequently linked to data from the national health registers including the NPR.[10] HF with preserved ejection fraction [HFpEF] and HF with reduced ejection fraction [HFrEF]) was determined based only on data from local echocardiography registries, and for cohort 2, HFpEF was defined as a left ventricular ejection fraction (LVEF) of ≥50% and HFrEF was defined as an LVEF of <50%. This cut-off

For peer review only

is based on data constraints; LVEF is recorded categorically in the echocardiography registers, with the categories used differing between the county councils such that 50% was the only option that allowed for a common threshold.

Data extraction and study timelines

Data were extracted from EMRs based on an observed HF diagnosis during 1994-2015 for the Uppsala County and during 1992–2016 for the Västerbotten County. The analysis period was adjusted to 1 January 2010 owing to inconsistent patterns of HF registration in the Uppsala County EMR systems. The look-back period in the EMR data was from the time the first data were available until 31 December 2009. For NPR data, the look-back period was from 1 January 1997 for inpatient care and 1 January 2001 for outpatient care to 31 December 2004 (Figure S1) to identify prevalent patients with HF at a national level. This prevalent patient cohort was used to assess trends in total healthcare resource use and costs from 2005 to 2014.

Newly diagnosed individuals from the Uppsala County and during the analysis period made up the incident HF population. To enable all patients to have at least 3 years of potential follow-up, all analyses were performed on patients diagnosed with incident HF between 2010 and 2012 (Figure S1).

For the analyses of clinical characteristics, healthcare resource use, and costs, the index date was defined as the date of first HF diagnosis. However, for the analyses of hospitalizations, the index date was defined as a patient’s second HF diagnosis to improve the accuracy of diagnosis and minimize the effects of immortal time bias. Follow-up was defined as the period between the second HF diagnosis and the end of the study, end of EMR collection for those patients who moved to another region, or date of death, whichever came first.

For peer review only

Variables analyzed and statistical analyses

For categorical outcomes a χ^2 test was performed; for comparison of means, a *t*-test was performed and confidence intervals (CIs) were calculated at a 95% level. Descriptive statistics assessed baseline clinical characteristics (Table 1) on the date of first HF diagnosis, since this was truly the first time that the patients were considered to have HF. Comorbidities, data from primary (EMR) and secondary (EMR and NPR) care stratified according to HF phenotype, were summarized according to the ICD-10 codes (Table S1). Comorbidities were collected from visits that occurred 0–5 years before the date of the first HF diagnosis. Charlson comorbidity index (CCI) scores during this time, ranging from 0 to greater than 10 (higher scores indicate greater comorbidity), were calculated.

All-cause CVD-related hospitalizations were analyzed using a subdistribution hazard model, proportion of patients having all-cause CVD-related hospitalizations occurring within 0.5, 1 and 3 years after the second HF diagnosis were estimated by a cumulative incidence function.. The time to all-cause and time to CVD-related hospitalizations/readmissions were estimated from the date of the second HF diagnosis using a Cox proportional hazards model, with age group, sex, diagnosis setting (primary care, secondary care, unknown), HF phenotype, and N-terminal pro B-type natriuretic peptide (NT-proBNP) level (0–1,000, 1,001–3,000, and >3,000 pg/mL) as covariates. Data for the mean and median (interquartile range [IQR]) number of hospitalizations and outpatient visits for all-cause, CVD-related, and HF-related events, as well as mean length of stay (LOS), were analyzed by HF phenotype and year since HF diagnosis.

Costs were estimated by year since the first HF diagnosis. All costs are presented as SEK 2015 values (based on the Consumer Price Index,[11] with the equivalent value in euros [€], based on the historical exchange rate in January 2015 (1 SEK = €0.105). Analyses of all-cause, CVD-related, and HF-related costs associated with secondary care were performed

For peer review only

based on diagnosis-related group codes and price lists, as determined by the NBHW.[12] Costs associated with primary care comprised costs of family physician and nurse visits as well as costs of blood tests. Primary care costs were based on general price lists and were available for the Uppsala County only, as were costs related to pharmacotherapy.[13] The total cost of all drug use for incident patients with HF was estimated, as was the aggregated cost of HF-related drugs, based on the 2012 European Society of Cardiology (ESC) guidelines (Table S2).[14] Logistic regression assessed baseline characteristics of patients who incurred the greatest secondary care costs related to HF, defined as the top 10% most resource-intensive patients.

SAS version 9.3 or higher was used for statistical analysis and data management.

Results

National prevalent population with HF

The absolute number of prevalent patients with HF in Sweden increased by 1.5-fold between 2005 and 2014 from 89,837 to 133,220 (overall population increased from 7,113,513 to 7,762,073), with a corresponding increase in age-adjusted prevalence from 1.30% to 1.72%.

Increase in total costs of prevalent patients with HF over time.

The total number of hospitalizations, hospital days, emergency room (ER) visits, and outpatient visits in secondary care increased from 2005 to 2014 by between 1.2- and 2.2-fold (Figure S2). The number of all-cause hospitalizations increased from 141,941 to 181,374 (difference: 39,433; 28% increase). The number of hospital days increased from 998,512 to 1,165,310 (difference: 166,798; 17% increase), indicating a decreased mean LOS of 4.23 days per hospitalization. ER visits increased from 14,272 to 31,037 (difference: 16,765;

For peer review only

117% increase). Outpatient visits in secondary care increased from 262,771 to 497,230 (difference: 234,459; 89% increase).

The total annual costs associated with the secondary care of prevalent patients with HF increased by 1.4-fold from SEK 6.23 billion (€0.60 billion) in 2005 to SEK 8.86 billion (€0.85 billion) in 2014, mainly driven by a corresponding increase in the overall resource use in inpatient care (52%) (Figure S3a). On an average, inpatient and outpatient costs accounted for 84% and 16% of the total costs, respectively. On an average, 81% of the total costs of secondary care were attributed to CVD-related costs; 64% of these were attributed to HF-related costs (Figure S3b). HF-related costs accounted for 52% of the total costs.

Regional incident population with HF

Data from 104,562 patients were identified from the Uppsala and Västerbotten counties; among these, 8,702 (8.3%) patients had a defined HF diagnosis. Information was available for 4,648 incident patients with HF between 2010 and 2012 (Figure S1). HF phenotype was characterized for 1,715 patients (36.9%); 1,106 (64.5%) patients had HFrEF and 609 (35.5%) had HFpEF (Figure S1).

Patient demographics and clinical characteristics

Table 1 presents the baseline clinical characteristics of patients, overall and by HF phenotype. Mean (median, IQR) age of the patients was 76.8 (79.0, 69.5–86.7) years, more than one-quarter (27.8%) were aged 85 years or more, and 53.8% were male. Most first HF diagnoses occurred in secondary care (75.9%) rather than in primary care (24.1%) (Table 2).

Hospitalizations

All-cause and CVD-related hospitalizations

For peer review only

The cumulative incidence function (95% CI) of the proportion of patients with all-cause hospitalizations within the first year of HF diagnosis was 64.7% (60.8–68.4) for patients with HFrEF and 63.7% (60.8–66.5) for patients with HFpEF; the corresponding sub-distribution hazard ratio (HR) over the follow-up period for HFrEF compared with HFpEF was 0.91 (95% CI: 0.81–1.03; P=0.1404) (Table S3). The cumulative incidence function of the proportion of patients with all-cause hospitalizations was similar for patients who had their first HF diagnosis in secondary vs primary care; the corresponding sub-distribution HR for the follow-up period for secondary vs primary care was 0.93 (95% CI: 0.86–1.01; P<0.0675) (Table S3). Higher NT-proBNP levels were associated with a greater risk of all-cause hospitalizations (Table S3), also seen for the proportion of patients with CVD-related hospitalizations by HF phenotype, diagnosis setting, NT-proBNP level, and age (Table 2).

Number of hospitalizations and outpatient visits and LOS

Mean and median number of all-cause, CVD-related, and HF-related hospitalizations and outpatient visits per patient decreased from the first year of HF diagnosis and stabilized between years 2 and 3 (Figure 1). Similar trends in hospitalizations and outpatient visits compared with those observed in the overall population were observed for patients with HFrEF and HFpEF, except that in patients with HFpEF, the mean number of CVD-related outpatient visits increased from the first year after HF diagnosis (Figure 1). The mean (median, IQR) LOS for all-cause, CVD-related, and HF-related hospitalizations was 17.5 (8, 2–23), 16.5 (8, 2–22), and 12.8 (6, 0–16) days in the first year after HF diagnosis, declining at 3 years after diagnosis to 7.6 (0, 0–8), 7.0 (0, 0–7), and 4.2 (0, 0–2) days, respectively. Patients with HFrEF had a shorter LOS for all-cause, CVD-related, and HF-related events than those with HFpEF (data not shown).

For peer review only

Healthcare costs per patient for the first year after the index date

Secondary healthcare (overall, inpatient, and outpatient) costs

Total and inpatient all-cause costs decreased by more than half after the first year of HF diagnosis (Figure 2). A reduction in all-cause outpatient costs was also noted at the second year after HF diagnosis, relative to the first year. Similar trends were seen for CVD-related and HF-related costs. In the first year after HF diagnosis, total all-cause secondary care costs were SEK 122,758 (€12,890) per patient per year, which declined to SEK 53,220 (€5,588) per patient per year by year 2 after diagnosis. The corresponding total CVD-related and HF-related costs were SEK 110,268 (€11,578) and SEK 84,956 (€8,920) per patient per year in the first year, which decreased to SEK 43,590 (€4,577) and 26,038 (€2,734) per patient per year by year 2, respectively. Therefore, HF-specific inpatient and outpatient costs accounted for 69% of the total costs in the first year after HF diagnosis, declining to 49% during the second year after diagnosis (and 46% during the third year after diagnosis). Inpatient care constituted more than 90% of the total secondary healthcare costs per patient at 1 year after diagnosis for all-cause (SEK 112,432 [€11,805]), CVD-related (SEK 106,101 [€11,141]), and HF-related (SEK 82,897 [€8,704]) events. By comparison, the mean total primary care cost for the Uppsala County cohort only was SEK 10,347 (€1,086) per patient per year (Table S4; Supplementary Information).

Drivers of high resource use and costs

Of the total patient population, 10% (n=465) incurred approximately 40% of the total secondary healthcare costs. Compared with ‘other’ (non-resource intensive) patients, those in the resource-intensive population were likely to have a lower mean age, belong to the male sex, have a mean NT-proBNP level above 3,000 pg/mL, and a number of comorbidities; had

For peer review only

a known LVEF; and had not received either an angiotensin-converting enzyme inhibitor or angiotensin receptor blocker (Table 3).

Discussion

This study found that (i) the absolute number of patients with HF in Sweden continues to increase; (ii) the total cost of secondary care of prevalent patients with HF increased and was primarily driven by costs associated with inpatient care; (iii) patients with newly diagnosed HF were at a high risk of frequent hospitalizations during the first year after diagnosis, especially in those diagnosed in secondary vs primary care; (iv) HFpEF was associated with greater resource use compared with HFrEF or an unknown LVEF; (v) during the first year, hospitalizations (inpatient care) constituted more than 90% of the total secondary healthcare costs, with 69% of all secondary healthcare costs being attributable to HF-specific care; and (iv) approximately 10% of the patients were highly resource-intensive.

In HF management, hospitalizations are a key challenge because they are highly resource intensive and costly.[3, 4, 15-17] Not only are patients with HF frequently hospitalized, but duration of their admissions are also often lengthy, particularly in Europe compared with the US.[(15, 16)] In the current study, the mean hospital LOS during the first year after HF diagnosis was more than 16 days for patients admitted for all-cause or CVD-related events and 12.8 days for patients admitted for HF-related events.

The clinical implication of our data is to identify factors responsible for the high costs of HF within the first year of diagnosis. Such factors could include patients being discharged from hospital too early to ensure full evaluation, patients not being initiated on evidence-based therapy or devices, delayed or incomplete follow-up in patient management programmes, and loss of continuity and/or ineffective HF follow-ups.

For peer review only

An important observation concerned the risk of hospitalization/readmission over the follow-up period, which was significantly greater for patients receiving their first HF diagnosis in secondary vs primary care. This might be the result of patients presenting with more severe HF and/or more severe chronic HF and patients with a diagnosis of acute HF. Accordingly, the total costs associated with primary care, available for the Uppsala County only, were SEK 10,347 (€1,086) per patient and were much lower than the secondary care costs in the first year after diagnosis. These costs generally remained stable over the first 4 years of follow-up and increased slightly at year 5, which could be a result of the increasing age of the population.

Patients with HFpEF were also at a higher risk of all-cause and CVD-related hospitalizations and required more outpatient visits at 3 years than those with HFrEF, potentially because patients with HFpEF tended to be older and/or have a higher comorbidity burden. Importantly, a higher proportion of deaths in patients with HFpEF than in those with HFrEF may have a non-CVD cause.[18, 19] Mortality data presented in Lindmark et al[(20)] show that the HR for the 1-year all-cause mortality rate was significantly lower for HFrEF than for HFpEF (0.77, 95% CI 0.62–0.96, P=0.0159).

We found a decline in resource use observed after the first year of diagnosis and substantial reductions in healthcare costs, with total all-cause costs per patient decreasing by more than half in the second year. This decrease reflects patient-level, longitudinal data trends and, in fact, from a total population/healthcare system perspective, the opposite was true with an increase in costs over time (Table S5) because of an increasing prevalence of HF and a shift in patient profile towards older and sicker patients.[20] In our incident population, the high financial costs during the first year after diagnosis may be partly explained by survivor bias, assuming that patients alive in later years have less severe HF symptoms and require less cost-intensive treatment, such as the need for re-hospitalizations and longer LOS.

For peer review only

Other possible explanations include the use of both more intensive investigations to define the aetiology of HF during the first year and expensive medicines, devices, and/or surgical treatments during the initial phase of treatment. The costs reported herein are generally similar to those reported in another Swedish EMR- and register-based study that analyzed data from 2006.[4]

Similar to other economic studies in HF,[3, 21, 22] the inpatient care accounted for most of the total secondary care costs (~90%) and showed the greatest proportional decline in costs from the peak at 1 year after diagnosis, consistent with the reduction seen in the number of hospitalizations. Similar trends were observed for CVD-related and HF-related costs per patient. The comparatively lower HF-related costs suggest that patients accrue significant costs related to conditions inherent to HF. Data for the 10% of patients who accumulated the highest healthcare costs showed that patients with several different comorbidities were associated with a higher resource use. The most resource-intensive patients tended to be male with high NT-proBNP levels. Patients with high/increasing biomarker levels tend to be followed up closely by specialists because of the potential for adverse outcomes.[23]

A limitation of the current study was the challenge of retrospectively identifying patients with confirmed HF despite the requirement for at least two HF diagnoses. Because the primary care EMR systems did not implement a hierarchy of diagnoses, this condition could not be applied. The use of two, rather than one, HF diagnoses was expected to increase the certainty surrounding the HF diagnosis, although it could potentially have led to exclusion of more recent or mild cases of HF. Second limitation concerned the definition of subgroups based on the HF phenotype. The limited number of echocardiographs required for the verification of the HF diagnosis was a major drawback, meaning that a large proportion of patients had missing information for both LVEF and structural and functional pathologies. This may have introduced some level of bias because it could be assumed that the

For peer review only

echocardiography was performed only in more severe cases of HF, meaning that the two HF phenotype subgroups were not wholly representative of the total cohort. Nevertheless, our data reflect the real-world costs associated with a diagnosis of HF, irrespective of the true validation of the diagnosis. Moreover, the threshold for defining HFrEF was an LVEF of ≤50%, which is higher than the recommendations from the ESC of <40%[24]; therefore, a larger number of patients would have been categorized as having HFrEF. Finally, there were missing data on doctor visits for the Västerbotten County cohort because of technical issues relating to the Västerbotten EMR database; thus, primary care resource use and costs were available only for the Uppsala County cohort.

Conclusions

Care for patients with HF represents an increasing financial burden on the Swedish healthcare system. Hospitalizations/readmissions represent a key resource and cost burden regardless of HF phenotype, particularly during the first year after diagnosis, with total costs of HF attributed primarily to those associated with inpatient care. Furthermore, characteristics of patients incurring the greatest healthcare costs included younger age, male sex, and high CCI scores and NT-proBNP levels at the time of the HF diagnosis. Understanding the profile of patients with HF who are likely to incur the greatest healthcare costs is invaluable to guide resource allocation.

Acknowledgements

The authors thank Japinder Kaur of Novartis and Sharon Smalley and Carly Sellick of Pharma Genesis, London, for providing medical writing assistance with this manuscript.

Funding This research was funded by Novartis Pharma AG, Basel, Switzerland.

For peer review only

Competing interest K. Boman, M. Olofsson, and J. Stålhammar received reimbursement from Novartis via IQVIA for performing the study. K. Boman and M. Olofsson have also received lecture grants from Novartis. K. Lindmark received lecture grants and consultant fees from Novartis. G. Wikström has no conflicts of interest to declare; however, Uppsala University received research funding from Novartis for conducting this study. M. Costa-Scharplatz is an employee of Novartis Sweden AB, Sweden, and A.F. Fonseca is an employee of Novartis Pharma AG, Basel, Switzerland. Data extraction and analysis were conducted by S. Johansson, V. Heller, and M. Törnblom are employees of IQVIA, Sweden, which was commissioned to conduct the study on behalf of Novartis Pharma AG, Basel, Switzerland, and have ongoing consulting and research relationships with Novartis Pharma AG.

Data availability statement Major findings from the study will be published in scientific manuscripts only. The data will not be made available in any other format in order to preserve the privacy of the patients in compliance with local laws and regulation.

Authors contributions

Kurt Boman and Gerhard Wikström were involved in the study and manuscript conceptualization, interpretation of results and development of the first draft of the manuscript. Krister Lindmark contributed to protocol development, study and manuscript conceptualization, results interpretation, refinement of analysis requirements and writing of the manuscript. Stina Johansson, Vincent Heller, Michael Törnblom performed statistical analyses. Jan Stålhammar and Mona Olofsson contributed to protocol development, study conceptualization, interpretation of results and supported the development of the manuscript. Madlaina Costa-Scharplatz and Ana Filipa Fonseca contributed to manuscript conceptualization, interpretation of results, refinement of analysis requirements and supported the development of the manuscript. All authors reviewed the manuscript and approved the final draft of the manuscript for submission.

For peer review only

References

1. Bui AL, Horwich TB, Fonarow GC. Epidemiology and risk profile of heart failure. *Nat Rev Cardiol*. 2011;**8**(1):30-41.

2. Socialstyrelsen (The National Board of Health and Welfare). Statistics on the causes of death 2015. https://www.socialstyrelsen.se/globalassets/sharepoint-dokument/artikelkatalog/statistik/2016-8-3.pdf 2016 [Accessed on 19 December 2017]

3. Stalhammar J, Stern L, Linder R, Sherman S, Parikh R, Ariely R, Deschaseaux C, Wikstrom G. The burden of preserved ejection fraction heart failure in a real-world Swedish patient population. *J Med Econ*. 2014;**17**(1):43-51.

4. Stalhammar J, Stern L, Linder R, Sherman S, Parikh R, Ariely R, Wikstrom G. Resource utilization and cost of heart failure associated with reduced ejection fraction in Swedish patients. *J Med Econ*. 2012;**15**(5):938-46.

5. Sveriges Kommuner och Landsting (SKL) SKoL. Socialstyrelsen. Öppna jämförelser 2014. Hälso-och sjukvård. Jämförelser mellan landsting. Del 2. Indikatorer om sjukdomar och behandlingar. **https://webbutik.skr.se/bilder/artiklar/pdf/7585-157-0.pdf?issuusl=ignore.** 2014 [Accessed on 2 February 2018].

6. Agvall B, Borgquist L, Foldevi M, Dahlstrom U. Cost of heart failure in Swedish primary healthcare. *Scand J Prim Health Care*. 2005;**23**(4):227-32.

7. Ryden-Bergsten T, Andersson F. The health care costs of heart failure in Sweden. *J Intern Med*. 1999;**246**(3):275-84.

8. Boman K LK, Stalhammar J, Olofsson M, Costa-Scharplatz M, Fonseca AF, Bruce Wirta S, Castelo-Branco A, Tornblom M, Wikstrom G. Total annual healthcare costs of heart

For peer review only

failure between 2005 and 2014: a retrospective, population-based study in Sweden. . Poster presented at the European Meeting of the *International Society for Pharmacoeconomics and Outcomes Research* (ISPOR) 2018;10–14 November 2018, Barcelona, Spain.

9. Heidenreich PA AN, Allen LA, Bluemke DA, Butler J, Fonarow GC, Ikonomidis JS, Khavjou O, Konstam MA, Maddox TM, Nichol G, Pham M, Pina IL, Trogdon JG, American Heart Association Advocacy Coordinating C, Council on Arteriosclerosis T, Vascular B, Council on Cardiovascular R, Intervention, Council on Clinical C, Council on E, Prevention, Stroke C. Forecasting the impact of heart failure in the United States: a policy statement from the American Heart Association. *Circ Heart Fail* 2013;**6**:606–19.

10. Martinell M, Jan Stålhammar, J., Hallqvist, J. Automated data extraction—A feasible way to construct patient registers of primary care utilization. *Upsala Journal of Medical Sciences*. 2012;**117**:52-6.

11. Statistics Sweden. Consumer price index [Internet]. **http://www.scb.se/en/finding-statistics/statistics-by-subject-area/prices-and-consumption/consumer-price-index/consumer-price-index-cpi/**. 2016 [Accessed on 12 January 2016].

12. National Board of Health and Welfare. 2015 Vägledning till NordDRG. Swedish version. **https://www.socialstyrelsen.se/globalassets/sharepoint-dokument/artikelkatalog/ovrigt/2015-12-29.pdf**

. 2016 [Accessed 2 February 2018].

13. Tandvårds- och läkemedelsförmånsverket TTSDaPBA. **https://www.tlv.se/in-english/prices-in-our-database.html.** [Accessed 5 March 2018].

14. McMurray JJ, Adamopoulos S, Anker SD, Auricchio A, Bohm M, Dickstein K, Falk V, Filippatos G, Fonseca C, Gomez-Sanchez MA, Jaarsma T, Køber L, Lip GYH, Maggioni AP, Parkhomenko A, Pieske BM, Popescu BA, Rønnevik PK, Rutten FH, Schwitter J, Seferovic P, Stepinska J, Trindade PT, Voors AA, Zannad F, Zeiher A, Bax JJ, Baumgartner

For peer review only

H, Ceconi C, Dean V, Deaton C, Fagard R, Funck-Brentano C, Hasdai D, Hoes A, Kirchhof P, Knuuti J, Kolh P, McDonagh T, Moulin C, Popescu BA, Reiner Z, Sechtem U, Sirnes PA, Tendera M, Torbicki A, Vahanian A, Windecker A, McDonagh T, Sechtem U, Bonet LA, Avraamides P, Ben Lamin HA, Brignole M, Coca A, Cowburn P, Dargie H, Elliott P, Flachskampf FA, Guida GF, Hardman S, Lung B, Merkely B, Mueller C, Nanas JN, Nielsen OW, Ørn S, John T. Parissis JT, Ponikowski P. ESC Guidelines for the diagnosis and treatment of acute and chronic heart failure 2012: The Task Force for the Diagnosis and Treatment of Acute and Chronic Heart Failure 2012 of the European Society of Cardiology. Developed in collaboration with the Heart Failure Association (HFA) of the ESC. *Eur Heart J*. 2012;**33**(14):1787-847.

15. Cowie MR, Anker SD, Cleland JGF, Felker GM, Filippatos G, Jaarsma T, Jourdain P, Knight E, Massie B, Ponikowski P, López-Sendón J. Improving care for patients with acute heart failure: before, during and after hospitalization. *ESC Heart Fail*. 2014;**1**(2):110-45.

16. Braunschweig F, Cowie MR, Auricchio A. What are the costs of heart failure? *Europace*. 2011;**13** Suppl 2:ii13-7.

17. Kilgore M, Patel HK, Kielhorn A, Maya JF, Sharma P. Economic burden of hospitalizations of Medicare beneficiaries with heart failure. *Risk Manag Healthc Policy*. 2017;**10**:63-70.

18. Dunlay SM, Redfield MM, Weston SA, Therneau TM, Hall Long K, Shah ND, Roger VL. Hospitalizations after heart failure diagnosis a community perspective. *J Am Coll Cardiol*. 2009;**54**(18):1695-702.

19. Chan MM, Lam CS. How do patients with heart failure with preserved ejection fraction die? *Eur J Heart Fail*. 2013;**15**(6):604-13.

20. Lindmark K, Boman K, Olofsson M, Tornblom M, Levine A, Castelo-Branco A, Schlienger R, Wirta SB, Stålhammar J, Wikström G. Epidemiology of heart failure and

For peer review only

trends in diagnostic work-up: a retrospective, population-based cohort study in Sweden. *Clin Epidemiol*. 2019;**11**:231-44.

21. Stafylas P, Farmakis D, Kourlaba G, Giamouzis G, Tsarouhas K, Maniadakis N, Parissis J. The heart failure pandemic: The clinical and economic burden in Greece. *Int J Cardiol*. 2017;**227**:923-9.

22. Mejhert M, Lindgren P, Schill O, Edner M, Persson H, Kahan T. Long term health care consumption and cost expenditure in systolic heart failure. *Eur J Intern Med*. 2013;**24**(3):260-5.

23. Kim HN, Januzzi JL, Jr. Natriuretic peptide testing in heart failure. *Circulation*. 2011;**123**(18):2015-9.

24. Ponikowski P, Voors AA, Anker SD, Bueno H, Cleland JGF, Coats AJS, Falk V, González-Juanatey JR, Harjola V, Jankowska EA, Jessup M, Linde C, Nihoyannopoulos P, Parissis JT, Pieske B, Riley JP, Rosano GMC, Ruilope LM, Ruschitzka F, Rutten FH, Meer P. 2016 ESC Guidelines for the diagnosis and treatment of acute and chronic heart failure: The Task Force for the diagnosis and treatment of acute and chronic heart failure of the European Society of Cardiology (ESC)Developed with the special contribution of the Heart Failure Association (HFA) of the ESC. *Eur Heart J*. 2016;**37**(27):2129-200.

For peer review only

Tables

Table 1 Demographic and clinical characteristics at baseline for incident patients with HF from the Uppsala and Västerbotten counties with a first HF diagnosis in 2010–2012, overall and by HF phenotype

Characteristic	Overall population (N=5,205)	HF phenotype HFpEF (n=652)	HFrEF (n=1,167)	Unknown LVEF (n=3,386)
Mean age at index date (SD), years	76.8 (12.3)	74.8 (12.2)	70.3 (13.0)	79.4 (11.1)
Median age at index date (IQR), years	79.0 (69.5, 85.7)	76.8 (68.3, 83.6)	71.2 (63.1, 80.3)	81.5 (73.4, 87.2)
Sex, n (%)				
Women	2,405 (46.2)	341 (52.3)	380 (32.6)	1,684 (49.7)
Men	2,800 (53.8)	311 (47.7)	787 (67.4)	1,702 (50.3)
Mean NT-proBNP level (SD), pg/mL	4,909.1 (8,202.9)	4,270.8 (7,576.7)	6,217.1 (9,495.2)	4,543.4 (7,730.5)
Mean SBP/DBP (SD), mmHg	139.7 (25.6)/ 78.7 (15.3)	141.8 (27.1)/ 76.8 (14.6)	134.9 (24.3)/ 79.7 (15.3)	140.9 (25.6)/ 78.8 (15.3)
Mean eGFR (SD), mL/min/1.73 m^2	51.9 (29.1)	49.6 (27.9)	60.6 (30.2)	44.2 (26.0)
Mean CCI[a,b] (SD)	1.8 (2.2)	1.8 (2.2)	1.7 (2.1)	1.9 (2.2)
Common comorbidities and risk factors, n (%)[b,c]				
Hypertension	2,679 (51.5)	366 (56.1)	476 (40.8)	1,837 (54.3)

For peer review only

Atrial fibrillation	1,593 (30.6)	212 (32.5)	247 (21.2)	1,134 (33.5)
IHD (angina or MI)	1,198 (23.0)	122 (18.7)	303 (26.0)	773 (22.8)
Diabetes	952 (18.3)	137 (21.0)	197 (16.9)	618 (18.3)
Cancer	739 (14.2)	118 (18.1)	141 (12.1)	480 (14.2)
Dyslipidaemia	676 (13.0)	89 (13.7)	152 (13.0)	435 (12.8)
Cerebrovascular disease	660 (12.7)	70 (10.7)	109 (9.3)	481 (14.2)
Anaemia	645 (12.4)	104 (16.0)	101 (8.7)	440 (13.0)

CCI, Charlson comorbidity index; DBP, diastolic blood pressure; eGFR, estimated glomerular filtration rate; HF, heart failure; HFpEF, HF with preserved ejection fraction; HFrEF, HF with reduced ejection fraction; IHD, ischaemic heart disease; IQR, interquartile range; LVEF, left ventricular ejection fraction; MI, myocardial infarction; NT-pro-BNP, N-terminal pro-B-type natriuretic peptide; SBP, systolic blood pressure; SD, standard deviation.

Notes: Data are either number (n) and percentage (%) or mean (SD). [a]Includes patients with a CCI of zero (ie no comorbidities). [b]Comorbidities and underlying cardiac diseases occurring 0–5 years before the index date. [c]Comorbidities and underlying cardiac diseases occurring in ≥10% of the overall population.

For peer review only

Table 2 Sub-distribution hazard model for the proportion of incident patients with HF from the Uppsala and Västerbotten counties with a second HF diagnosis in 2010–2012 who had CVD-related hospitalizations within 3 years and cumulative incidence function estimates for the proportion of patients hospitalized within 0.5, 1, and 3 years after index date

Characteristic	N=4,648	Cumulative incidence function of CVD-related hospitalizations, % (95% CI)			Sub-distribution HR (95% CI)	*P* value
		0.5 years	1 year	3 years		
Age group, years						
18–54 (Reference)	275	46.9 (40.9–52.7)	52.0 (45.9–57.7)	62.5 (55.9–68.3)	1	
55–64	457	47.0 (42.4–51.5)	56.5 (51.8–60.9)	71.8 (66.7–76.3)	1.15 (0.95–1.41)	0.1564
65–74	1,051	47.3 (44.2–50.3)	57.5 (54.4–60.4)	72.5 (69.2–75.4)	1.21 (1.01–1.45)	0.0383
75–84	1,592	44.6 (42.1–47.0)	55.3 (52.9–57.7)	75.6 (72.8–78.0)	1.21 (1.02–1.45)	0.0305
≥85	1,273	41.9 (39.2–44.6)	52.2 (49.4–54.9)	70.7 (67.8–73.4)	1.13 (0.94–1.35)	0.1916
Sex						
Female (Reference)	2,143	43.8 (41.7–45.9)	52.9 (50.8–55.0)	71.7 (69.4–73.9)	1	
Male	2,505	45.8 (43.8–47.7)	56.5 (54.6–58.4)	73.0 (70.9–74.9)	1.06 (0.98–1.14)	0.1211
Diagnosis setting						
Primary care (Reference)	1,118	41.8 (38.9–44.6)	53.3 (50.3–56.2)	77.1 (73.8–80.0)	1	
Secondary care	3,528	45.8 (44.2–47.4)	55.3 (53.7–57.0)	71.0 (69.3–72.7)	0.94 (0.86–1.02)	0.1178
Unknown	2	N/A	N/A	N/A	1.99 (1.32–3.00)	0.0011
HF phenotype						

For peer review only

HFrEF (Reference)	609	53.2 (49.2–57.1)	61.1 (57.1–64.8)	77.9 (73.7–81.5)	1	
HFpEF	1,106	52.4 (49.5–55.3)	60.9 (58.0–63.7)	73.8 (70.8–76.6)	0.93 (0.83–1.06)	0.2769
Unknown LVEF	2,933	40.3 (38.5–42.0)	51.3 (49.5–53.1)	70.9 (68.9–72.8)	0.79 (0.71–0.88)	<0.0001
NT-proBNP level, pg/mL						
0–1,000 (reference)	912	40.4 (37.2–43.5)	51.8 (48.5–54.9)	72.4 (68.8–75.7)	1	
1,001–3,000	1,283	45.4 (42.7–48.1)	56.6 (53.8–59.2)	76.1 (73.1–78.7)	1.10 (0.99–1.21)	0.0682
>3,000	1,601	52.5 (50.0–54.9)	61.3 (58.8–63.6)	74.3 (71.7–76.7)	1.19 (1.08–1.32)	0.0006
Missing	852	34.5 (31.3–37.7)	43.5 (40.2–46.8)	63.2 (59.4–66.9)	0.83 (0.74–0.93)	0.0017

CI, confidence interval; CVD, cardiovascular disease; HF, heart failure; HFpEF, HF with preserved ejection fraction; HFrEF, HF with reduced ejection fraction; HR, hazard ratio; LVEF, left ventricular ejection fraction; NT-proBNP, N-terminal pro-B-type natriuretic peptide.

Note: The Fine and Grays sub-distribution hazard model was used to obtain the sub-distribution HRs. Cumulative incidence function was used to estimate percentage of patients with CVD-related hospitalizations within 0.5, 1, and 3 years after index date.

For peer review only

Table 3 Logistic regression to determine characteristics at HF diagnosis of the 10% of incident patients with HF incurring the greatest secondary healthcare costs during the first year after index date

		**Unadjusted model**		**Adjusted model**	
**Characteristics at HF diagnosis**	**N=5,205 n (%)**	**OR**	**95% CI**	**OR**	**95% CI**
Age at index date, years					
18–54	297 (5.7%)	Reference		Reference	
55–64	511(9.8%)	0.88	0.60–1.29	0.83	0.56–1.24
65–74	1,155 (22.2%)	0.89	0.64–1.24	0.82	0.57–1.18
75–84	1,796 (34.5%)	0.40	0.28–0.57	0.39	0.27–0.57
≥85	1,446 (27.8%)	0.18	0.12–0.27	0.21	0.13–0.33
Sex					
Male	2,800 (53.8%)	Reference		Reference	
Female	2,405 (46.2%)	0.73	0.61–0.88	0.98	0.80–1.20
LVEF					
HFrEF	1,167 (22.4%)	Reference		Reference	
HFpEF	652 (12.5%)	1.01	0.79–1.30	1.05	0.80–1.39
Unknown	3,386 (65.1%)	0.29	0.24–0.36	0.41	0.33–0.52
NT-proBNP, ng/L					
0–1,000	1,075 (20.7%)	Reference		Reference	
1,001–3,000	1,433 (27.5%)	1.17	0.88–1.56	1.48	1.09–2.01
>3,001	1,730 (33.2%)	1.77	1.36–2.30	1.93	1.45–2.57
Missing	967 (18.6%)	1.17	0.85–1.60	1.44	1.03–2.02

For peer review only - http://bmjopen.bmj.com/site/about/guidelines.xhtml

ACEI or ARB treatment					
ACEI	2,481 (47.7%)	Reference		Reference	
ACEI and ARB	58 (1.1%)	1.81	0.88–3.74	1.61	0.75–3.45
ARB	964 (18.5%)	1.02	0.79–1.31	1.01	0.77–1.33
Neither ACEI nor ARB	1,702 (32.7%)	1.26	1.03–1.55	1.63	1.31–2.03
Hypertension					
No	2,526 (48.5%)	Reference		Reference	
Yes	2,679 (51.5%)	1.11	0.93–1.33	1.06	0.84–1.33
Atrial fibrillation					
No	3,612 (69.4%)	Reference		Reference	
Yes	1,593 (30.61)	0.75	0.61–0.92	0.77	0.61–0.97
Angina or MI					
No	4,007 (76.9%)	Reference		Reference	
Yes	1,198 (23.02%)	1.45	1.19–1.78	1.27	1.00–1.60
Diabetes					
No	4,253 (81.71%)	Reference		Reference	
Yes	952 (18.29%)	1.48	1.19–1.83	1.19	0.93–1.53
Cancer					
No	4,466 (85.80%)	Reference		Reference	
Yes	739 (14.20%)	1.19	0.93–1.52	1.17	0.89–1.53
Cerebrovascular disease					
No	4,545 (87.32%)	Reference		Reference	
Yes	660 (12.68%)	1.08	0.83–1.41	1.27	0.95–1.71

For peer review only - http://bmjopen.bmj.com/site/about/guidelines.xhtml

Dyslipidaemia					
No	4,529 (87.01%)	Reference		Reference	
Yes	676 (12.99%)	1.60	1.26–2.03	1.10	0.83–1.47
Anaemia					
No	4,560 (87.61%)	Reference		Reference	
Yes	645 (12.39%)	1.73	1.36–2.20	1.63	1.24–2.14
COPD					
No	4,801 (92.24%)	Reference		Reference	
Yes	404 (7.76%)	1.27	0.93–1.74	1.13	0.80–1.58
Aortic insufficiency/regurgitation					
No	4,814 (92.49%)	Reference		Reference	
Yes	391 (7.51%)	3.21	2.49–4.13	2.54	1.92–3.37
Dementia					
No	4,883 (93.81%)	Reference		Reference	
Yes	322 (6.19%)	0.33	0.19–0.60	0.53	0.29–0.99
Chronic kidney disease					
No	4,987 (95.81%)	Reference		Reference	
Yes	218 (4.19%)	2.47	1.76–3.48	1.44	0.97–2.14
Depression					
No	5,015 (96.35%)	Reference		Reference	
Yes	190 (3.65%)	1.38	0.90–2.13	1.21	0.75–1.93
Mitral insufficiency/regurgitation					
No	5,030 (96.64%)	Reference		Reference	
Yes	175 (3.36%)	3.32	2.34–4.72	2.58	1.74–3.81

For peer review only - http://bmjopen.bmj.com/site/about/guidelines.xhtml

Peripheral artery disease					
No	5,086 (97.71%)	Reference		Reference	
Yes	119 (2.29%)	2.47	1.57–3.87	1.60	0.97–2.65

ACEI, angiotensin-converting enzyme inhibitor; ARB, angiotensin receptor blocker; CI, confidence interval; COPD, chronic obstructive pulmonary disease; HF, heart failure; HFpEF, heart failure with preserved ejection fraction; HFrEF, heart failure with reduced ejection fraction; LVEF, left ventricular ejection fraction; MI, myocardial infarction; NT-proBNP, N-terminal pro-B-type natriuretic peptide; OR, odds ratio.

For peer review only

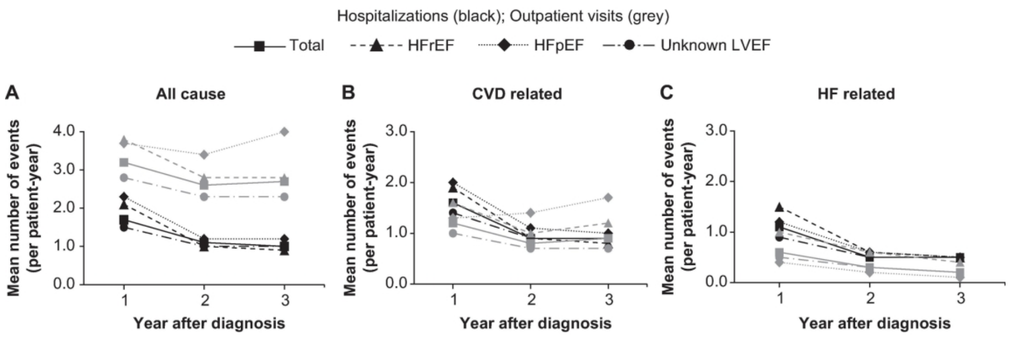

Figure 1: Mean number of hospitalizations (lines in black) and outpatient visits (lines in grey) for incident patients with HF during 2010–2012 by year after HF diagnosis. CVD, cardiovascular disease; HF, heart failure; HFpEF, HF with preserved ejection fraction; HFrEF, HF with reduced ejection fraction; LVEF, left ventricular ejection fraction. Notes: Number of patients 1, 2, and 3 years after diagnosis: 4,648, 2,769, and 1,309 total patients; 1,106, 667, and 323 patients with HFrEF; 609, 353, and 156 patients with HFpEF; 2,933, 1,749, and 830 with Unknown LVEF, respectively.

177x57mm (300 x 300 DPI)

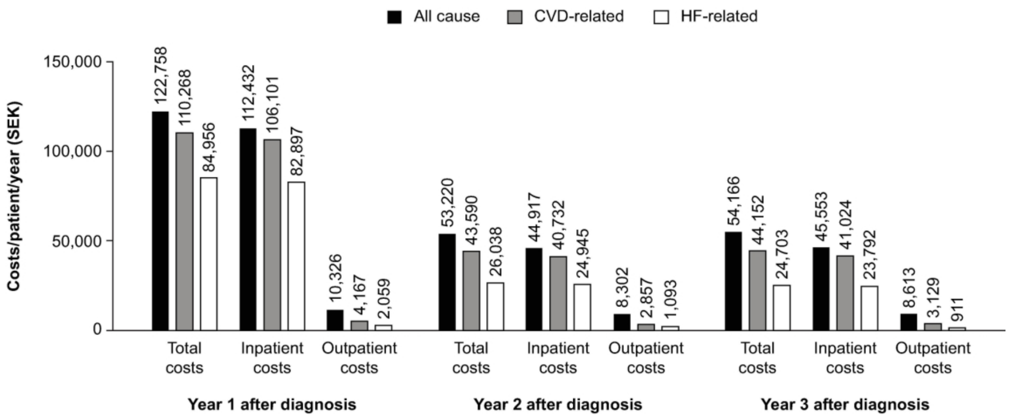

Figure 2: All-cause, CVD-related, and HF-related total, inpatient, and outpatient costs up to 3 years after diagnosis of HF (incident patients with HF, 2010–2012). CVD, cardiovascular disease; HF, heart failure; SEK, Swedish krona.Notes: Number of patients: year 1, n=4,648; year 2, n=2,769; year 3, n=13,095. All costs are given in SEK 2015 values (1 SEK = €0.105).

172x70mm (300 x 300 DPI)

Supporting information

Secondary healthcare costs: pharmacotherapy (the Uppsala County cohort)

In the cohort of patients from the Uppsala County with incident patients with heart failure (HF) during the period of 2010–2012 (N=3,452), the overall drug use (HF-related drugs and other pharmacotherapies) in the year after diagnosis was associated with a mean cost of Swedish krona (SEK) 10,727 (equivalent to €1,126) per patient per year. In general, these costs remained stable for up to 4 years after the diagnosis of HF. However, at year 5, an increase was observed in the mean cost to SEK 12,760 (€1,340) per patient per year. The costs of HF-specific therapy (drugs listed in Table S2) constituted 13% of the total drug costs in the first year after HF diagnosis (mean costs per patient per year at years 1 and 5 after HF diagnosis were SEK 1,395 [€146] and 1,404 [€147], respectively).

For peer review only - http://bmjopen.bmj.com/site/about/guidelines.xhtml

Table S1 List of ICD-10 codes

Comorbidity/risk factor	ICD-10 code
Hypertension	I10–I13, I15
Atrial fibrillation	I48
Angina or MI	I20–I22
Diabetes	E10–E14
Cancer	C00–C97
Cerebrovascular disease	I60–I69
Dyslipidaemia	E78
Anaemia	D50–D64
COPD	J40–J44, J47
Aortic insufficiency/regurgitation	I06, I35, Q231
Dementia	F00–F07
Chronic kidney disease	N18
Depression	F32–F33
Mitral insufficiency/regurgitation	I051, I340, Q233
Peripheral artery disease	I739
**Definitions for calculation of CCI**	
MI	I21–I22, I252
Congestive heart failure	I43, I50, I099, I110, I130, I132, I255, I420, I425–I429, P290
Peripheral vascular disease	I70–I71, I731, I738–I739, I771, I790, I792, K551, K558–K559, Z958–Z959

For peer review only - http://bmjopen.bmj.com/site/about/guidelines.xhtml

Cerebrovascular disease	G45–G46, I60–I69, H340
Dementia	F00–F03, G30, F051, G311
Chronic pulmonary disease	J40–J47, J60–J67, I278–I279, J684, J701, J703
Connective tissue disease-rheumatic disease	M05–M06, M315, M32–M34, M351, M353, M360
Peptic ulcer disease	K25–K28
Mild liver disease	B18, K73–K74, K700–K703, K709, K713–K715, K717, K760, K762–K764, K768–K769, Z944
Diabetes without complications	E100–E101, E106, E108–E111, E116, E118–E121, E126, E128–E131, E136, E138–E141, E146, E148–E149
Diabetes with complications	E102–E105, E107, E112–E115, E117, E122–E125, E127, E132–E135, E137, E142–E145, E147
Paraplegia and hemiplegia	G81–G82, G041, G114, G801–G802, G830–G834, G839
Renal disease	N18–N19, N052–N057, N250, I120, I131, N032–N037, Z490–Z492, Z940, Z992
Cancer	C00–C76, C81–C97
Moderate to severe liver disease	K704, K711, K721, K729, K765–K767, I850, I859, I864, I982
Metastatic carcinoma	C77–C80
AIDS/HIV	B20–B22, B24

AIDS/HIV, human immunodeficiency virus/acquired immunodeficiency syndrome; CCI, Charlson comorbidity index; COPD, chronic obstructive pulmonary disease; ICD-10, International Classification of Diseases and Related Health Problems, Tenth Revision; MI, myocardial infarction.

For peer review only - http://bmjopen.bmj.com/site/about/guidelines.xhtml

Table S2 Heart failure-related drugs (according to the European Society of Cardiology 2012 guidelines[9])

**Drug/drug class**	**Anatomical Therapeutic Chemical code**
Angiotensin-converting enzyme inhibitor	C09A, C09B
Angiotensin receptor blocker	C09C, C09D
Beta blocker	C07
Mineralocorticoid receptor antagonist	C03DA01-C03DA04
Ivabradine (sinus node I_f channel inhibitor)	C01EB17
Diuretics	C03
Digoxin	C01AA05
Nitrates and nitrites	C01DA

For peer review only - http://bmjopen.bmj.com/site/about/guidelines.xhtml

Table S3 Sub-distribution hazard model of the proportion of incident patients with HF from the Uppsala and Västerbotten counties with a second HF diagnosis in 2010–2012 who had all-cause hospitalizations within 3 years and cumulative incidence function estimates for the proportion of hospitalized patients within 0.5, 1, and 3 years after index date

**Characteristic**	**N=4,648**	**Cumulative incidence function of all-cause hospitalizations, % (95% CI)**			**Sub-distribution HR (95% CI)**	***P* value**
		**0.5 years**	**1 year**	**3 years**		
Age group, years						
18–54 (reference)	275	51.6 (45.6–57.4)	58.2 (52.1–63.8)	69.5 (63.0–75.0)	1	
55–64	457	50.3 (45.6–54.8)	60.4 (55.7–64.7)	75.2 (70.1–79.5)	1.07 (0.88–1.29)	0.4983
65–74	1,051	50.5 (47.5–53.5)	60.7 (57.7–63.6)	75.8 (72.7–78.6)	1.11 (0.93–1.31)	0.2417
75–84	1,592	46.2 (43.8–48.7)	57.4 (54.9–59.8)	77.3 (74.7–79.8)	1.06 (0.90–1.25)	0.5110
≥85	1,273	43.4 (40.6–46.1)	53.7 (51.0–56.4)	72.4 (69.5–75.0)	0.97 (0.82–1.16)	0.7664
Sex						
Female (reference)	2,143	46.3 (44.2–48.4)	55.6 (53.5–57.7)	74.5 (72.2–76.6)	1	
Male	2,505	47.8 (45.9–49.8)	59.1 (57.1–61.0)	75.5 (73.4–77.4)	1.04 (0.97–1.12)	0.2858

For peer review only - http://bmjopen.bmj.com/site/about/guidelines.xhtml

Diagnosis setting						
Primary care (reference)	1,118	43.7 (40.8–46.6)	56.1 (53.1–58.9)	79.9 (76.7–82.8)	1	
Secondary care	3,528	48.2 (46.5–49.8)	57.9 (56.3–59.5)	73.6 (71.8–75.2)	0.93 (0.86–1.01)	0.0675
Unknown	2	N/A	N/A	N/A	1.80 (1.15–2.82)	0.0098
HF phenotype						
HFrEF (reference)	609	56.5 (52.4–60.3)	64.7 (60.8–68.4)	80.5 (76.5–83.9)	1	
HFpEF	1,106	54.9 (51.9–57.8)	63.7 (60.8–66.5)	76.4 (73.4–79.0)	0.91 (0.81–1.03)	0.1404
Unknown LVEF	2,933	42.3 (40.5–44.1)	53.6 (51.8–55.4)	73.6 (71.6–75.4)	0.77 (0.70–0.86)	<0.0001
NT-proBNP, pg/mL						
0–1,000 (reference)	912	43.0 (39.7–46.2)	55.2 (51.9–58.3)	76.4 (73.0–79.6)	1	
1,001–3,000	1,283	47.3 (44.6–50.0)	58.6 (55.9–61.3)	78.0 (75.2–80.6)	1.06 (0.97–1.17)	0.2095
>3,000	1,601	54.5 (52.0–56.9)	63.7 (61.3–66.0)	76.4 (73.9–78.7)	1.19 (1.07–1.31)	0.0007
Missing	852	37.6 (34.3–40.8)	46.6 (43.2–49.9)	66.2 (62.5–69.7)	0.84 (0.75–0.94)	0.0028

For peer review only

For peer review only - http://bmjopen.bmj.com/site/about/guidelines.xhtml

CI, confidence interval; HF, heart failure; HFpEF, HF with preserved ejection fraction; HFrEF, HF with reduced ejection fraction; HR, hazard ratio; LVEF, left ventricular ejection fraction; NT-proBNP, N-terminal pro-B-type natriuretic peptide.

Note: Multivariate sub-distribution hazard model with death as competing risk used to obtain the sub-distribution HRs. Cumulative incidence function was used to estimate percentage of patients with all-cause hospitalizations in 0.5, 1, and 3 years after index date.

For peer review only

For peer review only - http://bmjopen.bmj.com/site/about/guidelines.xhtml

Table S4 Costs associated with primary care contacts for incident patients with HF between 2010 and 2012 in the Uppsala County

	Mean (SD) costs per patient per year in SEK				
	**Year 1 (N=3,453)**	**Year 2 (N=2,705)**	**Year 3 (N=1,846)**	**Year 4 (N=891)**	**Year 5 (N=166)**
Total costs	10,347 (12,685)	9,521 (11,974)	9,490 (12,225)	9,754 (12,755)	10,869 (16,857)
Family physician visits	5,432 (8,634)	4,718 (7,723)	4,668 (7,652)	4,641 (7,693)	5,153 (7,714)
Nurse visits	4,050 (7,251)	4,068 (7,475)	4,100 (7,546)	4,388 (8,107)	4,992 (11,685)
Blood tests	866 (1,254)	736 (1,143)	722 (1,133)	726 (1,128)	724 (1,034)

HF, heart failure; SD, standard deviation; SEK, Swedish krona.

Notes: Primary care costs (available only for the Uppsala County cohort) per visit or per test were calculated based on published price lists. All costs are in SEK 2015 values (1 SEK = €0.105).

For peer review only

For peer review only - http://bmjopen.bmj.com/site/about/guidelines.xhtml

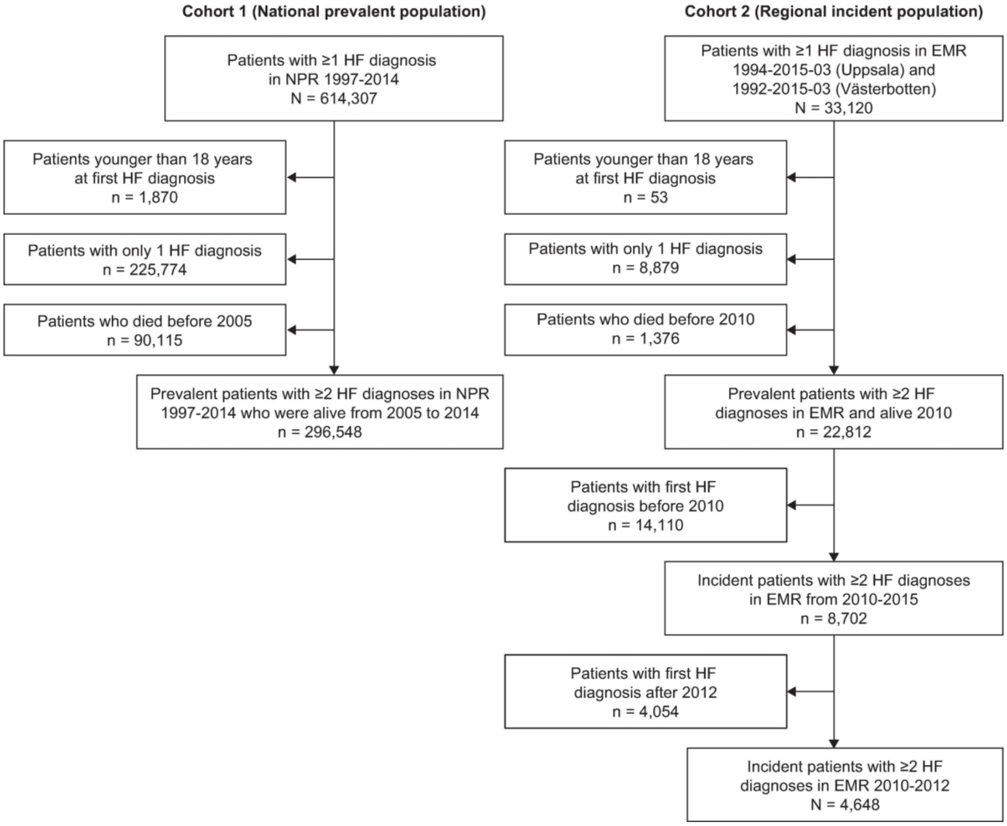

Figure S1: Patient flow. EMR, electronic medical record; HF, heart failure; HFpEF, HF with preserved ejection fraction; HFrEF, HF with reduced ejection fraction; LVEF, left ventricular ejection fraction; NPR, National Patient Register. %"Notes: In cohort 1, data from 614,307 patients were extracted from the Swedish NPR on the basis of an observed HF diagnosis between 1997 and 2014; of which, data from 296,548 patients met the inclusion criteria of at least two HF diagnoses in the analysis period (2005–2014) with no HF diagnosis in the look-back period (1997–2004). In cohort 2, data from 33,120 patients were extracted from the EMRs on the basis of an observed HF diagnosis between 1994 and 2015 (Uppsala County) and between 1992 and 2015 (Västerbotten County); of which, data from 4,648 patients met the inclusion criteria of at least two HF diagnoses in the analysis period (2010–2012) and no HF diagnosis in the look-back period (NPR: 1997–2009, EMR: 1992–2009). A total of 5,205 analyzable patients with 3-year follow-up data were identified; of the 1,819 patients for whom LVEF data were available, 64% had HFrEF and 36% had HFpEF. The incident HF population comprised those with no HF diagnosis in the look-back periods.

177x145mm (300 x 300 DPI)

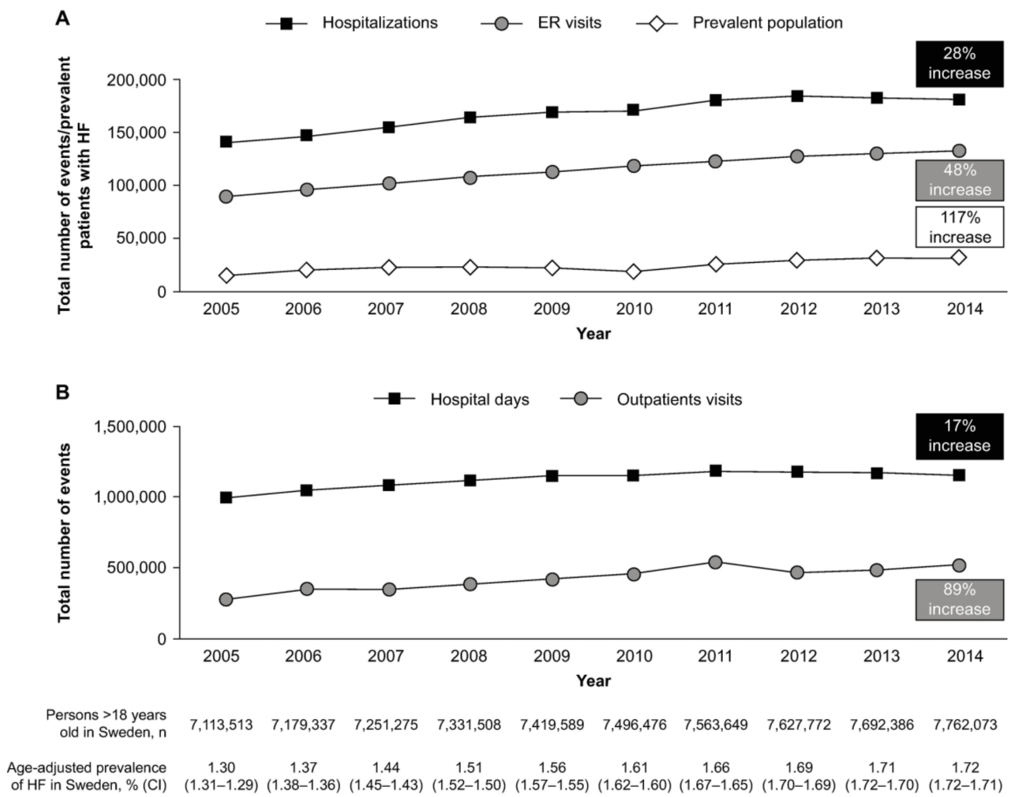

Figure S2: Total number of (a) hospitalizations and ER visits and (b) hospital days and outpatient visits in relation to the absolute number of prevalent adult patients with HF extracted from the NPR, size of adult population in Sweden, and age-adjusted prevalence of HF in Sweden from 2005 to 2014. CI, confidence interval; ER, emergency room; HF, heart failure; NPR, National Patient Register.

171x134mm (300 x 300 DPI)

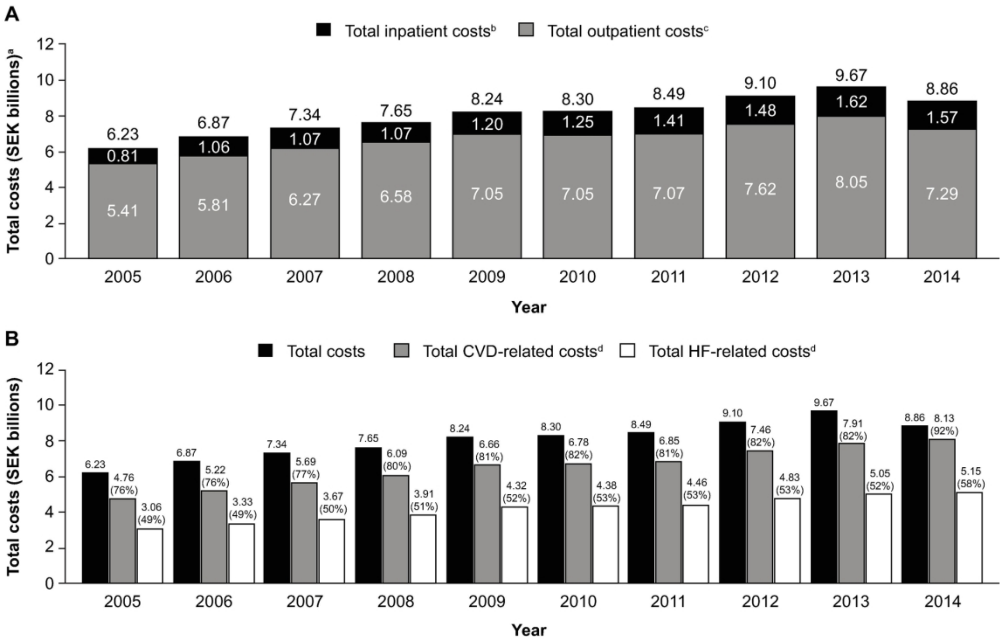

Figure S3: Costs associated with secondary care of prevalent patients with HF from 2005 to 2014: (a) total, inpatient, and outpatient costs and (b) total, CVD-related, and HF-related costs. CVD, cardiovascular disease; ER, emergency room; HF, heart failure; SEK, Swedish krona. Notes: aNumbers above bars indicate total costs (sum of inpatient and outpatient costs). bTotal inpatient costs correspond to the costs associated with hospitalizations. cTotal outpatient costs are the sum of the costs associated with outpatient and ER visits. dCVD-related and HF-related costs are not mutually exclusive; percentages shown in brackets represent the proportion of CVD-related and HF-related costs from the total costs.

169x108mm (300 x 300 DPI)

STROBE 2007 (v4) Statement—Checklist of items that should be included in reports of *cohort studies*

Section/Topic	Item #	Recommendation	Reported on page #
**Title and abstract**	1	(*a*) Indicate the study's design with a commonly used term in the title or the abstract	1
		(*b*) Provide in the abstract an informative and balanced summary of what was done and what was found	2
**Introduction**			
Background/rationale	2	Explain the scientific background and rationale for the investigation being reported	4
Objectives	3	State specific objectives, including any prespecified hypotheses	4
**Methods**			
Study design	4	Present key elements of study design early in the paper	4-5
Setting	5	Describe the setting, locations, and relevant dates, including periods of recruitment, exposure, follow-up, and data collection	4-5
Participants	6	(*a*) Give the eligibility criteria, and the sources and methods of selection of participants. Describe methods of follow-up	5
		(*b*) For matched studies, give matching criteria and number of exposed and unexposed	NA
Variables	7	Clearly define all outcomes, exposures, predictors, potential confounders, and effect modifiers. Give diagnostic criteria, if applicable	7-8
Data sources/ measurement	8*	For each variable of interest, give sources of data and details of methods of assessment (measurement). Describe comparability of assessment methods if there is more than one group	7-8
Bias	9	Describe any efforts to address potential sources of bias	6
Study size	10	Explain how the study size was arrived at	NA
Quantitative variables	11	Explain how quantitative variables were handled in the analyses. If applicable, describe which groupings were chosen and why	7-8
Statistical methods	12	(*a*) Describe all statistical methods, including those used to control for confounding	8
		(*b*) Describe any methods used to examine subgroups and interactions	7-8
		(*c*) Explain how missing data were addressed	NA
		(*d*) If applicable, explain how loss to follow-up was addressed	NA
		(*e*) Describe any sensitivity analyses	NA
**Results**			

For peer review only

Participants	13*	(a) Report numbers of individuals at each stage of study—eg numbers potentially eligible, examined for eligibility, confirmed eligible, included in the study, completing follow-up, and analysed	8-9
		(b) Give reasons for non-participation at each stage	NA
		(c) Consider use of a flow diagram	8 (Supplementary document)
Descriptive data	14*	(a) Give characteristics of study participants (eg demographic, clinical, social) and information on exposures and potential confounders	9-10
		(b) Indicate number of participants with missing data for each variable of interest	NA
		(c) Summarise follow-up time (eg, average and total amount)	6
Outcome data	15*	Report numbers of outcome events or summary measures over time	8-12
Main results	16	(*a*) Give unadjusted estimates and, if applicable, confounder-adjusted estimates and their precision (eg, 95% confidence interval). Make clear which confounders were adjusted for and why they were included	8
		(*b*) Report category boundaries when continuous variables were categorized	NA
		(*c*) If relevant, consider translating estimates of relative risk into absolute risk for a meaningful time period	NA
Other analyses	17	Report other analyses done—eg analyses of subgroups and interactions, and sensitivity analyses	9
**Discussion**			
Key results	18	Summarise key results with reference to study objectives	12-15
**Limitations**			
Interpretation	20	Give a cautious overall interpretation of results considering objectives, limitations, multiplicity of analyses, results from similar studies, and other relevant evidence	14-15
Generalisability	21	Discuss the generalisability (external validity) of the study results	12-15
**Other information**			
Funding	22	Give the source of funding and the role of the funders for the present study and, if applicable, for the original study on which the present article is based	15

*Give information separately for cases and controls in case-control studies and, if applicable, for exposed and unexposed groups in cohort and cross-sectional studies.

Note: An Explanation and Elaboration article discusses each checklist item and gives methodological background and published examples of transparent reporting. The STROBE checklist is best used in conjunction with this article (freely available on the Web sites of PLoS Medicine at http://www.plosmedicine.org/, Annals of Internal Medicine at http://www.annals.org/, and Epidemiology at http://www.epidem.com/). Information on the STROBE Initiative is available at www.strobe-statement.org.

For peer review only

BMJ Open

Healthcare resource utilization and costs associated with a heart failure diagnosis: a retrospective, population-based cohort study in Sweden.

Journal:	*BMJ Open*
Manuscript ID	bmjopen-2021-053806.R1
Article Type:	Original research
Date Submitted by the Author:	28-Jul-2021

Complete List of Authors:	Boman, Kurt; Umea University Department of Public Health and Clinical Medicine, Research Unit, Medicine<br>Lindmark, Krister; Umea University, Dept of public health and clinical medicine and Heart Centre<br>Stålhammar, Jan; Uppsala University, Dept of Public Health and Caring Sciences<br>Olofsson, Mona; Umea University Department of Public Health and Clinical Medicine, Research Unit, Medicine<br>Costa-Scharplatz, Madlaina; Novartis Sweden AB, Medical Affairs/RWE<br>Fonseca, Ana Filipa; Novartis Pharma AG<br>Johansson, Stina; IQVIA<br>Heller, Vincent; IQVIA<br>Törnblom, Michael; IQVIA Solutions Sweden AB, Real-World & Analytics Solutions<br>Wikström, Gerhard; Uppsala University Hospital, Institute of Medical Sciences

**Primary Subject Heading**:	Health economics
Secondary Subject Heading:	Health economics
Keywords:	Heart failure < CARDIOLOGY, Adult cardiology < CARDIOLOGY, HEALTH ECONOMICS

SCHOLARONE™ Manuscripts

I, the Submitting Author has the right to grant and does grant on behalf of all authors of the Work (as defined in the below author licence), an exclusive licence and/or a non-exclusive licence for contributions from authors who are: i) UK Crown employees; ii) where BMJ has agreed a CC-BY licence shall apply, and/or iii) in accordance with the terms applicable for US Federal Government officers or employees acting as part of their official duties; on a worldwide, perpetual, irrevocable, royalty-free basis to BMJ Publishing Group Ltd ("BMJ") its licensees and where the relevant Journal is co-owned by BMJ to the co-owners of the Journal, to publish the Work in this journal and any other BMJ products and to exploit all rights, as set out in our licence.

The Submitting Author accepts and understands that any supply made under these terms is made by BMJ to the Submitting Author unless you are acting as an employee on behalf of your employer or a postgraduate student of an affiliated institution which is paying any applicable article publishing charge ("APC") for Open Access articles. Where the Submitting Author wishes to make the Work available on an Open Access basis (and intends to pay the relevant APC), the terms of reuse of such Open Access shall be governed by a Creative Commons licence – details of these licences and which Creative Commons licence will apply to this Work are set out in our licence referred to above.

Other than as permitted in any relevant BMJ Author's Self Archiving Policies, I confirm this Work has not been accepted for publication elsewhere, is not being considered for publication elsewhere and does not duplicate material already published. I confirm all authors consent to publication of this Work and authorise the granting of this licence.

For peer review only

Healthcare resource utilization and costs associated with a heart failure diagnosis: a retrospective, population-based cohort study in Sweden.

Kurt Boman,[1] Krister Lindmark,[2] Jan Stålhammar,[3] Mona Olofsson,[1] Madlaina Costa-Scharplatz,[4] Ana Filipa Fonseca,[5] Stina Johansson,[6] Vincent Heller,[6] Michael Törnblom,[6] Gerhard Wikström[7]

[1]Research Unit, Medicine-Geriatric, Skellefteå County Hospital, Department of Public Health and Clinical Medicine, Umeå University, Umeå, Sweden; [2]Department of Public Health and Clinical Medicine and Heart Centre, Umeå University Hospital, Umeå, Sweden; [3]Department of Public Health and Caring Sciences, Uppsala University, Uppsala, Sweden; [4]Medical Affairs/RWE, Novartis Sweden AB, Stockholm, Sweden; [5]Novartis Pharma AG, Basel, Switzerland; [6] Real-World & Analytics Solutions, IQVIA, Solna, Sweden; [7]Institute for Medical Sciences, Uppsala University, Uppsala, Sweden

Corresponding author

Kurt Boman

Department of Public health and Clinical Medicine,

Umeå University, SE-901 87

Umea, Sweden

Sweden

Tel: +46910771113

Fax: +4691010274

Email: Kurt.Boman@regionvasterbotten.se

For peer review only

Abstract

Objectives: To examine healthcare resource use (HRU) and costs among heart failure (HF) patients using population data from Sweden.

Design: Retrospective, non-interventional cohort study

Setting: Two cohorts were identified from linked national health registers (cohort 1, 2005–2014) and electronic medical records (cohort 2, 2010–2012; primary/secondary care patients from Uppsala and Västerbotten).

Participants Patients (aged ≥18 years) with primary or secondary diagnoses of HF (≥2 ICD-10 classification) during the identification period of January 2005 to March 2015 were included.

Outcome measures: HRU across the HF phenotypes was assessed with logistic regression. Costs were estimated based on diagnosis-related group codes and general price lists.

Results: Total annual costs of secondary care of prevalent HF increased from SEK 6.23 (€0.60) to 8.86 (€0.85) billion between 2005 and 2014. Of 4,648 incident patients, HF phenotype was known for 1,715: reduced ejection fraction [HFrEF]: 64.5%, preserved ejection fraction [HFpEF]: 35.5%. Within 1 year of HF diagnosis, the proportion of patients hospitalized was only marginally higher for HFrEF vs HFpEF (all-cause [95%CI]: 64.7% [60.8–68.4] vs 63.7% [60.8–66.5], hazard ratio [HR] 0.91, P=0.14; cardiovascular disease related [95%CI]: 61.1% [57.1–64.8] vs 60.9% [58.0–63.7], HR 0.93, P=0.28). Frequency of hospitalizations and outpatient visits per patient declined after the first year. All-cause secondary care costs in the first year were SEK 122,758 (€12,890)/patient/year, with HF-specific care accounting for 69% of the costs. Overall, 10% of the most expensive population (younger; predominantly male; more likely to have comorbidities) incurred ~40% of total secondary care costs.

For peer review only

Conclusions: HF-associated costs and HRU are high, especially during the first year of diagnosis. This is driven by high hospitalizations rates. Understanding the profile of resource-intensive patients being at younger age, male sex, and high Charlson comorbidity index (CCI) scores at the time of the HF diagnosis is most likely a sign of more severe disease.

Key words: Heart failure, resource use, cost, HF phenotypes, real-world

Strengths and limitations of this study

- The study provides comprehensive information from two county regions in Sweden on the increase in number of newly diagnosed HF patients, resource use across the HF phenotypes and total annual costs of secondary care of prevalent HF in Sweden.
- The study provides new insights about the profile of resource-intensive patients at the time of the HF diagnosis, which can support guidance in resource allocation and improve patient management.
- A retrospective identification of patients with confirmed HF despite the requirement for at least two HF diagnoses is challenging.
- Primary and secondary care resource use and costs were available only for the Uppsala Region cohort, which limited the direct comparison with the data from the Västerbotten Region where only secondary care resources were available.
- Our data reflect the real-world costs associated with a diagnosis of HF, despite an acknowledged limitation in the true validation of the diagnosis due to lack of echocardiogram records for the majority of patients.

Introduction

For peer review only

Heart failure (HF) represents a major public health challenge in developed countries and is associated with high healthcare resource consumption[1] with cardiovascular diseases (CVD) as one of the leading cause of mortality in Sweden.[2] HF places a considerable burden on the healthcare systems,[3, 4] and approximately 20% of patients discharged from hospitals after an HF admission were readmitted within 30 days between 2010 and 2013.[5] Inpatient HF care accounts for the largest proportion of healthcare costs,[6] and that the total annual direct cost related to HF is Swedish krona 2.6 billion, corresponding to approximately 2% of the total Swedish healthcare budget.[7]

An ageing population, increasing number of comorbidities, and changed treatment patterns necessitate the examination of healthcare resource use and costs. Therefore, our primary aim was to investigate trends in total healthcare costs from 2005 to 2014 among prevalent and incident patients with HF[8]. Patient characteristics in the context of resource use were analyzed to determine which individuals incurred the greatest resource and secondary care costs. With the growing importance of HF with preserved ejection fraction (HFpEF), this investigation goes a step further than other Swedish studies[3, 4] to present new data comparing resource use and costs among patients according to HF phenotype.

Methods

Study design

This was a retrospective, non-interventional cohort study using longitudinal patient-level data for patients with HF in Sweden.[9] Data were collected from electronic medical records (EMRs) from primary and secondary care and from local echocardiography registries in the counties of Uppsala (two hospitals and 46 primary care centres [PCCs]) and Västerbotten (three hospitals and 37 PCCs). EMR data were linked via unique identifiers issued by the National Board of Health and Welfare (NBHW) to data from national health registers, including the National Patient Register (NPR). Data were pseudonymized before the linked

For peer review only

database was released to the research group, and ethical approval was obtained from the regional Ethical Review Board in Uppsala, Sweden (2015-045) before data extraction. No informed consent was required for this retrospective, pseudonymized study.

Patients

Patients (aged ≥18 years) with primary or secondary diagnoses of HF during the identification period of January 2005 to March 2015 were included. Analyzing data from patients treated in both primary and secondary care by linking NPR data with EMR data presents the challenge of distinct HF diagnosis specificity across the two settings. Two HF diagnoses were required to reduce the uncertainty surrounding HF diagnosis in primary care and increase overall diagnosis specificity. Diagnosis of HF was based on the International Classification of Diseases and Related Health Problems, tenth revision (ICD-10) diagnostic codes of I50, I42.0, I42.6, I42.7, I42.9, I11.0, I13.0, or I13.2 for the primary or secondary diagnoses (Table S1).

The national cohort comprised prevalent patients with HF registered in the NPR from secondary care between January 2005 and December 2014, while the regional cohort (Uppsala and Västerbotten) included incident patients with HF diagnoses registered in EMRs from either primary or secondary care between January 2010 and March 2015.

The Pygargus Customized eXtraction Program (CXP 3.0) extracted data from EMRs from Uppsala and Västerbotten and subsequently linked to data from the national health registers including the NPR.[10] HF with preserved ejection fraction [HFpEF] and HF with reduced ejection fraction [HFrEF]) was determined based only on data from local echocardiography registries, and for cohort 2, HFpEF was defined as a left ventricular ejection fraction (LVEF) of ≥50% and HFrEF was defined as an LVEF of <50%. This cut-off is based on data constraints; LVEF is recorded categorically in the echocardiography

For peer review only

registers, with the categories used differing between the county councils such that 50% was the only option that allowed for a common threshold.

Data extraction and study timelines

Data were extracted from EMRs based on an observed HF diagnosis during 1994-2015 for the Uppsala County and during 1992–2016 for the Västerbotten County. The analysis period was adjusted to 1 January 2010 owing to inconsistent patterns of HF registration in the Uppsala County EMR systems. The look-back period in the EMR data was from the time the first data were available until 31 December 2009. For NPR data, the look-back period was from 1 January 1997 for inpatient care and 1 January 2001 for outpatient care to 31 December 2004 (Figure S1) to identify prevalent patients with HF at a national level. This prevalent patient cohort was used to assess trends in total healthcare resource use and costs from 2005 to 2014.

Newly diagnosed individuals from the Uppsala County and during the analysis period made up the incident HF population. To enable all patients to have at least 3 years of potential follow-up, all analyses were performed on patients diagnosed with incident HF between 2010 and 2012 (Figure S1).

For the analyses of clinical characteristics, healthcare resource use, and costs, the index date was defined as the date of first HF diagnosis. However, for the analyses of hospitalizations, the index date was defined as a patient's second HF diagnosis to improve the accuracy of diagnosis and minimize the effects of immortal time bias. Follow-up was defined as the period between the second HF diagnosis and the end of the study, end of EMR collection for those patients who moved to another region, or date of death, whichever came first.

For peer review only

Variables analyzed and statistical analyses

For categorical outcomes a χ^2 test was performed; for comparison of means, a *t*-test was performed and confidence intervals (CIs) were calculated at a 95% level. Descriptive statistics assessed baseline clinical characteristics (Table 1) on the date of first HF diagnosis, since this was truly the first time that the patients were considered to have HF. Comorbidities, data from primary (EMR) and secondary (EMR and NPR) care stratified according to HF phenotype, were summarized according to the ICD-10 codes (Table S1). Comorbidities were collected from visits that occurred 0–5 years before the date of the first HF diagnosis. Charlson comorbidity index (CCI) scores during this time, ranging from 0 to greater than 10 (higher scores indicate greater comorbidity), were calculated.

All-cause CVD-related hospitalizations were analyzed using a subdistribution hazard model, proportion of patients having all-cause CVD-related hospitalizations occurring within 0.5, 1 and 3 years after the second HF diagnosis were estimated by a cumulative incidence function.. The time to all-cause and time to CVD-related hospitalizations/readmissions were estimated from the date of the second HF diagnosis using a Cox proportional hazards model, with age group, sex, diagnosis setting (primary care, secondary care, unknown), HF phenotype, and N-terminal pro B-type natriuretic peptide (NT-proBNP) level (0–1,000, 1,001–3,000, and >3,000 pg/mL) as covariates. Data for the mean and median (interquartile range [IQR]) number of hospitalizations and outpatient visits for all-cause, CVD-related, and HF-related events, as well as mean length of stay (LOS), were analyzed by HF phenotype and year since HF diagnosis.

Costs were estimated by year since the first HF diagnosis. All costs are presented as SEK 2015 values (based on the Consumer Price Index,[11] with the equivalent value in euros [€], based on the historical exchange rate in January 2015 (1 SEK = €0.105). Analyses of all-cause, CVD-related, and HF-related costs associated with secondary care were performed

For peer review only

based on diagnosis-related group codes and price lists, as determined by the NBHW.[12] Costs associated with primary care comprised costs of family physician and nurse visits as well as costs of blood tests. Primary care costs were based on general price lists and were available for the Uppsala County only, as were costs related to pharmacotherapy.[13] The total cost of all drug use for incident patients with HF was estimated, as was the aggregated cost of HF-related drugs, based on the 2012 European Society of Cardiology (ESC) guidelines (Table S2).[14] Logistic regression assessed baseline characteristics of patients who incurred the greatest secondary care costs related to HF, defined as the top 10% most resource-intensive patients.

SAS version 9.3 or higher was used for statistical analysis and data management.

Patient and Public Involvement

No patients or members of the public were involved in this study.

Results

National prevalent population with HF

The absolute number of prevalent patients with HF in Sweden increased by 1.5-fold between 2005 and 2014 from 89,837 to 133,220 (overall population increased from 7,113,513 to 7,762,073), with a corresponding increase in age-adjusted prevalence from 1.30% to 1.72%.

Increase in total costs of prevalent patients with HF over time.

The total number of hospitalizations, hospital days, emergency room (ER) visits, and outpatient visits in secondary care increased from 2005 to 2014 by between 1.2- and 2.2-fold (Figure S2). The number of all-cause hospitalizations increased from 141,941 to 181,374 (difference: 39,433; 28% increase). The number of hospital days increased from 998,512 to 1,165,310 (difference: 166,798; 17% increase), indicating a decreased mean LOS of 4.23

For peer review only

days per hospitalization. ER visits increased from 14,272 to 31,037 (difference: 16,765; 117% increase). Outpatient visits in secondary care increased from 262,771 to 497,230 (difference: 234,459; 89% increase).

The total annual costs associated with the secondary care of prevalent patients with HF increased by 1.4-fold from SEK 6.23 billion (€0.60 billion) in 2005 to SEK 8.86 billion (€0.85 billion) in 2014, mainly driven by a corresponding increase in the overall resource use in inpatient care (52%) (Figure S3a). On an average, inpatient and outpatient costs accounted for 84% and 16% of the total costs, respectively. On an average, 81% of the total costs of secondary care were attributed to CVD-related costs; 64% of these were attributed to HF-related costs (Figure S3b). HF-related costs accounted for 52% of the total costs.

Regional incident population with HF

Data from 104,562 patients were identified from the Uppsala and Västerbotten counties; among these, 8,702 (8.3%) patients had a defined HF diagnosis. Information was available for 4,648 incident patients with HF between 2010 and 2012 (Figure S1). HF phenotype was characterized for 1,715 patients (36.9%); 1,106 (64.5%) patients had HFrEF and 609 (35.5%) had HFpEF (Figure S1).

Patient demographics and clinical characteristics

Table 1 presents the baseline clinical characteristics of patients, overall and by HF phenotype. Mean (median, IQR) age of the patients was 76.8 (79.0, 69.5–86.7) years, more than one-quarter (27.8%) were aged 85 years or more, and 53.8% were male. Most first HF diagnoses occurred in secondary care (75.9%) rather than in primary care (24.1%) (Table 2).

For peer review only

Hospitalizations

All-cause and CVD-related hospitalizations

The cumulative incidence function (95% CI) of the proportion of patients with all-cause hospitalizations within the first year of HF diagnosis was 64.7% (60.8–68.4) for patients with HFrEF and 63.7% (60.8–66.5) for patients with HFpEF; the corresponding sub-distribution hazard ratio (HR) over the follow-up period for HFrEF compared with HFpEF was 0.91 (95% CI: 0.81–1.03; *P*=0.14) (Table S3). The cumulative incidence function of the proportion of patients with all-cause hospitalizations was similar for patients who had their first HF diagnosis in secondary vs primary care; the corresponding sub-distribution HR for the follow-up period for secondary vs primary care was 0.93 (95% CI: 0.86–1.01; *P*<0.07) (Table S3). Higher NT-proBNP levels were associated with a greater risk of all-cause hospitalizations (Table S3), also seen for the proportion of patients with CVD-related hospitalizations by HF phenotype, diagnosis setting, NT-proBNP level, and age (Table 2).

Number of hospitalizations and outpatient visits and LOS

Mean and median number of all-cause, CVD-related, and HF-related hospitalizations and outpatient visits per patient decreased from the first year of HF diagnosis and stabilized between years 2 and 3 (Figure 1). Similar trends in hospitalizations and outpatient visits compared with those observed in the overall population were observed for patients with HFrEF and HFpEF, except that in patients with HFpEF, the mean number of CVD-related outpatient visits increased from the first year after HF diagnosis (Figure 1). The mean (median, IQR) LOS for all-cause, CVD-related, and HF-related hospitalizations was 17.5 (8, 2–23), 16.5 (8, 2–22), and 12.8 (6, 0–16) days in the first year after HF diagnosis, declining at 3 years after diagnosis to 7.6 (0, 0–8), 7.0 (0, 0–7), and 4.2 (0, 0–2) days, respectively.

For peer review only

Patients with HFrEF had a shorter LOS for all-cause, CVD-related, and HF-related events than those with HFpEF (data not shown).

Healthcare costs per patient for the first year after the index date

Secondary healthcare (overall, inpatient, and outpatient) costs

Total and inpatient all-cause costs decreased by more than half after the first year of HF diagnosis (Figure 2). A reduction in all-cause outpatient costs was also noted at the second year after HF diagnosis, relative to the first year. Similar trends were seen for CVD-related and HF-related costs. In the first year after HF diagnosis, total all-cause secondary care costs were SEK 122,758 (€12,890) per patient per year, which declined to SEK 53,220 (€5,588) per patient per year by year 2 after diagnosis. The corresponding total CVD-related and HF-related costs were SEK 110,268 (€11,578) and SEK 84,956 (€8,920) per patient per year in the first year, which decreased to SEK 43,590 (€4,577) and 26,038 (€2,734) per patient per year by year 2, respectively. Therefore, HF-specific inpatient and outpatient costs accounted for 69% of the total costs in the first year after HF diagnosis, declining to 49% during the second year after diagnosis (and 46% during the third year after diagnosis). Inpatient care constituted more than 90% of the total secondary healthcare costs per patient at 1 year after diagnosis for all-cause (SEK 112,432 [€11,805]), CVD-related (SEK 106,101 [€11,141]), and HF-related (SEK 82,897 [€8,704]) events. By comparison, the mean total primary care cost for the Uppsala County cohort only was SEK 10,347 (€1,086) per patient per year (Table S4; Supplementary Information).

Drivers of high resource use and costs

Of the total patient population, 10% (n=465) incurred approximately 40% of the total secondary healthcare costs. Compared with 'other' (non-resource intensive) patients, those in

For peer review only

the resource-intensive population were likely to have a lower mean age, belong to the male sex, have a mean NT-proBNP level above 3,000 pg/mL, and a number of comorbidities; had a known LVEF; and had not received either an angiotensin-converting enzyme inhibitor or angiotensin receptor blocker (Table 3).

Discussion

This study found that (i) the absolute number of patients with HF in Sweden continues to increase; (ii) the total cost of secondary care of prevalent patients with HF increased and was primarily driven by costs associated with inpatient care; (iii) patients with newly diagnosed HF were at a high risk of frequent hospitalizations during the first year after diagnosis, especially in those diagnosed in secondary vs primary care; (iv) HFpEF was associated with greater resource use compared with HFrEF or an unknown LVEF; (v) during the first year, hospitalizations (inpatient care) constituted more than 90% of the total secondary healthcare costs, with 69% of all secondary healthcare costs being attributable to HF-specific care; and (iv) approximately 10% of the patients were highly resource-intensive.

In HF management, hospitalizations are a key challenge because they are highly resource intensive and costly.[3, 4, 15-17] Not only are patients with HF frequently hospitalized, but duration of their admissions are also often lengthy, particularly in Europe compared with the US.[(15, 16)] In the current study, the mean hospital LOS during the first year after HF diagnosis was more than 16 days for patients admitted for all-cause or CVD-related events and 12.8 days for patients admitted for HF-related events.

The clinical implication of our data is to identify factors responsible for the high costs of HF within the first year of diagnosis. Such factors could include patients being discharged from hospital too early to ensure full evaluation, patients not being initiated on evidence-based

For peer review only

therapy or devices, delayed or incomplete follow-up in patient management programmes, and loss of continuity and/or ineffective HF follow-ups.

An important observation concerned the risk of hospitalization/readmission over the follow-up period, which was significantly greater for patients receiving their first HF diagnosis in secondary vs primary care. This might be the result of patients presenting with more severe HF and/or more severe chronic HF and patients with a diagnosis of acute HF. Accordingly, the total costs associated with primary care, available for the Uppsala County only, were SEK 10,347 (€1,086) per patient and were much lower than the secondary care costs in the first year after diagnosis. These costs generally remained stable over the first 4 years of follow-up and increased slightly at year 5, which could be a result of the increasing age of the population.

Patients with HFpEF were also at a higher risk of all-cause and CVD-related hospitalizations and required more outpatient visits at 3 years than those with HFrEF, potentially because patients with HFpEF tended to be older and/or have a higher comorbidity burden. Importantly, a higher proportion of deaths in patients with HFpEF than in those with HFrEF may have a non-CVD cause.[18, 19] Mortality data presented in Lindmark et al[(20)] show that the HR for the 1-year all-cause mortality rate was significantly lower for HFrEF than for HFpEF (0.77, 95% CI 0.62–0.96, *P*=0.0159).

We found a decline in resource use observed after the first year of diagnosis and substantial reductions in healthcare costs, with total all-cause costs per patient decreasing by more than half in the second year. This decrease reflects patient-level, longitudinal data trends and, in fact, from a total population/healthcare system perspective, the opposite was true with an increase in costs over time because of an increasing prevalence of HF and a shift in patient profile towards older and sicker patients.[20] In our incident population, the high financial costs during the first year after diagnosis may be partly explained by survivor bias,

For peer review only

assuming that patients alive in later years have less severe HF symptoms and require less cost-intensive treatment, such as the need for re-hospitalizations and longer LOS. Other possible explanations include the use of both more intensive investigations to define the aetiology of HF during the first year and expensive medicines, devices, and/or surgical treatments during the initial phase of treatment. The costs reported herein are generally similar to those reported in another Swedish EMR- and register-based study that analyzed data from 2006.[4]

Similar to other economic studies in HF,[3, 21, 22] the inpatient care accounted for most of the total secondary care costs (~90%) and showed the greatest proportional decline in costs from the peak at 1 year after diagnosis, consistent with the reduction seen in the number of hospitalizations. Similar trends were observed for CVD-related and HF-related costs per patient. Data for the 10% of patients who accumulated the highest healthcare costs showed that patients with several different comorbidities were associated with a higher resource use. The most resource-intensive patients tended to be male with high NT-proBNP levels. Patients with high/increasing biomarker levels tend to be followed up closely by specialists because of the potential for adverse outcomes.[23]

A limitation of the current study was the challenge of retrospectively identifying patients with confirmed HF. The use of two, rather than one, HF diagnoses was expected to increase the certainty surrounding the HF diagnosis, although it could potentially have led to exclusion of more recent or mild cases of HF. Second limitation concerned the definition of subgroups based on the HF phenotype. The limited number of echocardiographs required for the verification of the HF diagnosis was a major drawback, meaning that a large proportion of patients had missing information for both LVEF and structural and functional pathologies. This may have introduced some level of bias because it could be assumed that the echocardiography was performed only in more severe cases of HF, meaning that the two HF

For peer review only

phenotype subgroups were not wholly representative of the total cohort. Nevertheless, our data reflect the real-world costs associated with a diagnosis of HF, irrespective of the true validation of the diagnosis. Moreover, the threshold for defining HFrEF was an LVEF of ≤50%, which is higher than the recommendations from the ESC of <40%[24]; therefore, a larger number of patients would have been categorized as having HFrEF. Finally, there were missing data on doctor visits for the Västerbotten County cohort because of technical issues relating to the Västerbotten EMR database; thus, primary care resource use and costs were available only for the Uppsala County cohort.

Conclusions

Care for patients with HF represents an increasing financial burden on the Swedish healthcare system. Hospitalizations/readmissions represent a key resource and cost burden regardless of HF phenotype, particularly during the first year after diagnosis, with total costs of HF attributed primarily to those associated with inpatient care. Furthermore, characteristics of patients incurring the greatest healthcare costs included younger age, male sex, and high CCI scores and NT-proBNP levels at the time of the HF diagnosis. Understanding the profile of patients with HF who are likely to incur the greatest healthcare costs is invaluable to guide resource allocation.

Acknowledgements

The authors thank Japinder Kaur of Novartis and Sharon Smalley and Carly Sellick of Pharma Genesis, London, for providing medical writing assistance with this manuscript.

Funding This research was funded by Novartis Pharma AG, Basel, Switzerland.

Competing interest K. Boman, M. Olofsson, and J. Stålhammar received reimbursement from Novartis via IQVIA for performing the study. K. Boman and M. Olofsson have also

For peer review only

received lecture grants from Novartis. K. Lindmark received lecture grants and consultant fees from Novartis. G. Wikström has no conflicts of interest to declare; however, Uppsala University received research funding from Novartis for conducting this study. M. Costa-Scharplatz is an employee of Novartis Sweden AB, Sweden, and A.F. Fonseca is an employee of Novartis Pharma AG, Basel, Switzerland. Data extraction and analysis were conducted by S. Johansson, V. Heller, and M. Törnblom are employees of IQVIA, Sweden, which was commissioned to conduct the study on behalf of Novartis Pharma AG, Basel, Switzerland, and have ongoing consulting and research relationships with Novartis Pharma AG.

Data availability statement Major findings from the study will be published in scientific manuscripts only. The data will not be made available in any other format in order to preserve the privacy of the patients in compliance with local laws and regulation.

Authors contributions

Kurt Boman and Gerhard Wikström were involved in the study and manuscript conceptualization, interpretation of results and development of the first draft of the manuscript. Krister Lindmark contributed to protocol development, study and manuscript conceptualization, results interpretation, refinement of analysis requirements and writing of the manuscript. Stina Johansson, Vincent Heller, Michael Törnblom performed statistical analyses. Jan Stålhammar and Mona Olofsson contributed to protocol development, study conceptualization, interpretation of results and supported the development of the manuscript. Madlaina Costa-Scharplatz and Ana Filipa Fonseca contributed to manuscript conceptualization, interpretation of results, refinement of analysis requirements and supported the development of the manuscript. All authors reviewed the manuscript and approved the final draft of the manuscript for submission.

For peer review only

Figures caption

Figure 1 Mean number of hospitalizations (lines in black) and outpatient visits (lines in grey) for incident patients with HF during 2010–2012 by year after HF diagnosis.

CVD, cardiovascular disease; HF, heart failure; HFpEF, HF with preserved ejection fraction; HFrEF, HF with reduced ejection fraction; LVEF, left ventricular ejection fraction.

Notes: Number of patients 1, 2, and 3 years after diagnosis: 4,648, 2,769, and 1,309 total patients; 1,106, 667, and 323 patients with HFrEF; 609, 353, and 156 patients with HFpEF; 2,933, 1,749, and 830 with Unknown LVEF, respectively.

Figure 2 All-cause, CVD-related, and HF-related total, inpatient, and outpatient costs up to 3 years after diagnosis of HF (incident patients with HF, 2010–2012).

CVD, cardiovascular disease; HF, heart failure; SEK, Swedish krona.

Notes: Number of patients: year 1, n=4,648; year 2, n=2,769; year 3, n=13,095. All costs are given in SEK 2015 values (1 SEK = €0.105).

For peer review only

References

1. Bui AL, Horwich TB, Fonarow GC. Epidemiology and risk profile of heart failure. *Nat Rev Cardiol*. 2011;**8**(1):30-41.

2. Socialstyrelsen (The National Board of Health and Welfare). Statistics on the causes of death 2015. https://www.socialstyrelsen.se/globalassets/sharepoint-dokument/artikelkatalog/statistik/2016-8-3.pdf 2016 [Accessed on 19 December 2017]

3. Stalhammar J, Stern L, Linder R, Sherman S, Parikh R, Ariely R, Deschaseaux C, Wikstrom G. The burden of preserved ejection fraction heart failure in a real-world Swedish patient population. *J Med Econ*. 2014;**17**(1):43-51.

4. Stalhammar J, Stern L, Linder R, Sherman S, Parikh R, Ariely R, Wikstrom G. Resource utilization and cost of heart failure associated with reduced ejection fraction in Swedish patients. *J Med Econ*. 2012;**15**(5):938-46.

5. Sveriges Kommuner och Landsting (SKL) SKoL. Socialstyrelsen. Öppna jämförelser 2014. Hälso-och sjukvård. Jämförelser mellan landsting. Del 2. Indikatorer om sjukdomar och behandlingar. **https://webbutik.skr.se/bilder/artiklar/pdf/7585-157-0.pdf?issuusl=ignore.** 2014 [Accessed on 2 February 2018].

6. Agvall B, Borgquist L, Foldevi M, Dahlstrom U. Cost of heart failure in Swedish primary healthcare. *Scand J Prim Health Care*. 2005;**23**(4):227-32.

7. Ryden-Bergsten T, Andersson F. The health care costs of heart failure in Sweden. *J Intern Med*. 1999;**246**(3):275-84.

8. Boman K LK, Stalhammar J, Olofsson M, Costa-Scharplatz M, Fonseca AF, Bruce Wirta S, Castelo-Branco A, Tornblom M, Wikstrom G. Total annual healthcare costs of heart

For peer review only

failure between 2005 and 2014: a retrospective, population-based study in Sweden. . Poster presented at the European Meeting of the *International Society for Pharmacoeconomics and Outcomes Research* (ISPOR) 2018;10–14 November 2018, Barcelona, Spain.

9. Heidenreich PA AN, Allen LA, Bluemke DA, Butler J, Fonarow GC, Ikonomidis JS, Khavjou O, Konstam MA, Maddox TM, Nichol G, Pham M, Pina IL, Trogdon JG, American Heart Association Advocacy Coordinating C, Council on Arteriosclerosis T, Vascular B, Council on Cardiovascular R, Intervention, Council on Clinical C, Council on E, Prevention, Stroke C. Forecasting the impact of heart failure in the United States: a policy statement from the American Heart Association. *Circ Heart Fail* 2013;**6**:606–19.

10. Martinell M, Jan Stålhammar, J., Hallqvist, J. Automated data extraction—A feasible way to construct patient registers of primary care utilization. *Upsala Journal of Medical Sciences*. 2012;**117**:52-6.

11. Statistics Sweden. Consumer price index [Internet]. **http://www.scb.se/en/finding-statistics/statistics-by-subject-area/prices-and-consumption/consumer-price-index/consumer-price-index-cpi/**. 2016 [Accessed on 12 January 2016].

12. National Board of Health and Welfare. 2015 Vägledning till NordDRG. Swedish version. **https://www.socialstyrelsen.se/globalassets/sharepoint-dokument/artikelkatalog/ovrigt/2015-12-29.pdf**

. 2016 [Accessed 2 February 2018].

13. Tandvårds- och läkemedelsförmånsverket TTSDaPBA. **https://www.tlv.se/in-english/prices-in-our-database.html.** [Accessed 5 March 2018].

14. McMurray JJ, Adamopoulos S, Anker SD, Auricchio A, Bohm M, Dickstein K, Falk V, Filippatos G, Fonseca C, Gomez-Sanchez MA, Jaarsma T, Køber L, Lip GYH, Maggioni AP, Parkhomenko A, Pieske BM, Popescu BA, Rønnevik PK, Rutten FH, Schwitter J, Seferovic P, Stepinska J, Trindade PT, Voors AA, Zannad F, Zeiher A, Bax JJ, Baumgartner

For peer review only

H, Ceconi C, Dean V, Deaton C, Fagard R, Funck-Brentano C, Hasdai D, Hoes A, Kirchhof P, Knuuti J, Kolh P, McDonagh T, Moulin C, Popescu BA, Reiner Z, Sechtem U, Sirnes PA, Tendera M, Torbicki A, Vahanian A, Windecker A, McDonagh T, Sechtem U, Bonet LA, Avraamides P, Ben Lamin HA, Brignole M, Coca A, Cowburn P, Dargie H, Elliott P, Flachskampf FA, Guida GF, Hardman S, Lung B, Merkely B, Mueller C, Nanas JN, Nielsen OW, Ørn S, John T. Parissis JT, Ponikowski P. ESC Guidelines for the diagnosis and treatment of acute and chronic heart failure 2012: The Task Force for the Diagnosis and Treatment of Acute and Chronic Heart Failure 2012 of the European Society of Cardiology. Developed in collaboration with the Heart Failure Association (HFA) of the ESC. *Eur Heart J*. 2012;**33**(14):1787-847.

15. Cowie MR, Anker SD, Cleland JGF, Felker GM, Filippatos G, Jaarsma T, Jourdain P, Knight E, Massie B, Ponikowski P, López-Sendón J. Improving care for patients with acute heart failure: before, during and after hospitalization. *ESC Heart Fail*. 2014;**1**(2):110-45.

16. Braunschweig F, Cowie MR, Auricchio A. What are the costs of heart failure? *Europace*. 2011;**13** Suppl 2:ii13-7.

17. Kilgore M, Patel HK, Kielhorn A, Maya JF, Sharma P. Economic burden of hospitalizations of Medicare beneficiaries with heart failure. *Risk Manag Healthc Policy*. 2017;**10**:63-70.

18. Dunlay SM, Redfield MM, Weston SA, Therneau TM, Hall Long K, Shah ND, Roger VL. Hospitalizations after heart failure diagnosis a community perspective. *J Am Coll Cardiol*. 2009;**54**(18):1695-702.

19. Chan MM, Lam CS. How do patients with heart failure with preserved ejection fraction die? *Eur J Heart Fail*. 2013;**15**(6):604-13.

20. Lindmark K, Boman K, Olofsson M, Tornblom M, Levine A, Castelo-Branco A, Schlienger R, Wirta SB, Stålhammar J, Wikström G. Epidemiology of heart failure and

For peer review only

trends in diagnostic work-up: a retrospective, population-based cohort study in Sweden. *Clin Epidemiol*. 2019;**11**:231-44.

21. Stafylas P, Farmakis D, Kourlaba G, Giamouzis G, Tsarouhas K, Maniadakis N, Parissis J. The heart failure pandemic: The clinical and economic burden in Greece. *Int J Cardiol*. 2017;**227**:923-9.

22. Mejhert M, Lindgren P, Schill O, Edner M, Persson H, Kahan T. Long term health care consumption and cost expenditure in systolic heart failure. *Eur J Intern Med*. 2013;**24**(3):260-5.

23. Kim HN, Januzzi JL, Jr. Natriuretic peptide testing in heart failure. *Circulation*. 2011;**123**(18):2015-9.

24. Ponikowski P, Voors AA, Anker SD, Bueno H, Cleland JGF, Coats AJS, Falk V, González-Juanatey JR, Harjola V, Jankowska EA, Jessup M, Linde C, Nihoyannopoulos P, Parissis JT, Pieske B, Riley JP, Rosano GMC, Ruilope LM, Ruschitzka F, Rutten FH, Meer P. 2016 ESC Guidelines for the diagnosis and treatment of acute and chronic heart failure: The Task Force for the diagnosis and treatment of acute and chronic heart failure of the European Society of Cardiology (ESC)Developed with the special contribution of the Heart Failure Association (HFA) of the ESC. *Eur Heart J*. 2016;**37**(27):2129-200.

For peer review only

Tables

Table 1 Demographic and clinical characteristics at baseline for incident patients with HF from the Uppsala and Västerbotten counties with a first HF diagnosis in 2010–2012, overall and by HF phenotype

Characteristic	Overall population (N=5,205)	HF phenotype HFpEF (n=652)	HFrEF (n=1,167)	Unknown LVEF (n=3,386)
Mean age at index date (SD), years	76.8 (12.3)	74.8 (12.2)	70.3 (13.0)	79.4 (11.1)
Median age at index date (IQR), years	79.0 (69.5, 85.7)	76.8 (68.3, 83.6)	71.2 (63.1, 80.3)	81.5 (73.4, 87.2)
Sex, n (%)				
Women	2,405 (46.2)	341 (52.3)	380 (32.6)	1,684 (49.7)
Men	2,800 (53.8)	311 (47.7)	787 (67.4)	1,702 (50.3)
Mean NT-proBNP level (SD), pg/mL	4,909.1 (8,202.9)	4,270.8 (7,576.7)	6,217.1 (9,495.2)	4,543.4 (7,730.5)
Mean SBP/DBP (SD), mmHg	139.7 (25.6)/ 78.7 (15.3)	141.8 (27.1)/ 76.8 (14.6)	134.9 (24.3)/ 79.7 (15.3)	140.9 (25.6)/ 78.8 (15.3)
Mean eGFR (SD), mL/min/1.73 m^2	51.9 (29.1)	49.6 (27.9)	60.6 (30.2)	44.2 (26.0)
Mean CCI[a,b] (SD)	1.8 (2.2)	1.8 (2.2)	1.7 (2.1)	1.9 (2.2)
Common comorbidities and risk factors, n (%)[b,c]				
Hypertension	2,679 (51.5)	366 (56.1)	476 (40.8)	1,837 (54.3)
Atrial fibrillation	1,593 (30.6)	212 (32.5)	247 (21.2)	1,134 (33.5)
IHD (angina or MI)	1,198 (23.0)	122 (18.7)	303 (26.0)	773 (22.8)
Diabetes	952 (18.3)	137 (21.0)	197 (16.9)	618 (18.3)
Cancer	739 (14.2)	118 (18.1)	141 (12.1)	480 (14.2)
Dyslipidaemia	676 (13.0)	89 (13.7)	152 (13.0)	435 (12.8)
Cerebrovascular disease	660 (12.7)	70 (10.7)	109 (9.3)	481 (14.2)
Anaemia	645 (12.4)	104 (16.0)	101 (8.7)	440 (13.0)

CCI, Charlson comorbidity index; DBP, diastolic blood pressure; eGFR, estimated glomerular filtration rate; HF, heart failure; HFpEF, HF with preserved ejection fraction; HFrEF, HF with reduced ejection fraction; IHD, ischaemic heart disease; IQR, interquartile range; LVEF, left ventricular ejection fraction; MI, myocardial infarction; NT-pro-BNP, N-terminal pro-B-type natriuretic peptide; SBP, systolic blood pressure; SD, standard deviation.

For peer review only

Notes: Data are either number (n) and percentage (%) or mean (SD). [a]Includes patients with a CCI of zero (ie no comorbidities). [b]Comorbidities and underlying cardiac diseases occurring 0–5 years before the index date. [c]Comorbidities and underlying cardiac diseases occurring in ≥10% of the overall population.

For peer review only

Table 2 Sub-distribution hazard model for the proportion of incident patients with HF from the Uppsala and Västerbotten counties with a second HF diagnosis in 2010–2012 who had CVD-related hospitalizations within 3 years and cumulative incidence function estimates for the proportion of patients hospitalized within 0.5, 1, and 3 years after index date

**Characteristic**	**N=4,648**	**Cumulative incidence function of CVD-related hospitalizations, % (95% CI)**			**Sub-distribution HR (95% CI)**	***P* value**
		**0.5 years**	**1 year**	**3 years**		
Age group, years						
18–54 (Reference)	275	46.9 (40.9–52.7)	52.0 (45.9–57.7)	62.5 (55.9–68.3)	1	
55–64	457	47.0 (42.4–51.5)	56.5 (51.8–60.9)	71.8 (66.7–76.3)	1.15 (0.95–1.41)	0.16
65–74	1,051	47.3 (44.2–50.3)	57.5 (54.4–60.4)	72.5 (69.2–75.4)	1.21 (1.01–1.45)	0.0383
75–84	1,592	44.6 (42.1–47.0)	55.3 (52.9–57.7)	75.6 (72.8–78.0)	1.21 (1.02–1.45)	0.0305
≥85	1,273	41.9 (39.2–44.6)	52.2 (49.4–54.9)	70.7 (67.8–73.4)	1.13 (0.94–1.35)	0.19
Sex						
Female (Reference)	2,143	43.8 (41.7–45.9)	52.9 (50.8–55.0)	71.7 (69.4–73.9)	1	
Male	2,505	45.8 (43.8–47.7)	56.5 (54.6–58.4)	73.0 (70.9–74.9)	1.06 (0.98–1.14)	0.12
Diagnosis setting						
Primary care (Reference)	1,118	41.8 (38.9–44.6)	53.3 (50.3–56.2)	77.1 (73.8–80.0)	1	
Secondary care	3,528	45.8 (44.2–47.4)	55.3 (53.7–57.0)	71.0 (69.3–72.7)	0.94 (0.86–1.02)	0.12
Unknown	2	N/A	N/A	N/A	1.99 (1.32–3.00)	0.0011
HF phenotype						
HFrEF (Reference)	609	53.2 (49.2–57.1)	61.1 (57.1–64.8)	77.9 (73.7–81.5)	1	
HFpEF	1,106	52.4 (49.5–55.3)	60.9 (58.0–63.7)	73.8 (70.8–76.6)	0.93 (0.83–1.06)	0.28
Unknown LVEF	2,933	40.3 (38.5–42.0)	51.3 (49.5–53.1)	70.9 (68.9–72.8)	0.79 (0.71–0.88)	<0.0001
NT-proBNP level, pg/mL						
0–1,000 (reference)	912	40.4 (37.2–43.5)	51.8 (48.5–54.9)	72.4 (68.8–75.7)	1	
1,001–3,000	1,283	45.4 (42.7–48.1)	56.6 (53.8–59.2)	76.1 (73.1–78.7)	1.10 (0.99–1.21)	0.07
>3,000	1,601	52.5 (50.0–54.9)	61.3 (58.8–63.6)	74.3 (71.7–76.7)	1.19 (1.08–1.32)	0.0006
Missing	852	34.5 (31.3–37.7)	43.5 (40.2–46.8)	63.2 (59.4–66.9)	0.83 (0.74–0.93)	0.0017

CI, confidence interval; CVD, cardiovascular disease; HF, heart failure; HFpEF, HF with preserved ejection fraction; HFrEF, HF with reduced ejection fraction; HR, hazard ratio; LVEF, left ventricular ejection fraction; NT-proBNP, N-terminal pro-B-type natriuretic peptide.

For peer review only

Note: The Fine and Grays sub-distribution hazard model was used to obtain the sub-distribution HRs. Cumulative incidence function was used to estimate percentage of patients with CVD-related hospitalizations within 0.5, 1, and 3 years after index date.

For peer review only

Table 3 Logistic regression to determine characteristics at HF diagnosis of the 10% of incident patients with HF incurring the greatest secondary healthcare costs during the first year after index date

		Unadjusted model		Adjusted model	
**Characteristics at HF diagnosis**	**N=5,205 n (%)**	**OR**	**95% CI**	**OR**	**95% CI**
Age at index date, years					
18–54	297 (5.7%)	Reference		Reference	
55–64	511(9.8%)	0.88	0.60–1.29	0.83	0.56–1.24
65–74	1,155 (22.2%)	0.89	0.64–1.24	0.82	0.57–1.18
75–84	1,796 (34.5%)	0.40	0.28–0.57	0.39	0.27–0.57
≥85	1,446 (27.8%)	0.18	0.12–0.27	0.21	0.13–0.33
Sex					
Male	2,800 (53.8%)	Reference		Reference	
Female	2,405 (46.2%)	0.73	0.61–0.88	0.98	0.80–1.20
LVEF					
HFrEF	1,167 (22.4%)	Reference		Reference	
HFpEF	652 (12.5%)	1.01	0.79–1.30	1.05	0.80–1.39
Unknown	3,386 (65.1%)	0.29	0.24–0.36	0.41	0.33–0.52
NT-proBNP, ng/L					
0–1,000	1,075 (20.7%)	Reference		Reference	
1,001–3,000	1,433 (27.5%)	1.17	0.88–1.56	1.48	1.09–2.01
>3,001	1,730 (33.2%)	1.77	1.36–2.30	1.93	1.45–2.57
Missing	967 (18.6%)	1.17	0.85–1.60	1.44	1.03–2.02
ACEI or ARB treatment					
ACEI	2,481 (47.7%)	Reference		Reference	
ACEI and ARB	58 (1.1%)	1.81	0.88–3.74	1.61	0.75–3.45
ARB	964 (18.5%)	1.02	0.79–1.31	1.01	0.77–1.33
Neither ACEI nor ARB	1,702 (32.7%)	1.26	1.03–1.55	1.63	1.31–2.03
Hypertension					
No	2,526 (48.5%)	Reference		Reference	
Yes	2,679 (51.5%)	1.11	0.93–1.33	1.06	0.84–1.33
Atrial fibrillation					
No	3,612 (69.4%)	Reference		Reference	
Yes	1,593 (30.61)	0.75	0.61–0.92	0.77	0.61–0.97
Angina or MI					
No	4,007 (76.9%)	Reference		Reference	
Yes	1,198 (23.02%)	1.45	1.19–1.78	1.27	1.00–1.60

For peer review only - http://bmjopen.bmj.com/site/about/guidelines.xhtml

Diabetes					
No	4,253 (81.71%)	Reference		Reference	
Yes	952 (18.29%)	1.48	1.19–1.83	1.19	0.93–1.53
Cancer					
No	4,466 (85.80%)	Reference		Reference	
Yes	739 (14.20%)	1.19	0.93–1.52	1.17	0.89–1.53
Cerebrovascular disease					
No	4,545 (87.32%)	Reference		Reference	
Yes	660 (12.68%)	1.08	0.83–1.41	1.27	0.95–1.71
Dyslipidaemia					
No	4,529 (87.01%)	Reference		Reference	
Yes	676 (12.99%)	1.60	1.26–2.03	1.10	0.83–1.47
Anaemia					
No	4,560 (87.61%)	Reference		Reference	
Yes	645 (12.39%)	1.73	1.36–2.20	1.63	1.24–2.14
COPD					
No	4,801 (92.24%)	Reference		Reference	
Yes	404 (7.76%)	1.27	0.93–1.74	1.13	0.80–1.58
Aortic insufficiency/regurgitation					
No	4,814 (92.49%)	Reference		Reference	
Yes	391 (7.51%)	3.21	2.49–4.13	2.54	1.92–3.37
Dementia					
No	4,883 (93.81%)	Reference		Reference	
Yes	322 (6.19%)	0.33	0.19–0.60	0.53	0.29–0.99
Chronic kidney disease					
No	4,987 (95.81%)	Reference		Reference	
Yes	218 (4.19%)	2.47	1.76–3.48	1.44	0.97–2.14
Depression					
No	5,015 (96.35%)	Reference		Reference	
Yes	190 (3.65%)	1.38	0.90–2.13	1.21	0.75–1.93
Mitral insufficiency/regurgitation					
No	5,030 (96.64%)	Reference		Reference	
Yes	175 (3.36%)	3.32	2.34–4.72	2.58	1.74–3.81
Peripheral artery disease					
No	5,086 (97.71%)	Reference		Reference	
Yes	119 (2.29%)	2.47	1.57–3.87	1.60	0.97–2.65

ACEI, angiotensin-converting enzyme inhibitor; ARB, angiotensin receptor blocker; CI, confidence interval; COPD, chronic obstructive pulmonary disease; HF, heart failure; HFpEF, heart failure with preserved ejection fraction; HFrEF, heart failure with reduced ejection fraction; LVEF, left ventricular ejection fraction; MI, myocardial infarction; NT-proBNP, N-terminal pro-B-type natriuretic peptide; OR, odds ratio.

For peer review only - http://bmjopen.bmj.com/site/about/guidelines.xhtml

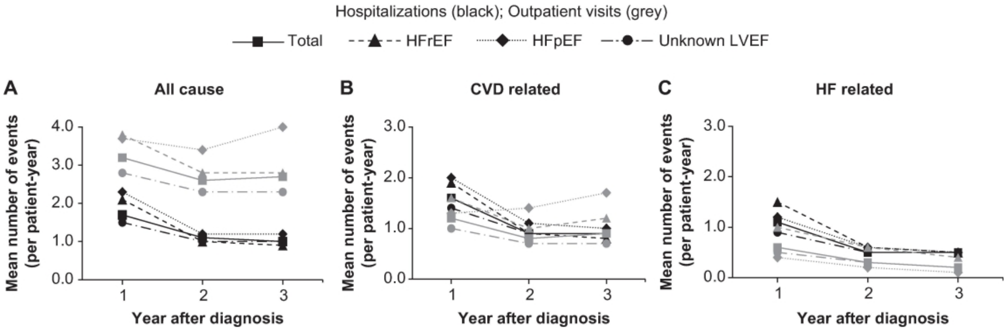

Figure 1 Mean number of hospitalizations (lines in black) and outpatient visits (lines in grey) for incident patients with HF during 2010–2012 by year after HF diagnosis.
CVD, cardiovascular disease; HF, heart failure; HFpEF, HF with preserved ejection fraction; HFrEF, HF with reduced ejection fraction; LVEF, left ventricular ejection fraction.
Notes: Number of patients 1, 2, and 3 years after diagnosis: 4,648, 2,769, and 1,309 total patients; 1,106, 667, and 323 patients with HFrEF; 609, 353, and 156 patients with HFpEF; 2,933, 1,749, and 830 with Unknown LVEF, respectively.

177x57mm (600 x 600 DPI)

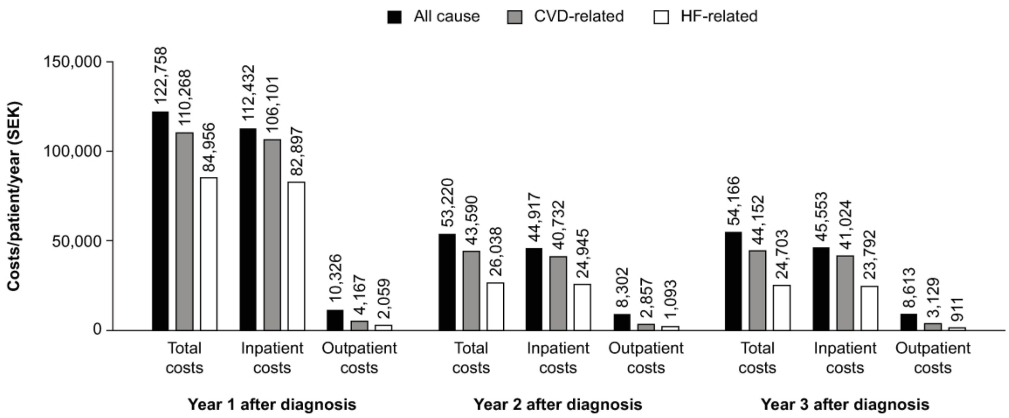

Figure 2 All-cause, CVD-related, and HF-related total, inpatient, and outpatient costs up to 3 years after diagnosis of HF (incident patients with HF, 2010–2012).
CVD, cardiovascular disease; HF, heart failure; SEK, Swedish krona.
Notes: Number of patients: year 1, n=4,648; year 2, n=2,769; year 3, n=13,095. All costs are given in SEK 2015 values (1 SEK = €0.105).

172x70mm (600 x 600 DPI)

Supporting information

Secondary healthcare costs: pharmacotherapy (the Uppsala County cohort)

In the cohort of patients from the Uppsala County with incident patients with heart failure (HF) during the period of 2010–2012 (N=3,452), the overall drug use (HF-related drugs and other pharmacotherapies) in the year after diagnosis was associated with a mean cost of Swedish krona (SEK) 10,727 (equivalent to €1,126) per patient per year. In general, these costs remained stable for up to 4 years after the diagnosis of HF. However, at year 5, an increase was observed in the mean cost to SEK 12,760 (€1,340) per patient per year. The costs of HF-specific therapy (drugs listed in Table S2) constituted 13% of the total drug costs in the first year after HF diagnosis (mean costs per patient per year at years 1 and 5 after HF diagnosis were SEK 1,395 [€146] and 1,404 [€147], respectively).

For peer review only - http://bmjopen.bmj.com/site/about/guidelines.xhtml

Table S1 List of ICD-10 codes

**Comorbidity/risk factor**	**ICD-10 code**
Hypertension	I10–I13, I15
Atrial fibrillation	I48
Angina or MI	I20–I22
Diabetes	E10–E14
Cancer	C00–C97
Cerebrovascular disease	I60–I69
Dyslipidaemia	E78
Anaemia	D50–D64
COPD	J40–J44, J47
Aortic insufficiency/regurgitation	I06, I35, Q231
Dementia	F00–F07
Chronic kidney disease	N18
Depression	F32–F33
Mitral insufficiency/regurgitation	I051, I340, Q233
Peripheral artery disease	I739
**Definitions for calculation of CCI**	
MI	I21–I22, I252
Congestive heart failure	I43, I50, I099, I110, I130, I132, I255, I420, I425–I429, P290
Peripheral vascular disease	I70–I71, I731, I738–I739, I771, I790, I792, K551, K558–K559, Z958–Z959

For peer review only

Cerebrovascular disease	G45–G46, I60–I69, H340
Dementia	F00–F03, G30, F051, G311
Chronic pulmonary disease	J40–J47, J60–J67, I278–I279, J684, J701, J703
Connective tissue disease-rheumatic disease	M05–M06, M315, M32–M34, M351, M353, M360
Peptic ulcer disease	K25–K28
Mild liver disease	B18, K73–K74, K700–K703, K709, K713–K715, K717, K760, K762–K764, K768–K769, Z944
Diabetes without complications	E100–E101, E106, E108–E111, E116, E118–E121, E126, E128–E131, E136, E138–E141, E146, E148–E149
Diabetes with complications	E102–E105, E107, E112–E115, E117, E122–E125, E127, E132–E135, E137, E142–E145, E147
Paraplegia and hemiplegia	G81–G82, G041, G114, G801–G802, G830–G834, G839
Renal disease	N18–N19, N052–N057, N250, I120, I131, N032–N037, Z490–Z492, Z940, Z992
Cancer	C00–C76, C81–C97
Moderate to severe liver disease	K704, K711, K721, K729, K765–K767, I850, I859, I864, I982
Metastatic carcinoma	C77–C80
AIDS/HIV	B20–B22, B24

AIDS/HIV, human immunodeficiency virus/acquired immunodeficiency syndrome; CCI, Charlson comorbidity index; COPD, chronic obstructive pulmonary disease; ICD-10, International Classification of Diseases and Related Health Problems, Tenth Revision; MI, myocardial infarction.

For peer review only - http://bmjopen.bmj.com/site/about/guidelines.xhtml

Table S2 Heart failure-related drugs (according to the European Society of Cardiology 2012 guidelines[9])

**Drug/drug class**	**Anatomical Therapeutic Chemical code**
Angiotensin-converting enzyme inhibitor	C09A, C09B
Angiotensin receptor blocker	C09C, C09D
Beta blocker	C07
Mineralocorticoid receptor antagonist	C03DA01-C03DA04
Ivabradine (sinus node I_f channel inhibitor)	C01EB17
Diuretics	C03
Digoxin	C01AA05
Nitrates and nitrites	C01DA

For peer review only

Table S3 Sub-distribution hazard model of the proportion of incident patients with HF from the Uppsala and Västerbotten counties with a second HF diagnosis in 2010–2012 who had all-cause hospitalizations within 3 years and cumulative incidence function estimates for the proportion of hospitalized patients within 0.5, 1, and 3 years after index date

**Characteristic**	**N=4,648**	**Cumulative incidence function of all-cause hospitalizations, % (95% CI)**			**Sub-distribution HR (95% CI)**	***P* value**
		**0.5 years**	**1 year**	**3 years**		
Age group, years						
18–54 (reference)	275	51.6 (45.6–57.4)	58.2 (52.1–63.8)	69.5 (63.0–75.0)	1	
55–64	457	50.3 (45.6–54.8)	60.4 (55.7–64.7)	75.2 (70.1–79.5)	1.07 (0.88–1.29)	0.50
65–74	1,051	50.5 (47.5–53.5)	60.7 (57.7–63.6)	75.8 (72.7–78.6)	1.11 (0.93–1.31)	0.24
75–84	1,592	46.2 (43.8–48.7)	57.4 (54.9–59.8)	77.3 (74.7–79.8)	1.06 (0.90–1.25)	0.51
≥85	1,273	43.4 (40.6–46.1)	53.7 (51.0–56.4)	72.4 (69.5–75.0)	0.97 (0.82–1.16)	0.77
Sex						
Female (reference)	2,143	46.3 (44.2–48.4)	55.6 (53.5–57.7)	74.5 (72.2–76.6)	1	
Male	2,505	47.8 (45.9–49.8)	59.1 (57.1–61.0)	75.5 (73.4–77.4)	1.04 (0.97–1.12)	0.28

For peer review only - http://bmjopen.bmj.com/site/about/guidelines.xhtml

Diagnosis setting						
Primary care (reference)	1,118	43.7 (40.8–46.6)	56.1 (53.1–58.9)	79.9 (76.7–82.8)	1	
Secondary care	3,528	48.2 (46.5–49.8)	57.9 (56.3–59.5)	73.6 (71.8–75.2)	0.93 (0.86–1.01)	0.07
Unknown	2	N/A	N/A	N/A	1.80 (1.15–2.82)	0.0098
HF phenotype						
HFrEF (reference)	609	56.5 (52.4–60.3)	64.7 (60.8–68.4)	80.5 (76.5–83.9)	1	
HFpEF	1,106	54.9 (51.9–57.8)	63.7 (60.8–66.5)	76.4 (73.4–79.0)	0.91 (0.81–1.03)	0.14
Unknown LVEF	2,933	42.3 (40.5–44.1)	53.6 (51.8–55.4)	73.6 (71.6–75.4)	0.77 (0.70–0.86)	<0.0001
NT-proBNP, pg/mL						
0–1,000 (reference)	912	43.0 (39.7–46.2)	55.2 (51.9–58.3)	76.4 (73.0–79.6)	1	
1,001–3,000	1,283	47.3 (44.6–50.0)	58.6 (55.9–61.3)	78.0 (75.2–80.6)	1.06 (0.97–1.17)	0.21
>3,000	1,601	54.5 (52.0–56.9)	63.7 (61.3–66.0)	76.4 (73.9–78.7)	1.19 (1.07–1.31)	0.0007
Missing	852	37.6 (34.3–40.8)	46.6 (43.2–49.9)	66.2 (62.5–69.7)	0.84 (0.75–0.94)	0.0028

For peer review only

CI, confidence interval; HF, heart failure; HFpEF, HF with preserved ejection fraction; HFrEF, HF with reduced ejection fraction; HR, hazard ratio; LVEF, left ventricular ejection fraction; NT-proBNP, N-terminal pro-B-type natriuretic peptide.

Note: Multivariate sub-distribution hazard model with death as competing risk used to obtain the sub-distribution HRs. Cumulative incidence function was used to estimate percentage of patients with all-cause hospitalizations in 0.5, 1, and 3 years after index date.

For peer review only

For peer review only - http://bmjopen.bmj.com/site/about/guidelines.xhtml

Table S4 Costs associated with primary care contacts for incident patients with HF between 2010 and 2012 in the Uppsala County

	**Mean (SD) costs per patient per year in SEK**				
	**Year 1 (N=3,453)**	**Year 2 (N=2,705)**	**Year 3 (N=1,846)**	**Year 4 (N=891)**	**Year 5 (N=166)**
Total costs	10,347 (12,685)	9,521 (11,974)	9,490 (12,225)	9,754 (12,755)	10,869 (16,857)
Family physician visits	5,432 (8,634)	4,718 (7,723)	4,668 (7,652)	4,641 (7,693)	5,153 (7,714)
Nurse visits	4,050 (7,251)	4,068 (7,475)	4,100 (7,546)	4,388 (8,107)	4,992 (11,685)
Blood tests	866 (1,254)	736 (1,143)	722 (1,133)	726 (1,128)	724 (1,034)

HF, heart failure; SD, standard deviation; SEK, Swedish krona.

Notes: Primary care costs (available only for the Uppsala County cohort) per visit or per test were calculated based on published price lists. All costs are in SEK 2015 values (1 SEK = €0.105).

For peer review only - http://bmjopen.bmj.com/site/about/guidelines.xhtml

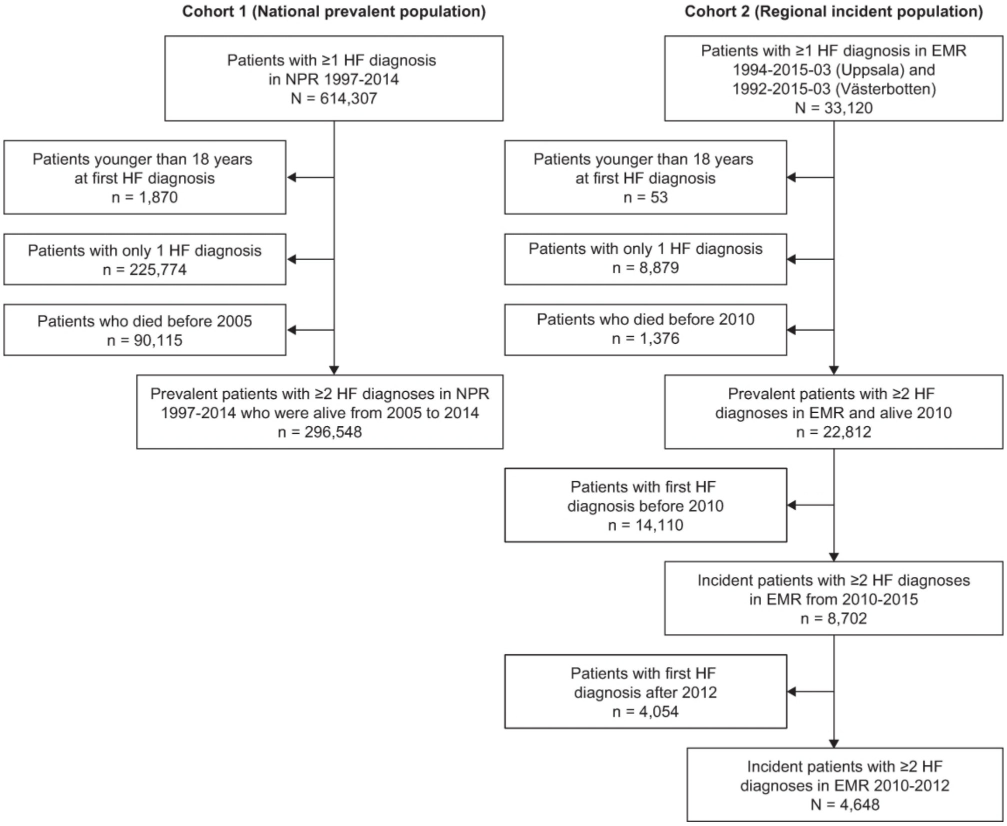

Figure S1: Patient flow. EMR, electronic medical record; HF, heart failure; HFpEF, HF with preserved ejection fraction; HFrEF, HF with reduced ejection fraction; LVEF, left ventricular ejection fraction; NPR, National Patient Register. %"Notes: In cohort 1, data from 614,307 patients were extracted from the Swedish NPR on the basis of an observed HF diagnosis between 1997 and 2014; of which, data from 296,548 patients met the inclusion criteria of at least two HF diagnoses in the analysis period (2005–2014) with no HF diagnosis in the look-back period (1997–2004). In cohort 2, data from 33,120 patients were extracted from the EMRs on the basis of an observed HF diagnosis between 1994 and 2015 (Uppsala County) and between 1992 and 2015 (Västerbotten County); of which, data from 4,648 patients met the inclusion criteria of at least two HF diagnoses in the analysis period (2010–2012) and no HF diagnosis in the look-back period (NPR: 1997–2009, EMR: 1992–2009). A total of 4,648 analyzable patients with 3-year follow-up data were identified between 2010 and 2012. Of the 1,715 patients for whom LVEF data were available, 64,5% had HFrEF and 35,5% had HFpEF. The incident HF population comprised those with no HF diagnosis in the look-back periods.

177x145mm (600 x 600 DPI)

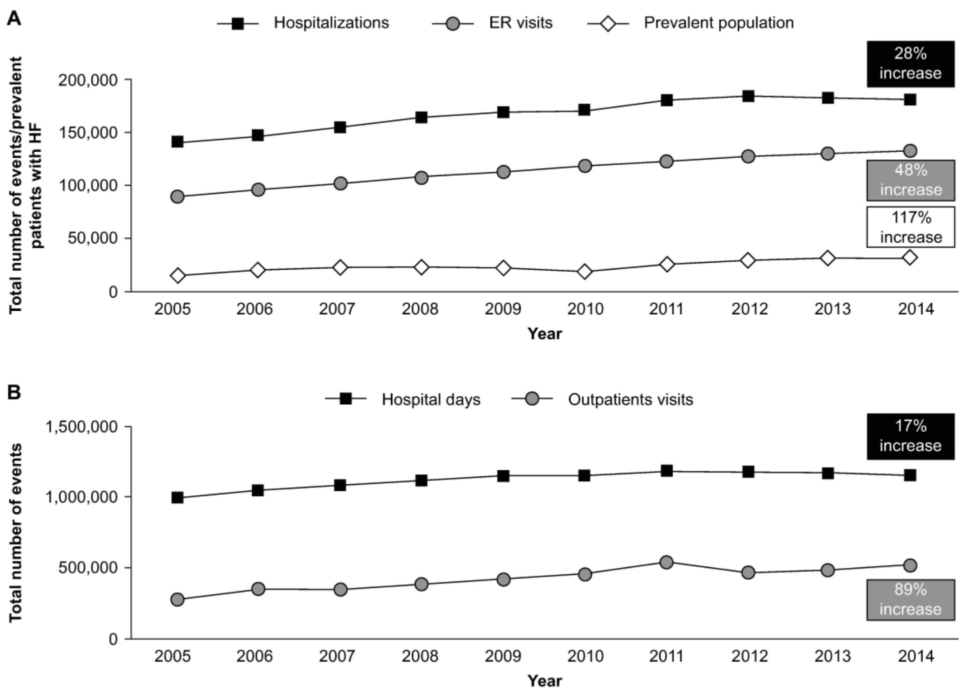

	2005	2006	2007	2008	2009	2010	2011	2012	2013	2014
Persons >18 years old in Sweden, n	7,113,513	7,179,337	7,251,275	7,331,508	7,419,589	7,496,476	7,563,649	7,627,772	7,692,386	7,762,073
Age-adjusted prevalence of HF in Sweden, % (CI)	1.30 (1.31–1.29)	1.37 (1.38–1.36)	1.44 (1.45–1.43)	1.51 (1.52–1.50)	1.56 (1.57–1.55)	1.61 (1.62–1.60)	1.66 (1.67–1.65)	1.69 (1.70–1.69)	1.71 (1.72–1.70)	1.72 (1.72–1.71)

Figure S2: Total number of (a) hospitalizations and ER visits and (b) hospital days and outpatient visits in relation to the absolute number of prevalent adult patients with HF extracted from the NPR, size of adult population in Sweden, and age-adjusted prevalence of HF in Sweden from 2005 to 2014. CI, confidence interval; ER, emergency room; HF, heart failure; NPR, National Patient Register.

171x134mm (600 x 600 DPI)

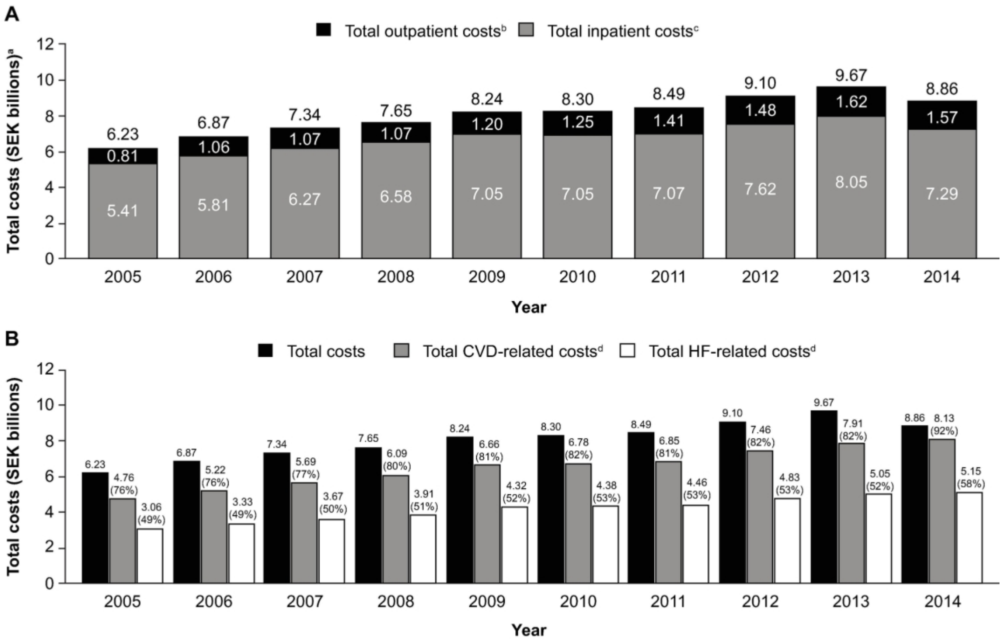

Figure S3 Costs associated with secondary care of prevalent patients with HF from 2005 to 2014: (a) total, inpatient, and outpatient costs and (b) total, CVD-related, and HF-related costs.
CVD, cardiovascular disease; ER, emergency room; HF, heart failure; SEK, Swedish krona.
Notes: aNumbers above bars indicate total costs (sum of inpatient and outpatient costs). bTotal inpatient costs correspond to the costs associated with hospitalizations. cTotal outpatient costs are the sum of the costs associated with outpatient and ER visits. dCVD-related and HF-related costs are not mutually exclusive; percentages shown in brackets represent the proportion of CVD-related and HF-related costs from the total costs.

169x108mm (300 x 300 DPI)

STROBE 2007 (v4) Statement—Checklist of items that should be included in reports of *cohort studies*

Section/Topic	Item #	Recommendation	Reported on page #
**Title and abstract**	1	(*a*) Indicate the study's design with a commonly used term in the title or the abstract	1
		(*b*) Provide in the abstract an informative and balanced summary of what was done and what was found	2
**Introduction**			
Background/rationale	2	Explain the scientific background and rationale for the investigation being reported	4
Objectives	3	State specific objectives, including any prespecified hypotheses	4
**Methods**			
Study design	4	Present key elements of study design early in the paper	4-5
Setting	5	Describe the setting, locations, and relevant dates, including periods of recruitment, exposure, follow-up, and data collection	4-5
Participants	6	(*a*) Give the eligibility criteria, and the sources and methods of selection of participants. Describe methods of follow-up	5
		(*b*) For matched studies, give matching criteria and number of exposed and unexposed	NA
Variables	7	Clearly define all outcomes, exposures, predictors, potential confounders, and effect modifiers. Give diagnostic criteria, if applicable	7-8
Data sources/ measurement	8*	For each variable of interest, give sources of data and details of methods of assessment (measurement). Describe comparability of assessment methods if there is more than one group	7-8
Bias	9	Describe any efforts to address potential sources of bias	6
Study size	10	Explain how the study size was arrived at	NA
Quantitative variables	11	Explain how quantitative variables were handled in the analyses. If applicable, describe which groupings were chosen and why	7-8
Statistical methods	12	(*a*) Describe all statistical methods, including those used to control for confounding	8
		(*b*) Describe any methods used to examine subgroups and interactions	7-8
		(*c*) Explain how missing data were addressed	NA
		(*d*) If applicable, explain how loss to follow-up was addressed	NA
		(*e*) Describe any sensitivity analyses	NA
**Results**			

For peer review only

Participants	13*	(a) Report numbers of individuals at each stage of study—eg numbers potentially eligible, examined for eligibility, confirmed eligible, included in the study, completing follow-up, and analysed	8-9
		(b) Give reasons for non-participation at each stage	NA
		(c) Consider use of a flow diagram	9 (Figure S1)
Descriptive data	14*	(a) Give characteristics of study participants (eg demographic, clinical, social) and information on exposures and potential confounders	9
		(b) Indicate number of participants with missing data for each variable of interest	NA
		(c) Summarise follow-up time (eg, average and total amount)	6
Outcome data	15*	Report numbers of outcome events or summary measures over time	8-12
Main results	16	(*a*) Give unadjusted estimates and, if applicable, confounder-adjusted estimates and their precision (eg, 95% confidence interval). Make clear which confounders were adjusted for and why they were included	8
		(*b*) Report category boundaries when continuous variables were categorized	NA
		(*c*) If relevant, consider translating estimates of relative risk into absolute risk for a meaningful time period	NA
Other analyses	17	Report other analyses done—eg analyses of subgroups and interactions, and sensitivity analyses	9
**Discussion**			
Key results	18	Summarise key results with reference to study objectives	12-15
**Limitations**			
Interpretation	20	Give a cautious overall interpretation of results considering objectives, limitations, multiplicity of analyses, results from similar studies, and other relevant evidence	14-15
Generalisability	21	Discuss the generalisability (external validity) of the study results	12-15
**Other information**			
Funding	22	Give the source of funding and the role of the funders for the present study and, if applicable, for the original study on which the present article is based	15

*Give information separately for cases and controls in case-control studies and, if applicable, for exposed and unexposed groups in cohort and cross-sectional studies.

Note: An Explanation and Elaboration article discusses each checklist item and gives methodological background and published examples of transparent reporting. The STROBE checklist is best used in conjunction with this article (freely available on the Web sites of PLoS Medicine at http://www.plosmedicine.org/, Annals of Internal Medicine at http://www.annals.org/, and Epidemiology at http://www.epidem.com/). Information on the STROBE Initiative is available at www.strobe-statement.org.

For peer review only

BMJ Open

Healthcare resource utilization and costs associated with a heart failure diagnosis: a retrospective, population-based cohort study in Sweden.

Journal:	*BMJ Open*
Manuscript ID	bmjopen-2021-053806.R1
Article Type:	Original research
Date Submitted by the Author:	28-Jul-2021
Complete List of Authors:	Boman, Kurt; Umea University Department of Public Health and Clinical Medicine, Research Unit, Medicine<br>Lindmark, Krister; Umea University, Dept of public health and clinical medicine and Heart Centre<br>Stålhammar, Jan; Uppsala University, Dept of Public Health and Caring Sciences<br>Olofsson, Mona; Umea University Department of Public Health and Clinical Medicine, Research Unit, Medicine<br>Costa-Scharplatz, Madlaina; Novartis Sweden AB, Medical Affairs/RWE<br>Fonseca, Ana Filipa; Novartis Pharma AG<br>Johansson, Stina; IQVIA<br>Heller, Vincent; IQVIA<br>Törnblom, Michael; IQVIA Solutions Sweden AB, Real-World & Analytics Solutions<br>Wikström, Gerhard; Uppsala University Hospital, Institute of Medical Sciences
**Primary Subject Heading**:	Health economics
Secondary Subject Heading:	Health economics
Keywords:	Heart failure < CARDIOLOGY, Adult cardiology < CARDIOLOGY, HEALTH ECONOMICS

SCHOLARONE™
Manuscripts

I, the Submitting Author has the right to grant and does grant on behalf of all authors of the Work (as defined in the below author licence), an exclusive licence and/or a non-exclusive licence for contributions from authors who are: i) UK Crown employees; ii) where BMJ has agreed a CC-BY licence shall apply, and/or iii) in accordance with the terms applicable for US Federal Government officers or employees acting as part of their official duties; on a worldwide, perpetual, irrevocable, royalty-free basis to BMJ Publishing Group Ltd ("BMJ") its licensees and where the relevant Journal is co-owned by BMJ to the co-owners of the Journal, to publish the Work in this journal and any other BMJ products and to exploit all rights, as set out in our licence.

The Submitting Author accepts and understands that any supply made under these terms is made by BMJ to the Submitting Author unless you are acting as an employee on behalf of your employer or a postgraduate student of an affiliated institution which is paying any applicable article publishing charge ("APC") for Open Access articles. Where the Submitting Author wishes to make the Work available on an Open Access basis (and intends to pay the relevant APC), the terms of reuse of such Open Access shall be governed by a Creative Commons licence – details of these licences and which Creative Commons licence will apply to this Work are set out in our licence referred to above.

Other than as permitted in any relevant BMJ Author's Self Archiving Policies, I confirm this Work has not been accepted for publication elsewhere, is not being considered for publication elsewhere and does not duplicate material already published. I confirm all authors consent to publication of this Work and authorise the granting of this licence.

For peer review only

Healthcare resource utilization and costs associated with a heart failure diagnosis: a retrospective, population-based cohort study in Sweden.

Kurt Boman,[1] Krister Lindmark,[2] Jan Stålhammar,[3] Mona Olofsson,[1] Madlaina Costa-Scharplatz,[4] Ana Filipa Fonseca,[5] Stina Johansson,[6] Vincent Heller,[6] Michael Törnblom,[6] Gerhard Wikström[7]

[1]Research Unit, Medicine-Geriatric, Skellefteå County Hospital, Department of Public Health and Clinical Medicine, Umeå University, Umeå, Sweden; [2]Department of Public Health and Clinical Medicine and Heart Centre, Umeå University Hospital, Umeå, Sweden; [3]Department of Public Health and Caring Sciences, Uppsala University, Uppsala, Sweden; [4]Medical Affairs/RWE, Novartis Sweden AB, Stockholm, Sweden; [5]Novartis Pharma AG, Basel, Switzerland; [6] Real-World & Analytics Solutions, IQVIA, Solna, Sweden; [7]Institute for Medical Sciences, Uppsala University, Uppsala, Sweden

Corresponding author

Kurt Boman

Department of Public health and Clinical Medicine,

Umeå University, SE-901 87

Umea, Sweden

Sweden

Tel: +46910771113

Fax: +4691010274

Email: Kurt.Boman@regionvasterbotten.se

For peer review only

Abstract

Objectives: To examine healthcare resource use (HRU) and costs among heart failure (HF) patients using population data from Sweden.

Design: Retrospective, non-interventional cohort study

Setting: Two cohorts were identified from linked national health registers (cohort 1, 2005–2014) and electronic medical records (cohort 2, 2010–2012; primary/secondary care patients from Uppsala and Västerbotten).

Participants Patients (aged ≥18 years) with primary or secondary diagnoses of HF (≥2 ICD-10 classification) during the identification period of January 2005 to March 2015 were included.

Outcome measures: HRU across the HF phenotypes was assessed with logistic regression. Costs were estimated based on diagnosis-related group codes and general price lists.

Results: Total annual costs of secondary care of prevalent HF increased from SEK 6.23 (€0.60) to 8.86 (€0.85) billion between 2005 and 2014. Of 4,648 incident patients, HF phenotype was known for 1,715: reduced ejection fraction [HFrEF]: 64.5%, preserved ejection fraction [HFpEF]: 35.5%. Within 1 year of HF diagnosis, the proportion of patients hospitalized was only marginally higher for HFrEF vs HFpEF (all-cause [95%CI]: 64.7% [60.8–68.4] vs 63.7% [60.8–66.5], hazard ratio [HR] 0.91, P=0.14; cardiovascular disease related [95%CI]: 61.1% [57.1–64.8] vs 60.9% [58.0–63.7], HR 0.93, P=0.28). Frequency of hospitalizations and outpatient visits per patient declined after the first year. All-cause secondary care costs in the first year were SEK 122,758 (€12,890)/patient/year, with HF-specific care accounting for 69% of the costs. Overall, 10% of the most expensive population (younger; predominantly male; more likely to have comorbidities) incurred ~40% of total secondary care costs.

For peer review only

Conclusions: HF-associated costs and HRU are high, especially during the first year of diagnosis. This is driven by high hospitalizations rates. Understanding the profile of resource-intensive patients being at younger age, male sex, and high Charlson comorbidity index (CCI) scores at the time of the HF diagnosis is most likely a sign of more severe disease.

Key words: Heart failure, resource use, cost, HF phenotypes, real-world

Strengths and limitations of this study

- The study provides comprehensive information from two county regions in Sweden on the increase in number of newly diagnosed HF patients, resource use across the HF phenotypes and total annual costs of secondary care of prevalent HF in Sweden.
- The study provides new insights about the profile of resource-intensive patients at the time of the HF diagnosis, which can support guidance in resource allocation and improve patient management.
- A retrospective identification of patients with confirmed HF despite the requirement for at least two HF diagnoses is challenging.
- Primary and secondary care resource use and costs were available only for the Uppsala Region cohort, which limited the direct comparison with the data from the Västerbotten Region where only secondary care resources were available.
- Our data reflect the real-world costs associated with a diagnosis of HF, despite an acknowledged limitation in the true validation of the diagnosis due to lack of echocardiogram records for the majority of patients.

Introduction

For peer review only

Heart failure (HF) represents a major public health challenge in developed countries and is associated with high healthcare resource consumption[1] with cardiovascular diseases (CVD) as one of the leading cause of mortality in Sweden.[2] HF places a considerable burden on the healthcare systems,[3, 4] and approximately 20% of patients discharged from hospitals after an HF admission were readmitted within 30 days between 2010 and 2013.[5] Inpatient HF care accounts for the largest proportion of healthcare costs,[6] and that the total annual direct cost related to HF is Swedish krona 2.6 billion, corresponding to approximately 2% of the total Swedish healthcare budget.[7]

An ageing population, increasing number of comorbidities, and changed treatment patterns necessitate the examination of healthcare resource use and costs. Therefore, our primary aim was to investigate trends in total healthcare costs from 2005 to 2014 among prevalent and incident patients with HF[8]. Patient characteristics in the context of resource use were analyzed to determine which individuals incurred the greatest resource and secondary care costs. With the growing importance of HF with preserved ejection fraction (HFpEF), this investigation goes a step further than other Swedish studies[3, 4] to present new data comparing resource use and costs among patients according to HF phenotype.

Methods

Study design

This was a retrospective, non-interventional cohort study using longitudinal patient-level data for patients with HF in Sweden.[9] Data were collected from electronic medical records (EMRs) from primary and secondary care and from local echocardiography registries in the counties of Uppsala (two hospitals and 46 primary care centres [PCCs]) and Västerbotten (three hospitals and 37 PCCs). EMR data were linked via unique identifiers issued by the National Board of Health and Welfare (NBHW) to data from national health registers, including the National Patient Register (NPR). Data were pseudonymized before the linked

For peer review only

database was released to the research group, and ethical approval was obtained from the regional Ethical Review Board in Uppsala, Sweden (2015-045) before data extraction. No informed consent was required for this retrospective, pseudonymized study.

Patients

Patients (aged ≥18 years) with primary or secondary diagnoses of HF during the identification period of January 2005 to March 2015 were included. Analyzing data from patients treated in both primary and secondary care by linking NPR data with EMR data presents the challenge of distinct HF diagnosis specificity across the two settings. Two HF diagnoses were required to reduce the uncertainty surrounding HF diagnosis in primary care and increase overall diagnosis specificity. Diagnosis of HF was based on the International Classification of Diseases and Related Health Problems, tenth revision (ICD-10) diagnostic codes of I50, I42.0, I42.6, I42.7, I42.9, I11.0, I13.0, or I13.2 for the primary or secondary diagnoses (Table S1).

The national cohort comprised prevalent patients with HF registered in the NPR from secondary care between January 2005 and December 2014, while the regional cohort (Uppsala and Västerbotten) included incident patients with HF diagnoses registered in EMRs from either primary or secondary care between January 2010 and March 2015.

The Pygargus Customized eXtraction Program (CXP 3.0) extracted data from EMRs from Uppsala and Västerbotten and subsequently linked to data from the national health registers including the NPR.[10] HF with preserved ejection fraction [HFpEF] and HF with reduced ejection fraction [HFrEF]) was determined based only on data from local echocardiography registries, and for cohort 2, HFpEF was defined as a left ventricular ejection fraction (LVEF) of ≥50% and HFrEF was defined as an LVEF of <50%. This cut-off is based on data constraints; LVEF is recorded categorically in the echocardiography

For peer review only

registers, with the categories used differing between the county councils such that 50% was the only option that allowed for a common threshold.

Data extraction and study timelines

Data were extracted from EMRs based on an observed HF diagnosis during 1994-2015 for the Uppsala County and during 1992–2016 for the Västerbotten County. The analysis period was adjusted to 1 January 2010 owing to inconsistent patterns of HF registration in the Uppsala County EMR systems. The look-back period in the EMR data was from the time the first data were available until 31 December 2009. For NPR data, the look-back period was from 1 January 1997 for inpatient care and 1 January 2001 for outpatient care to 31 December 2004 (Figure S1) to identify prevalent patients with HF at a national level. This prevalent patient cohort was used to assess trends in total healthcare resource use and costs from 2005 to 2014.

Newly diagnosed individuals from the Uppsala County and during the analysis period made up the incident HF population. To enable all patients to have at least 3 years of potential follow-up, all analyses were performed on patients diagnosed with incident HF between 2010 and 2012 (Figure S1).

For the analyses of clinical characteristics, healthcare resource use, and costs, the index date was defined as the date of first HF diagnosis. However, for the analyses of hospitalizations, the index date was defined as a patient's second HF diagnosis to improve the accuracy of diagnosis and minimize the effects of immortal time bias. Follow-up was defined as the period between the second HF diagnosis and the end of the study, end of EMR collection for those patients who moved to another region, or date of death, whichever came first.

For peer review only

Variables analyzed and statistical analyses

For categorical outcomes a χ^2 test was performed; for comparison of means, a *t*-test was performed and confidence intervals (CIs) were calculated at a 95% level. Descriptive statistics assessed baseline clinical characteristics (Table 1) on the date of first HF diagnosis, since this was truly the first time that the patients were considered to have HF. Comorbidities, data from primary (EMR) and secondary (EMR and NPR) care stratified according to HF phenotype, were summarized according to the ICD-10 codes (Table S1). Comorbidities were collected from visits that occurred 0–5 years before the date of the first HF diagnosis. Charlson comorbidity index (CCI) scores during this time, ranging from 0 to greater than 10 (higher scores indicate greater comorbidity), were calculated.

All-cause CVD-related hospitalizations were analyzed using a subdistribution hazard model, proportion of patients having all-cause CVD-related hospitalizations occurring within 0.5, 1 and 3 years after the second HF diagnosis were estimated by a cumulative incidence function.. The time to all-cause and time to CVD-related hospitalizations/readmissions were estimated from the date of the second HF diagnosis using a Cox proportional hazards model, with age group, sex, diagnosis setting (primary care, secondary care, unknown), HF phenotype, and N-terminal pro B-type natriuretic peptide (NT-proBNP) level (0–1,000, 1,001–3,000, and >3,000 pg/mL) as covariates. Data for the mean and median (interquartile range [IQR]) number of hospitalizations and outpatient visits for all-cause, CVD-related, and HF-related events, as well as mean length of stay (LOS), were analyzed by HF phenotype and year since HF diagnosis.

Costs were estimated by year since the first HF diagnosis. All costs are presented as SEK 2015 values (based on the Consumer Price Index,[11] with the equivalent value in euros [€], based on the historical exchange rate in January 2015 (1 SEK = €0.105). Analyses of all-cause, CVD-related, and HF-related costs associated with secondary care were performed

For peer review only - http://bmjopen.bmj.com/site/about/guidelines.xhtml

based on diagnosis-related group codes and price lists, as determined by the NBHW.[12] Costs associated with primary care comprised costs of family physician and nurse visits as well as costs of blood tests. Primary care costs were based on general price lists and were available for the Uppsala County only, as were costs related to pharmacotherapy.[13] The total cost of all drug use for incident patients with HF was estimated, as was the aggregated cost of HF-related drugs, based on the 2012 European Society of Cardiology (ESC) guidelines (Table S2).[14] Logistic regression assessed baseline characteristics of patients who incurred the greatest secondary care costs related to HF, defined as the top 10% most resource-intensive patients.

SAS version 9.3 or higher was used for statistical analysis and data management.

Patient and Public Involvement

No patients or members of the public were involved in this study.

Results

National prevalent population with HF

The absolute number of prevalent patients with HF in Sweden increased by 1.5-fold between 2005 and 2014 from 89,837 to 133,220 (overall population increased from 7,113,513 to 7,762,073), with a corresponding increase in age-adjusted prevalence from 1.30% to 1.72%.

Increase in total costs of prevalent patients with HF over time.

The total number of hospitalizations, hospital days, emergency room (ER) visits, and outpatient visits in secondary care increased from 2005 to 2014 by between 1.2- and 2.2-fold (Figure S2). The number of all-cause hospitalizations increased from 141,941 to 181,374 (difference: 39,433; 28% increase). The number of hospital days increased from 998,512 to 1,165,310 (difference: 166,798; 17% increase), indicating a decreased mean LOS of 4.23

For peer review only

days per hospitalization. ER visits increased from 14,272 to 31,037 (difference: 16,765; 117% increase). Outpatient visits in secondary care increased from 262,771 to 497,230 (difference: 234,459; 89% increase).

The total annual costs associated with the secondary care of prevalent patients with HF increased by 1.4-fold from SEK 6.23 billion (€0.60 billion) in 2005 to SEK 8.86 billion (€0.85 billion) in 2014, mainly driven by a corresponding increase in the overall resource use in inpatient care (52%) (Figure S3a). On an average, inpatient and outpatient costs accounted for 84% and 16% of the total costs, respectively. On an average, 81% of the total costs of secondary care were attributed to CVD-related costs; 64% of these were attributed to HF-related costs (Figure S3b). HF-related costs accounted for 52% of the total costs.

Regional incident population with HF

Data from 104,562 patients were identified from the Uppsala and Västerbotten counties; among these, 8,702 (8.3%) patients had a defined HF diagnosis. Information was available for 4,648 incident patients with HF between 2010 and 2012 (Figure S1). HF phenotype was characterized for 1,715 patients (36.9%); 1,106 (64.5%) patients had HFrEF and 609 (35.5%) had HFpEF (Figure S1).

Patient demographics and clinical characteristics

Table 1 presents the baseline clinical characteristics of patients, overall and by HF phenotype. Mean (median, IQR) age of the patients was 76.8 (79.0, 69.5–86.7) years, more than one-quarter (27.8%) were aged 85 years or more, and 53.8% were male. Most first HF diagnoses occurred in secondary care (75.9%) rather than in primary care (24.1%) (Table 2).

For peer review only

Hospitalizations

All-cause and CVD-related hospitalizations

The cumulative incidence function (95% CI) of the proportion of patients with all-cause hospitalizations within the first year of HF diagnosis was 64.7% (60.8–68.4) for patients with HFrEF and 63.7% (60.8–66.5) for patients with HFpEF; the corresponding sub-distribution hazard ratio (HR) over the follow-up period for HFrEF compared with HFpEF was 0.91 (95% CI: 0.81–1.03; *P*=0.14) (Table S3). The cumulative incidence function of the proportion of patients with all-cause hospitalizations was similar for patients who had their first HF diagnosis in secondary vs primary care; the corresponding sub-distribution HR for the follow-up period for secondary vs primary care was 0.93 (95% CI: 0.86–1.01; *P*<0.07) (Table S3). Higher NT-proBNP levels were associated with a greater risk of all-cause hospitalizations (Table S3), also seen for the proportion of patients with CVD-related hospitalizations by HF phenotype, diagnosis setting, NT-proBNP level, and age (Table 2).

Number of hospitalizations and outpatient visits and LOS

Mean and median number of all-cause, CVD-related, and HF-related hospitalizations and outpatient visits per patient decreased from the first year of HF diagnosis and stabilized between years 2 and 3 (Figure 1). Similar trends in hospitalizations and outpatient visits compared with those observed in the overall population were observed for patients with HFrEF and HFpEF, except that in patients with HFpEF, the mean number of CVD-related outpatient visits increased from the first year after HF diagnosis (Figure 1). The mean (median, IQR) LOS for all-cause, CVD-related, and HF-related hospitalizations was 17.5 (8, 2–23), 16.5 (8, 2–22), and 12.8 (6, 0–16) days in the first year after HF diagnosis, declining at 3 years after diagnosis to 7.6 (0, 0–8), 7.0 (0, 0–7), and 4.2 (0, 0–2) days, respectively.

For peer review only

Patients with HFrEF had a shorter LOS for all-cause, CVD-related, and HF-related events than those with HFpEF (data not shown).

Healthcare costs per patient for the first year after the index date

Secondary healthcare (overall, inpatient, and outpatient) costs

Total and inpatient all-cause costs decreased by more than half after the first year of HF diagnosis (Figure 2). A reduction in all-cause outpatient costs was also noted at the second year after HF diagnosis, relative to the first year. Similar trends were seen for CVD-related and HF-related costs. In the first year after HF diagnosis, total all-cause secondary care costs were SEK 122,758 (€12,890) per patient per year, which declined to SEK 53,220 (€5,588) per patient per year by year 2 after diagnosis. The corresponding total CVD-related and HF-related costs were SEK 110,268 (€11,578) and SEK 84,956 (€8,920) per patient per year in the first year, which decreased to SEK 43,590 (€4,577) and 26,038 (€2,734) per patient per year by year 2, respectively. Therefore, HF-specific inpatient and outpatient costs accounted for 69% of the total costs in the first year after HF diagnosis, declining to 49% during the second year after diagnosis (and 46% during the third year after diagnosis). Inpatient care constituted more than 90% of the total secondary healthcare costs per patient at 1 year after diagnosis for all-cause (SEK 112,432 [€11,805]), CVD-related (SEK 106,101 [€11,141]), and HF-related (SEK 82,897 [€8,704]) events. By comparison, the mean total primary care cost for the Uppsala County cohort only was SEK 10,347 (€1,086) per patient per year (Table S4; Supplementary Information).

Drivers of high resource use and costs

Of the total patient population, 10% (n=465) incurred approximately 40% of the total secondary healthcare costs. Compared with 'other' (non-resource intensive) patients, those in

For peer review only

the resource-intensive population were likely to have a lower mean age, belong to the male sex, have a mean NT-proBNP level above 3,000 pg/mL, and a number of comorbidities; had a known LVEF; and had not received either an angiotensin-converting enzyme inhibitor or angiotensin receptor blocker (Table 3).

Discussion

This study found that (i) the absolute number of patients with HF in Sweden continues to increase; (ii) the total cost of secondary care of prevalent patients with HF increased and was primarily driven by costs associated with inpatient care; (iii) patients with newly diagnosed HF were at a high risk of frequent hospitalizations during the first year after diagnosis, especially in those diagnosed in secondary vs primary care; (iv) HFpEF was associated with greater resource use compared with HFrEF or an unknown LVEF; (v) during the first year, hospitalizations (inpatient care) constituted more than 90% of the total secondary healthcare costs, with 69% of all secondary healthcare costs being attributable to HF-specific care; and (iv) approximately 10% of the patients were highly resource-intensive.

In HF management, hospitalizations are a key challenge because they are highly resource intensive and costly.[3, 4, 15-17] Not only are patients with HF frequently hospitalized, but duration of their admissions are also often lengthy, particularly in Europe compared with the US.[(15, 16)] In the current study, the mean hospital LOS during the first year after HF diagnosis was more than 16 days for patients admitted for all-cause or CVD-related events and 12.8 days for patients admitted for HF-related events.

The clinical implication of our data is to identify factors responsible for the high costs of HF within the first year of diagnosis. Such factors could include patients being discharged from hospital too early to ensure full evaluation, patients not being initiated on evidence-based

For peer review only

therapy or devices, delayed or incomplete follow-up in patient management programmes, and loss of continuity and/or ineffective HF follow-ups.

An important observation concerned the risk of hospitalization/readmission over the follow-up period, which was significantly greater for patients receiving their first HF diagnosis in secondary vs primary care. This might be the result of patients presenting with more severe HF and/or more severe chronic HF and patients with a diagnosis of acute HF. Accordingly, the total costs associated with primary care, available for the Uppsala County only, were SEK 10,347 (€1,086) per patient and were much lower than the secondary care costs in the first year after diagnosis. These costs generally remained stable over the first 4 years of follow-up and increased slightly at year 5, which could be a result of the increasing age of the population.

Patients with HFpEF were also at a higher risk of all-cause and CVD-related hospitalizations and required more outpatient visits at 3 years than those with HFrEF, potentially because patients with HFpEF tended to be older and/or have a higher comorbidity burden. Importantly, a higher proportion of deaths in patients with HFpEF than in those with HFrEF may have a non-CVD cause.[18, 19] Mortality data presented in Lindmark et al[(20)] show that the HR for the 1-year all-cause mortality rate was significantly lower for HFrEF than for HFpEF (0.77, 95% CI 0.62–0.96, P=0.0159).

We found a decline in resource use observed after the first year of diagnosis and substantial reductions in healthcare costs, with total all-cause costs per patient decreasing by more than half in the second year. This decrease reflects patient-level, longitudinal data trends and, in fact, from a total population/healthcare system perspective, the opposite was true with an increase in costs over time because of an increasing prevalence of HF and a shift in patient profile towards older and sicker patients.[20] In our incident population, the high financial costs during the first year after diagnosis may be partly explained by survivor bias,

For peer review only

assuming that patients alive in later years have less severe HF symptoms and require less cost-intensive treatment, such as the need for re-hospitalizations and longer LOS. Other possible explanations include the use of both more intensive investigations to define the aetiology of HF during the first year and expensive medicines, devices, and/or surgical treatments during the initial phase of treatment. The costs reported herein are generally similar to those reported in another Swedish EMR- and register-based study that analyzed data from 2006.[4]

Similar to other economic studies in HF,[3, 21, 22] the inpatient care accounted for most of the total secondary care costs (~90%) and showed the greatest proportional decline in costs from the peak at 1 year after diagnosis, consistent with the reduction seen in the number of hospitalizations. Similar trends were observed for CVD-related and HF-related costs per patient. Data for the 10% of patients who accumulated the highest healthcare costs showed that patients with several different comorbidities were associated with a higher resource use. The most resource-intensive patients tended to be male with high NT-proBNP levels. Patients with high/increasing biomarker levels tend to be followed up closely by specialists because of the potential for adverse outcomes.[23]

A limitation of the current study was the challenge of retrospectively identifying patients with confirmed HF. The use of two, rather than one, HF diagnoses was expected to increase the certainty surrounding the HF diagnosis, although it could potentially have led to exclusion of more recent or mild cases of HF. Second limitation concerned the definition of subgroups based on the HF phenotype. The limited number of echocardiographs required for the verification of the HF diagnosis was a major drawback, meaning that a large proportion of patients had missing information for both LVEF and structural and functional pathologies. This may have introduced some level of bias because it could be assumed that the echocardiography was performed only in more severe cases of HF, meaning that the two HF

For peer review only

phenotype subgroups were not wholly representative of the total cohort. Nevertheless, our data reflect the real-world costs associated with a diagnosis of HF, irrespective of the true validation of the diagnosis. Moreover, the threshold for defining HFrEF was an LVEF of ≤50%, which is higher than the recommendations from the ESC of <40%[24]; therefore, a larger number of patients would have been categorized as having HFrEF. Finally, there were missing data on doctor visits for the Västerbotten County cohort because of technical issues relating to the Västerbotten EMR database; thus, primary care resource use and costs were available only for the Uppsala County cohort.

Conclusions

Care for patients with HF represents an increasing financial burden on the Swedish healthcare system. Hospitalizations/readmissions represent a key resource and cost burden regardless of HF phenotype, particularly during the first year after diagnosis, with total costs of HF attributed primarily to those associated with inpatient care. Furthermore, characteristics of patients incurring the greatest healthcare costs included younger age, male sex, and high CCI scores and NT-proBNP levels at the time of the HF diagnosis. Understanding the profile of patients with HF who are likely to incur the greatest healthcare costs is invaluable to guide resource allocation.

Acknowledgements

The authors thank Japinder Kaur of Novartis and Sharon Smalley and Carly Sellick of Pharma Genesis, London, for providing medical writing assistance with this manuscript.

Funding This research was funded by Novartis Pharma AG, Basel, Switzerland.

Competing interest K. Boman, M. Olofsson, and J. Stålhammar received reimbursement from Novartis via IQVIA for performing the study. K. Boman and M. Olofsson have also

For peer review only

received lecture grants from Novartis. K. Lindmark received lecture grants and consultant fees from Novartis. G. Wikström has no conflicts of interest to declare; however, Uppsala University received research funding from Novartis for conducting this study. M. Costa-Scharplatz is an employee of Novartis Sweden AB, Sweden, and A.F. Fonseca is an employee of Novartis Pharma AG, Basel, Switzerland. Data extraction and analysis were conducted by S. Johansson, V. Heller, and M. Törnblom are employees of IQVIA, Sweden, which was commissioned to conduct the study on behalf of Novartis Pharma AG, Basel, Switzerland, and have ongoing consulting and research relationships with Novartis Pharma AG.

Data availability statement Major findings from the study will be published in scientific manuscripts only. The data will not be made available in any other format in order to preserve the privacy of the patients in compliance with local laws and regulation.

Authors contributions

Kurt Boman and Gerhard Wikström were involved in the study and manuscript conceptualization, interpretation of results and development of the first draft of the manuscript. Krister Lindmark contributed to protocol development, study and manuscript conceptualization, results interpretation, refinement of analysis requirements and writing of the manuscript. Stina Johansson, Vincent Heller, Michael Törnblom performed statistical analyses. Jan Stålhammar and Mona Olofsson contributed to protocol development, study conceptualization, interpretation of results and supported the development of the manuscript. Madlaina Costa-Scharplatz and Ana Filipa Fonseca contributed to manuscript conceptualization, interpretation of results, refinement of analysis requirements and supported the development of the manuscript. All authors reviewed the manuscript and approved the final draft of the manuscript for submission.

For peer review only

Figures caption

Figure 1 Mean number of hospitalizations (lines in black) and outpatient visits (lines in grey) for incident patients with HF during 2010–2012 by year after HF diagnosis.

CVD, cardiovascular disease; HF, heart failure; HFpEF, HF with preserved ejection fraction; HFrEF, HF with reduced ejection fraction; LVEF, left ventricular ejection fraction.

Notes: Number of patients 1, 2, and 3 years after diagnosis: 4,648, 2,769, and 1,309 total patients; 1,106, 667, and 323 patients with HFrEF; 609, 353, and 156 patients with HFpEF; 2,933, 1,749, and 830 with Unknown LVEF, respectively.

Figure 2 All-cause, CVD-related, and HF-related total, inpatient, and outpatient costs up to 3 years after diagnosis of HF (incident patients with HF, 2010–2012).

CVD, cardiovascular disease; HF, heart failure; SEK, Swedish krona.

Notes: Number of patients: year 1, n=4,648; year 2, n=2,769; year 3, n=13,095. All costs are given in SEK 2015 values (1 SEK = €0.105).

For peer review only

References

1. Bui AL, Horwich TB, Fonarow GC. Epidemiology and risk profile of heart failure. *Nat Rev Cardiol*. 2011;**8**(1):30-41.

2. Socialstyrelsen (The National Board of Health and Welfare). Statistics on the causes of death 2015. https://www.socialstyrelsen.se/globalassets/sharepoint-dokument/artikelkatalog/statistik/2016-8-3.pdf 2016 [Accessed on 19 December 2017]

3. Stalhammar J, Stern L, Linder R, Sherman S, Parikh R, Ariely R, Deschaseaux C, Wikstrom G. The burden of preserved ejection fraction heart failure in a real-world Swedish patient population. *J Med Econ*. 2014;**17**(1):43-51.

4. Stalhammar J, Stern L, Linder R, Sherman S, Parikh R, Ariely R, Wikstrom G. Resource utilization and cost of heart failure associated with reduced ejection fraction in Swedish patients. *J Med Econ*. 2012;**15**(5):938-46.

5. Sveriges Kommuner och Landsting (SKL) SKoL. Socialstyrelsen. Öppna jämförelser 2014. Hälso-och sjukvård. Jämförelser mellan landsting. Del 2. Indikatorer om sjukdomar och behandlingar. **https://webbutik.skr.se/bilder/artiklar/pdf/7585-157-0.pdf?issuusl=ignore.** 2014 [Accessed on 2 February 2018].

6. Agvall B, Borgquist L, Foldevi M, Dahlstrom U. Cost of heart failure in Swedish primary healthcare. *Scand J Prim Health Care*. 2005;**23**(4):227-32.

7. Ryden-Bergsten T, Andersson F. The health care costs of heart failure in Sweden. *J Intern Med*. 1999;**246**(3):275-84.

8. Boman K LK, Stalhammar J, Olofsson M, Costa-Scharplatz M, Fonseca AF, Bruce Wirta S, Castelo-Branco A, Tornblom M, Wikstrom G. Total annual healthcare costs of heart

For peer review only

failure between 2005 and 2014: a retrospective, population-based study in Sweden. . Poster presented at the European Meeting of the *International Society for Pharmacoeconomics and Outcomes Research* (ISPOR) 2018;10–14 November 2018, Barcelona, Spain.

9. Heidenreich PA AN, Allen LA, Bluemke DA, Butler J, Fonarow GC, Ikonomidis JS, Khavjou O, Konstam MA, Maddox TM, Nichol G, Pham M, Pina IL, Trogdon JG, American Heart Association Advocacy Coordinating C, Council on Arteriosclerosis T, Vascular B, Council on Cardiovascular R, Intervention, Council on Clinical C, Council on E, Prevention, Stroke C. Forecasting the impact of heart failure in the United States: a policy statement from the American Heart Association. *Circ Heart Fail* 2013;**6**:606–19.

10. Martinell M, Jan Stålhammar, J., Hallqvist, J. Automated data extraction—A feasible way to construct patient registers of primary care utilization. *Upsala Journal of Medical Sciences*. 2012;**117**:52-6.

11. Statistics Sweden. Consumer price index [Internet]. **http://www.scb.se/en/finding-statistics/statistics-by-subject-area/prices-and-consumption/consumer-price-index/consumer-price-index-cpi/**. 2016 [Accessed on 12 January 2016].

12. National Board of Health and Welfare. 2015 Vägledning till NordDRG. Swedish version. **https://www.socialstyrelsen.se/globalassets/sharepoint-dokument/artikelkatalog/ovrigt/2015-12-29.pdf**

. 2016 [Accessed 2 February 2018].

13. Tandvårds- och läkemedelsförmånsverket TTSDaPBA. **https://www.tlv.se/in-english/prices-in-our-database.html.** [Accessed 5 March 2018].

14. McMurray JJ, Adamopoulos S, Anker SD, Auricchio A, Bohm M, Dickstein K, Falk V, Filippatos G, Fonseca C, Gomez-Sanchez MA, Jaarsma T, Køber L, Lip GYH, Maggioni AP, Parkhomenko A, Pieske BM, Popescu BA, Rønnevik PK, Rutten FH, Schwitter J, Seferovic P, Stepinska J, Trindade PT, Voors AA, Zannad F, Zeiher A, Bax JJ, Baumgartner

For peer review only

H, Ceconi C, Dean V, Deaton C, Fagard R, Funck-Brentano C, Hasdai D, Hoes A, Kirchhof P, Knuuti J, Kolh P, McDonagh T, Moulin C, Popescu BA, Reiner Z, Sechtem U, Sirnes PA, Tendera M, Torbicki A, Vahanian A, Windecker A, McDonagh T, Sechtem U, Bonet LA, Avraamides P, Ben Lamin HA, Brignole M, Coca A, Cowburn P, Dargie H, Elliott P, Flachskampf FA, Guida GF, Hardman S, Lung B, Merkely B, Mueller C, Nanas JN, Nielsen OW, Ørn S, John T. Parissis JT, Ponikowski P. ESC Guidelines for the diagnosis and treatment of acute and chronic heart failure 2012: The Task Force for the Diagnosis and Treatment of Acute and Chronic Heart Failure 2012 of the European Society of Cardiology. Developed in collaboration with the Heart Failure Association (HFA) of the ESC. *Eur Heart J*. 2012;**33**(14):1787-847.

15. Cowie MR, Anker SD, Cleland JGF, Felker GM, Filippatos G, Jaarsma T, Jourdain P, Knight E, Massie B, Ponikowski P, López-Sendón J. Improving care for patients with acute heart failure: before, during and after hospitalization. *ESC Heart Fail*. 2014;**1**(2):110-45.

16. Braunschweig F, Cowie MR, Auricchio A. What are the costs of heart failure? *Europace*. 2011;**13** Suppl 2:ii13-7.

17. Kilgore M, Patel HK, Kielhorn A, Maya JF, Sharma P. Economic burden of hospitalizations of Medicare beneficiaries with heart failure. *Risk Manag Healthc Policy*. 2017;**10**:63-70.

18. Dunlay SM, Redfield MM, Weston SA, Therneau TM, Hall Long K, Shah ND, Roger VL. Hospitalizations after heart failure diagnosis a community perspective. *J Am Coll Cardiol*. 2009;**54**(18):1695-702.

19. Chan MM, Lam CS. How do patients with heart failure with preserved ejection fraction die? *Eur J Heart Fail*. 2013;**15**(6):604-13.

20. Lindmark K, Boman K, Olofsson M, Tornblom M, Levine A, Castelo-Branco A, Schlienger R, Wirta SB, Stålhammar J, Wikström G. Epidemiology of heart failure and

For peer review only

trends in diagnostic work-up: a retrospective, population-based cohort study in Sweden. *Clin Epidemiol*. 2019;**11**:231-44.

21. Stafylas P, Farmakis D, Kourlaba G, Giamouzis G, Tsarouhas K, Maniadakis N, Parissis J. The heart failure pandemic: The clinical and economic burden in Greece. *Int J Cardiol*. 2017;**227**:923-9.

22. Mejhert M, Lindgren P, Schill O, Edner M, Persson H, Kahan T. Long term health care consumption and cost expenditure in systolic heart failure. *Eur J Intern Med*. 2013;**24**(3):260-5.

23. Kim HN, Januzzi JL, Jr. Natriuretic peptide testing in heart failure. *Circulation*. 2011;**123**(18):2015-9.

24. Ponikowski P, Voors AA, Anker SD, Bueno H, Cleland JGF, Coats AJS, Falk V, González-Juanatey JR, Harjola V, Jankowska EA, Jessup M, Linde C, Nihoyannopoulos P, Parissis JT, Pieske B, Riley JP, Rosano GMC, Ruilope LM, Ruschitzka F, Rutten FH, Meer P. 2016 ESC Guidelines for the diagnosis and treatment of acute and chronic heart failure: The Task Force for the diagnosis and treatment of acute and chronic heart failure of the European Society of Cardiology (ESC)Developed with the special contribution of the Heart Failure Association (HFA) of the ESC. *Eur Heart J*. 2016;**37**(27):2129-200.

For peer review only

Tables

Table 1 Demographic and clinical characteristics at baseline for incident patients with HF from the Uppsala and Västerbotten counties with a first HF diagnosis in 2010–2012, overall and by HF phenotype

Characteristic	Overall population (N=5,205)	HF phenotype<br>HFpEF (n=652)	HFrEF (n=1,167)	Unknown LVEF (n=3,386)
Mean age at index date (SD), years	76.8 (12.3)	74.8 (12.2)	70.3 (13.0)	79.4 (11.1)
Median age at index date (IQR), years	79.0 (69.5, 85.7)	76.8 (68.3, 83.6)	71.2 (63.1, 80.3)	81.5 (73.4, 87.2)
Sex, n (%)				
Women	2,405 (46.2)	341 (52.3)	380 (32.6)	1,684 (49.7)
Men	2,800 (53.8)	311 (47.7)	787 (67.4)	1,702 (50.3)
Mean NT-proBNP level (SD), pg/mL	4,909.1 (8,202.9)	4,270.8 (7,576.7)	6,217.1 (9,495.2)	4,543.4 (7,730.5)
Mean SBP/DBP (SD), mmHg	139.7 (25.6)/ 78.7 (15.3)	141.8 (27.1)/ 76.8 (14.6)	134.9 (24.3)/ 79.7 (15.3)	140.9 (25.6)/ 78.8 (15.3)
Mean eGFR (SD), mL/min/1.73 m^2	51.9 (29.1)	49.6 (27.9)	60.6 (30.2)	44.2 (26.0)
Mean CCI[a,b] (SD)	1.8 (2.2)	1.8 (2.2)	1.7 (2.1)	1.9 (2.2)
Common comorbidities and risk factors, n (%)[b,c]				
Hypertension	2,679 (51.5)	366 (56.1)	476 (40.8)	1,837 (54.3)
Atrial fibrillation	1,593 (30.6)	212 (32.5)	247 (21.2)	1,134 (33.5)
IHD (angina or MI)	1,198 (23.0)	122 (18.7)	303 (26.0)	773 (22.8)
Diabetes	952 (18.3)	137 (21.0)	197 (16.9)	618 (18.3)
Cancer	739 (14.2)	118 (18.1)	141 (12.1)	480 (14.2)
Dyslipidaemia	676 (13.0)	89 (13.7)	152 (13.0)	435 (12.8)
Cerebrovascular disease	660 (12.7)	70 (10.7)	109 (9.3)	481 (14.2)
Anaemia	645 (12.4)	104 (16.0)	101 (8.7)	440 (13.0)

CCI, Charlson comorbidity index; DBP, diastolic blood pressure; eGFR, estimated glomerular filtration rate; HF, heart failure; HFpEF, HF with preserved ejection fraction; HFrEF, HF with reduced ejection fraction; IHD, ischaemic heart disease; IQR, interquartile range; LVEF, left ventricular ejection fraction; MI, myocardial infarction; NT-pro-BNP, N-terminal pro-B-type natriuretic peptide; SBP, systolic blood pressure; SD, standard deviation.

For peer review only

Notes: Data are either number (n) and percentage (%) or mean (SD). [a]Includes patients with a CCI of zero (ie no comorbidities). [b]Comorbidities and underlying cardiac diseases occurring 0–5 years before the index date. [c]Comorbidities and underlying cardiac diseases occurring in ≥10% of the overall population.

For peer review only

Table 2 Sub-distribution hazard model for the proportion of incident patients with HF from the Uppsala and Västerbotten counties with a second HF diagnosis in 2010–2012 who had CVD-related hospitalizations within 3 years and cumulative incidence function estimates for the proportion of patients hospitalized within 0.5, 1, and 3 years after index date

**Characteristic**	**N=4,648**	**Cumulative incidence function of CVD-related hospitalizations, % (95% CI)**			**Sub-distribution HR (95% CI)**	***P* value**
		**0.5 years**	**1 year**	**3 years**		
Age group, years						
18–54 (Reference)	275	46.9 (40.9–52.7)	52.0 (45.9–57.7)	62.5 (55.9–68.3)	1	
55–64	457	47.0 (42.4–51.5)	56.5 (51.8–60.9)	71.8 (66.7–76.3)	1.15 (0.95–1.41)	0.16
65–74	1,051	47.3 (44.2–50.3)	57.5 (54.4–60.4)	72.5 (69.2–75.4)	1.21 (1.01–1.45)	0.0383
75–84	1,592	44.6 (42.1–47.0)	55.3 (52.9–57.7)	75.6 (72.8–78.0)	1.21 (1.02–1.45)	0.0305
≥85	1,273	41.9 (39.2–44.6)	52.2 (49.4–54.9)	70.7 (67.8–73.4)	1.13 (0.94–1.35)	0.19
Sex						
Female (Reference)	2,143	43.8 (41.7–45.9)	52.9 (50.8–55.0)	71.7 (69.4–73.9)	1	
Male	2,505	45.8 (43.8–47.7)	56.5 (54.6–58.4)	73.0 (70.9–74.9)	1.06 (0.98–1.14)	0.12
Diagnosis setting						
Primary care (Reference)	1,118	41.8 (38.9–44.6)	53.3 (50.3–56.2)	77.1 (73.8–80.0)	1	
Secondary care	3,528	45.8 (44.2–47.4)	55.3 (53.7–57.0)	71.0 (69.3–72.7)	0.94 (0.86–1.02)	0.12
Unknown	2	N/A	N/A	N/A	1.99 (1.32–3.00)	0.0011
HF phenotype						
HFrEF (Reference)	609	53.2 (49.2–57.1)	61.1 (57.1–64.8)	77.9 (73.7–81.5)	1	
HFpEF	1,106	52.4 (49.5–55.3)	60.9 (58.0–63.7)	73.8 (70.8–76.6)	0.93 (0.83–1.06)	0.28
Unknown LVEF	2,933	40.3 (38.5–42.0)	51.3 (49.5–53.1)	70.9 (68.9–72.8)	0.79 (0.71–0.88)	<0.0001
NT-proBNP level, pg/mL						
0–1,000 (reference)	912	40.4 (37.2–43.5)	51.8 (48.5–54.9)	72.4 (68.8–75.7)	1	
1,001–3,000	1,283	45.4 (42.7–48.1)	56.6 (53.8–59.2)	76.1 (73.1–78.7)	1.10 (0.99–1.21)	0.07
>3,000	1,601	52.5 (50.0–54.9)	61.3 (58.8–63.6)	74.3 (71.7–76.7)	1.19 (1.08–1.32)	0.0006
Missing	852	34.5 (31.3–37.7)	43.5 (40.2–46.8)	63.2 (59.4–66.9)	0.83 (0.74–0.93)	0.0017

CI, confidence interval; CVD, cardiovascular disease; HF, heart failure; HFpEF, HF with preserved ejection fraction; HFrEF, HF with reduced ejection fraction; HR, hazard ratio; LVEF, left ventricular ejection fraction; NT-proBNP, N-terminal pro-B-type natriuretic peptide.

For peer review only

Note: The Fine and Grays sub-distribution hazard model was used to obtain the sub-distribution HRs. Cumulative incidence function was used to estimate percentage of patients with CVD-related hospitalizations within 0.5, 1, and 3 years after index date.

For peer review only

Table 3 Logistic regression to determine characteristics at HF diagnosis of the 10% of incident patients with HF incurring the greatest secondary healthcare costs during the first year after index date

		Unadjusted model		Adjusted model	
**Characteristics at HF diagnosis**	**N=5,205 n (%)**	**OR**	**95% CI**	**OR**	**95% CI**
Age at index date, years					
18–54	297 (5.7%)	Reference		Reference	
55–64	511(9.8%)	0.88	0.60–1.29	0.83	0.56–1.24
65–74	1,155 (22.2%)	0.89	0.64–1.24	0.82	0.57–1.18
75–84	1,796 (34.5%)	0.40	0.28–0.57	0.39	0.27–0.57
≥85	1,446 (27.8%)	0.18	0.12–0.27	0.21	0.13–0.33
Sex					
Male	2,800 (53.8%)	Reference		Reference	
Female	2,405 (46.2%)	0.73	0.61–0.88	0.98	0.80–1.20
LVEF					
HFrEF	1,167 (22.4%)	Reference		Reference	
HFpEF	652 (12.5%)	1.01	0.79–1.30	1.05	0.80–1.39
Unknown	3,386 (65.1%)	0.29	0.24–0.36	0.41	0.33–0.52
NT-proBNP, ng/L					
0–1,000	1,075 (20.7%)	Reference		Reference	
1,001–3,000	1,433 (27.5%)	1.17	0.88–1.56	1.48	1.09–2.01
>3,001	1,730 (33.2%)	1.77	1.36–2.30	1.93	1.45–2.57
Missing	967 (18.6%)	1.17	0.85–1.60	1.44	1.03–2.02
ACEI or ARB treatment					
ACEI	2,481 (47.7%)	Reference		Reference	
ACEI and ARB	58 (1.1%)	1.81	0.88–3.74	1.61	0.75–3.45
ARB	964 (18.5%)	1.02	0.79–1.31	1.01	0.77–1.33
Neither ACEI nor ARB	1,702 (32.7%)	1.26	1.03–1.55	1.63	1.31–2.03
Hypertension					
No	2,526 (48.5%)	Reference		Reference	
Yes	2,679 (51.5%)	1.11	0.93–1.33	1.06	0.84–1.33
Atrial fibrillation					
No	3,612 (69.4%)	Reference		Reference	
Yes	1,593 (30.61)	0.75	0.61–0.92	0.77	0.61–0.97
Angina or MI					
No	4,007 (76.9%)	Reference		Reference	
Yes	1,198 (23.02%)	1.45	1.19–1.78	1.27	1.00–1.60

For peer review only - http://bmjopen.bmj.com/site/about/guidelines.xhtml

Diabetes					
No	4,253 (81.71%)	Reference		Reference	
Yes	952 (18.29%)	1.48	1.19–1.83	1.19	0.93–1.53
Cancer					
No	4,466 (85.80%)	Reference		Reference	
Yes	739 (14.20%)	1.19	0.93–1.52	1.17	0.89–1.53
Cerebrovascular disease					
No	4,545 (87.32%)	Reference		Reference	
Yes	660 (12.68%)	1.08	0.83–1.41	1.27	0.95–1.71
Dyslipidaemia					
No	4,529 (87.01%)	Reference		Reference	
Yes	676 (12.99%)	1.60	1.26–2.03	1.10	0.83–1.47
Anaemia					
No	4,560 (87.61%)	Reference		Reference	
Yes	645 (12.39%)	1.73	1.36–2.20	1.63	1.24–2.14
COPD					
No	4,801 (92.24%)	Reference		Reference	
Yes	404 (7.76%)	1.27	0.93–1.74	1.13	0.80–1.58
Aortic insufficiency/regurgitation					
No	4,814 (92.49%)	Reference		Reference	
Yes	391 (7.51%)	3.21	2.49–4.13	2.54	1.92–3.37
Dementia					
No	4,883 (93.81%)	Reference		Reference	
Yes	322 (6.19%)	0.33	0.19–0.60	0.53	0.29–0.99
Chronic kidney disease					
No	4,987 (95.81%)	Reference		Reference	
Yes	218 (4.19%)	2.47	1.76–3.48	1.44	0.97–2.14
Depression					
No	5,015 (96.35%)	Reference		Reference	
Yes	190 (3.65%)	1.38	0.90–2.13	1.21	0.75–1.93
Mitral insufficiency/regurgitation					
No	5,030 (96.64%)	Reference		Reference	
Yes	175 (3.36%)	3.32	2.34–4.72	2.58	1.74–3.81
Peripheral artery disease					
No	5,086 (97.71%)	Reference		Reference	
Yes	119 (2.29%)	2.47	1.57–3.87	1.60	0.97–2.65

ACEI, angiotensin-converting enzyme inhibitor; ARB, angiotensin receptor blocker; CI, confidence interval; COPD, chronic obstructive pulmonary disease; HF, heart failure; HFpEF, heart failure with preserved ejection fraction; HFrEF, heart failure with reduced ejection fraction; LVEF, left ventricular ejection fraction; MI, myocardial infarction; NT-proBNP, N-terminal pro-B-type natriuretic peptide; OR, odds ratio.

For peer review only - http://bmjopen.bmj.com/site/about/guidelines.xhtml

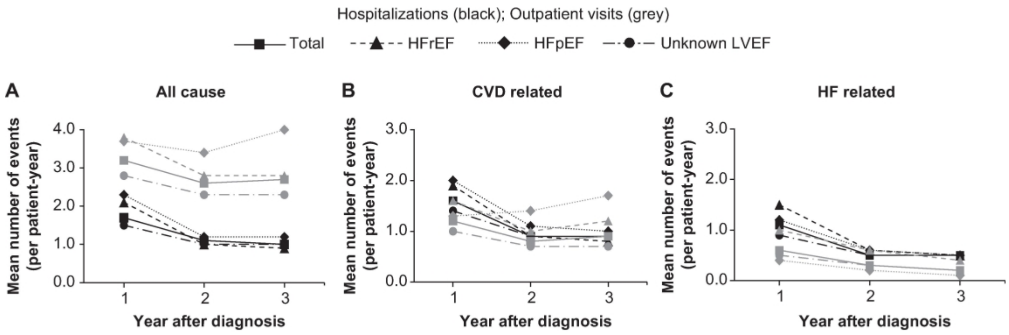

Figure 1 Mean number of hospitalizations (lines in black) and outpatient visits (lines in grey) for incident patients with HF during 2010–2012 by year after HF diagnosis.
CVD, cardiovascular disease; HF, heart failure; HFpEF, HF with preserved ejection fraction; HFrEF, HF with reduced ejection fraction; LVEF, left ventricular ejection fraction.
Notes: Number of patients 1, 2, and 3 years after diagnosis: 4,648, 2,769, and 1,309 total patients; 1,106, 667, and 323 patients with HFrEF; 609, 353, and 156 patients with HFpEF; 2,933, 1,749, and 830 with Unknown LVEF, respectively.

177x57mm (600 x 600 DPI)

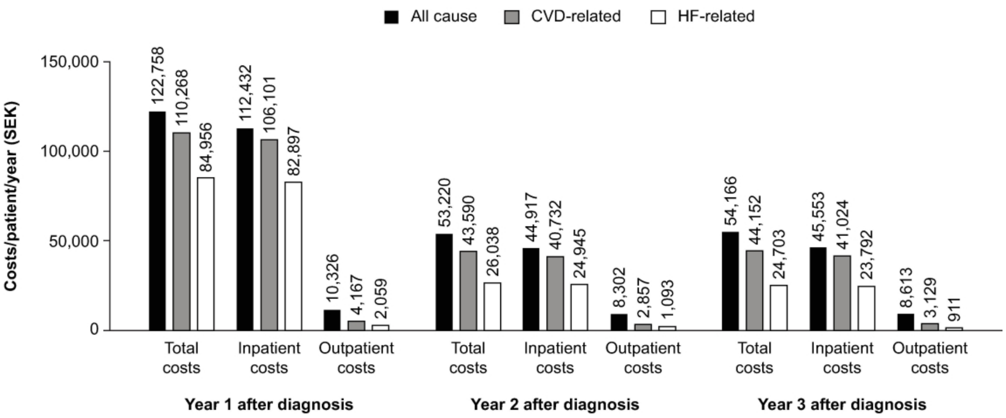

Figure 2 All-cause, CVD-related, and HF-related total, inpatient, and outpatient costs up to 3 years after diagnosis of HF (incident patients with HF, 2010–2012).
CVD, cardiovascular disease; HF, heart failure; SEK, Swedish krona.
Notes: Number of patients: year 1, n=4,648; year 2, n=2,769; year 3, n=13,095. All costs are given in SEK 2015 values (1 SEK = €0.105).

172x70mm (600 x 600 DPI)

Supporting information

Secondary healthcare costs: pharmacotherapy (the Uppsala County cohort)

In the cohort of patients from the Uppsala County with incident patients with heart failure (HF) during the period of 2010–2012 (N=3,452), the overall drug use (HF-related drugs and other pharmacotherapies) in the year after diagnosis was associated with a mean cost of Swedish krona (SEK) 10,727 (equivalent to €1,126) per patient per year. In general, these costs remained stable for up to 4 years after the diagnosis of HF. However, at year 5, an increase was observed in the mean cost to SEK 12,760 (€1,340) per patient per year. The costs of HF-specific therapy (drugs listed in Table S2) constituted 13% of the total drug costs in the first year after HF diagnosis (mean costs per patient per year at years 1 and 5 after HF diagnosis were SEK 1,395 [€146] and 1,404 [€147], respectively).

For peer review only - http://bmjopen.bmj.com/site/about/guidelines.xhtml

Table S1 List of ICD-10 codes

**Comorbidity/risk factor**	**ICD-10 code**
Hypertension	I10–I13, I15
Atrial fibrillation	I48
Angina or MI	I20–I22
Diabetes	E10–E14
Cancer	C00–C97
Cerebrovascular disease	I60–I69
Dyslipidaemia	E78
Anaemia	D50–D64
COPD	J40–J44, J47
Aortic insufficiency/regurgitation	I06, I35, Q231
Dementia	F00–F07
Chronic kidney disease	N18
Depression	F32–F33
Mitral insufficiency/regurgitation	I051, I340, Q233
Peripheral artery disease	I739
**Definitions for calculation of CCI**	
MI	I21–I22, I252
Congestive heart failure	I43, I50, I099, I110, I130, I132, I255, I420, I425–I429, P290
Peripheral vascular disease	I70–I71, I731, I738–I739, I771, I790, I792, K551, K558–K559, Z958–Z959

For peer review only - http://bmjopen.bmj.com/site/about/guidelines.xhtml

Cerebrovascular disease	G45–G46, I60–I69, H340
Dementia	F00–F03, G30, F051, G311
Chronic pulmonary disease	J40–J47, J60–J67, I278–I279, J684, J701, J703
Connective tissue disease-rheumatic disease	M05–M06, M315, M32–M34, M351, M353, M360
Peptic ulcer disease	K25–K28
Mild liver disease	B18, K73–K74, K700–K703, K709, K713–K715, K717, K760, K762–K764, K768–K769, Z944
Diabetes without complications	E100–E101, E106, E108–E111, E116, E118–E121, E126, E128–E131, E136, E138–E141, E146, E148–E149
Diabetes with complications	E102–E105, E107, E112–E115, E117, E122–E125, E127, E132–E135, E137, E142–E145, E147
Paraplegia and hemiplegia	G81–G82, G041, G114, G801–G802, G830–G834, G839
Renal disease	N18–N19, N052–N057, N250, I120, I131, N032–N037, Z490–Z492, Z940, Z992
Cancer	C00–C76, C81–C97
Moderate to severe liver disease	K704, K711, K721, K729, K765–K767, I850, I859, I864, I982
Metastatic carcinoma	C77–C80
AIDS/HIV	B20–B22, B24

AIDS/HIV, human immunodeficiency virus/acquired immunodeficiency syndrome; CCI, Charlson comorbidity index; COPD, chronic obstructive pulmonary disease; ICD-10, International Classification of Diseases and Related Health Problems, Tenth Revision; MI, myocardial infarction.

For peer review only - http://bmjopen.bmj.com/site/about/guidelines.xhtml

Table S2 Heart failure-related drugs (according to the European Society of Cardiology 2012 guidelines[9])

**Drug/drug class**	**Anatomical Therapeutic Chemical code**
Angiotensin-converting enzyme inhibitor	C09A, C09B
Angiotensin receptor blocker	C09C, C09D
Beta blocker	C07
Mineralocorticoid receptor antagonist	C03DA01-C03DA04
Ivabradine (sinus node I_f channel inhibitor)	C01EB17
Diuretics	C03
Digoxin	C01AA05
Nitrates and nitrites	C01DA

For peer review only - http://bmjopen.bmj.com/site/about/guidelines.xhtml

Table S3 Sub-distribution hazard model of the proportion of incident patients with HF from the Uppsala and Västerbotten counties with a second HF diagnosis in 2010–2012 who had all-cause hospitalizations within 3 years and cumulative incidence function estimates for the proportion of hospitalized patients within 0.5, 1, and 3 years after index date

**Characteristic**	**N=4,648**	**Cumulative incidence function of all-cause hospitalizations, % (95% CI)**			**Sub-distribution HR (95% CI)**	***P* value**
		**0.5 years**	**1 year**	**3 years**		
Age group, years						
18–54 (reference)	275	51.6 (45.6–57.4)	58.2 (52.1–63.8)	69.5 (63.0–75.0)	1	
55–64	457	50.3 (45.6–54.8)	60.4 (55.7–64.7)	75.2 (70.1–79.5)	1.07 (0.88–1.29)	0.50
65–74	1,051	50.5 (47.5–53.5)	60.7 (57.7–63.6)	75.8 (72.7–78.6)	1.11 (0.93–1.31)	0.24
75–84	1,592	46.2 (43.8–48.7)	57.4 (54.9–59.8)	77.3 (74.7–79.8)	1.06 (0.90–1.25)	0.51
≥85	1,273	43.4 (40.6–46.1)	53.7 (51.0–56.4)	72.4 (69.5–75.0)	0.97 (0.82–1.16)	0.77
Sex						
Female (reference)	2,143	46.3 (44.2–48.4)	55.6 (53.5–57.7)	74.5 (72.2–76.6)	1	
Male	2,505	47.8 (45.9–49.8)	59.1 (57.1–61.0)	75.5 (73.4–77.4)	1.04 (0.97–1.12)	0.28

For peer review only - http://bmjopen.bmj.com/site/about/guidelines.xhtml

Diagnosis setting						
Primary care (reference)	1,118	43.7 (40.8–46.6)	56.1 (53.1–58.9)	79.9 (76.7–82.8)	1	
Secondary care	3,528	48.2 (46.5–49.8)	57.9 (56.3–59.5)	73.6 (71.8–75.2)	0.93 (0.86–1.01)	0.07
Unknown	2	N/A	N/A	N/A	1.80 (1.15–2.82)	0.0098
HF phenotype						
HFrEF (reference)	609	56.5 (52.4–60.3)	64.7 (60.8–68.4)	80.5 (76.5–83.9)	1	
HFpEF	1,106	54.9 (51.9–57.8)	63.7 (60.8–66.5)	76.4 (73.4–79.0)	0.91 (0.81–1.03)	0.14
Unknown LVEF	2,933	42.3 (40.5–44.1)	53.6 (51.8–55.4)	73.6 (71.6–75.4)	0.77 (0.70–0.86)	<0.0001
NT-proBNP, pg/mL						
0–1,000 (reference)	912	43.0 (39.7–46.2)	55.2 (51.9–58.3)	76.4 (73.0–79.6)	1	
1,001–3,000	1,283	47.3 (44.6–50.0)	58.6 (55.9–61.3)	78.0 (75.2–80.6)	1.06 (0.97–1.17)	0.21
>3,000	1,601	54.5 (52.0–56.9)	63.7 (61.3–66.0)	76.4 (73.9–78.7)	1.19 (1.07–1.31)	0.0007
Missing	852	37.6 (34.3–40.8)	46.6 (43.2–49.9)	66.2 (62.5–69.7)	0.84 (0.75–0.94)	0.0028

For peer review only - http://bmjopen.bmj.com/site/about/guidelines.xhtml

CI, confidence interval; HF, heart failure; HFpEF, HF with preserved ejection fraction; HFrEF, HF with reduced ejection fraction; HR, hazard ratio; LVEF, left ventricular ejection fraction; NT-proBNP, N-terminal pro-B-type natriuretic peptide.

Note: Multivariate sub-distribution hazard model with death as competing risk used to obtain the sub-distribution HRs. Cumulative incidence function was used to estimate percentage of patients with all-cause hospitalizations in 0.5, 1, and 3 years after index date.

For peer review only

For peer review only - http://bmjopen.bmj.com/site/about/guidelines.xhtml

Table S4 Costs associated with primary care contacts for incident patients with HF between 2010 and 2012 in the Uppsala County

	**Mean (SD) costs per patient per year in SEK**				
	**Year 1 (N=3,453)**	**Year 2 (N=2,705)**	**Year 3 (N=1,846)**	**Year 4 (N=891)**	**Year 5 (N=166)**
Total costs	10,347 (12,685)	9,521 (11,974)	9,490 (12,225)	9,754 (12,755)	10,869 (16,857)
Family physician visits	5,432 (8,634)	4,718 (7,723)	4,668 (7,652)	4,641 (7,693)	5,153 (7,714)
Nurse visits	4,050 (7,251)	4,068 (7,475)	4,100 (7,546)	4,388 (8,107)	4,992 (11,685)
Blood tests	866 (1,254)	736 (1,143)	722 (1,133)	726 (1,128)	724 (1,034)

HF, heart failure; SD, standard deviation; SEK, Swedish krona.

Notes: Primary care costs (available only for the Uppsala County cohort) per visit or per test were calculated based on published price lists. All costs are in SEK 2015 values (1 SEK = €0.105).

For peer review only - http://bmjopen.bmj.com/site/about/guidelines.xhtml

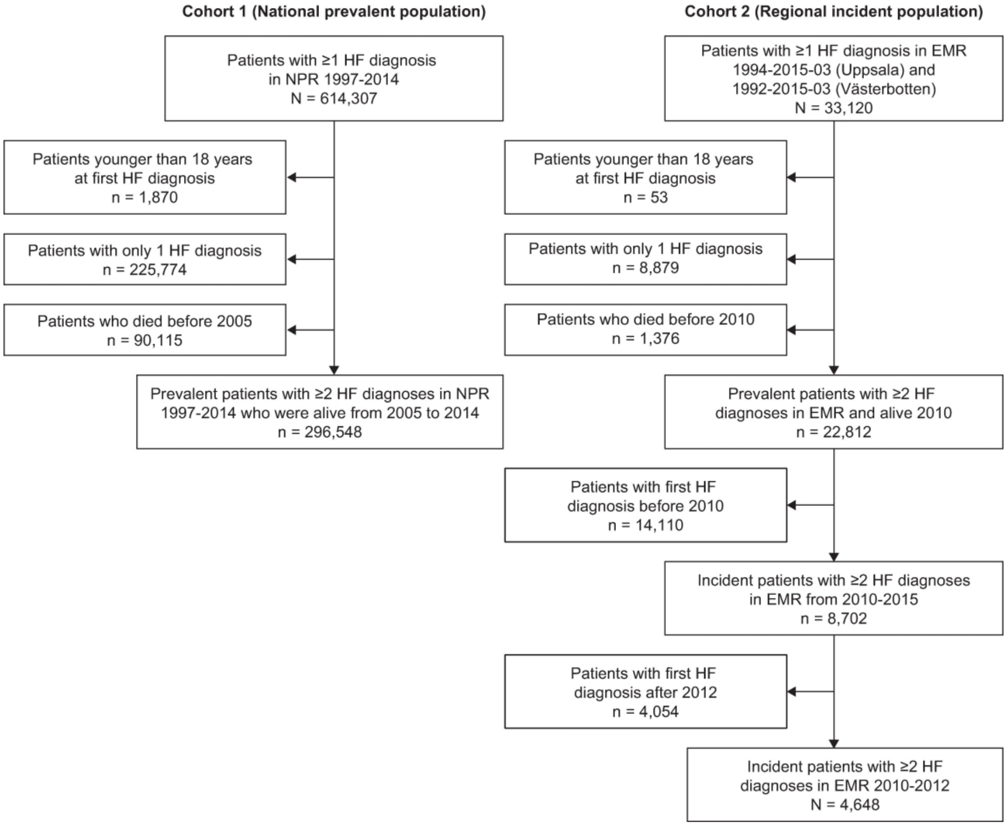

Figure S1: Patient flow. EMR, electronic medical record; HF, heart failure; HFpEF, HF with preserved ejection fraction; HFrEF, HF with reduced ejection fraction; LVEF, left ventricular ejection fraction; NPR, National Patient Register. %"Notes: In cohort 1, data from 614,307 patients were extracted from the Swedish NPR on the basis of an observed HF diagnosis between 1997 and 2014; of which, data from 296,548 patients met the inclusion criteria of at least two HF diagnoses in the analysis period (2005–2014) with no HF diagnosis in the look-back period (1997–2004). In cohort 2, data from 33,120 patients were extracted from the EMRs on the basis of an observed HF diagnosis between 1994 and 2015 (Uppsala County) and between 1992 and 2015 (Västerbotten County); of which, data from 4,648 patients met the inclusion criteria of at least two HF diagnoses in the analysis period (2010–2012) and no HF diagnosis in the look-back period (NPR: 1997–2009, EMR: 1992–2009). A total of 4,648 analyzable patients with 3-year follow-up data were identified between 2010 and 2012. Of the 1,715 patients for whom LVEF data were available, 64,5% had HFrEF and 35,5% had HFpEF. The incident HF population comprised those with no HF diagnosis in the look-back periods.

177x145mm (600 x 600 DPI)

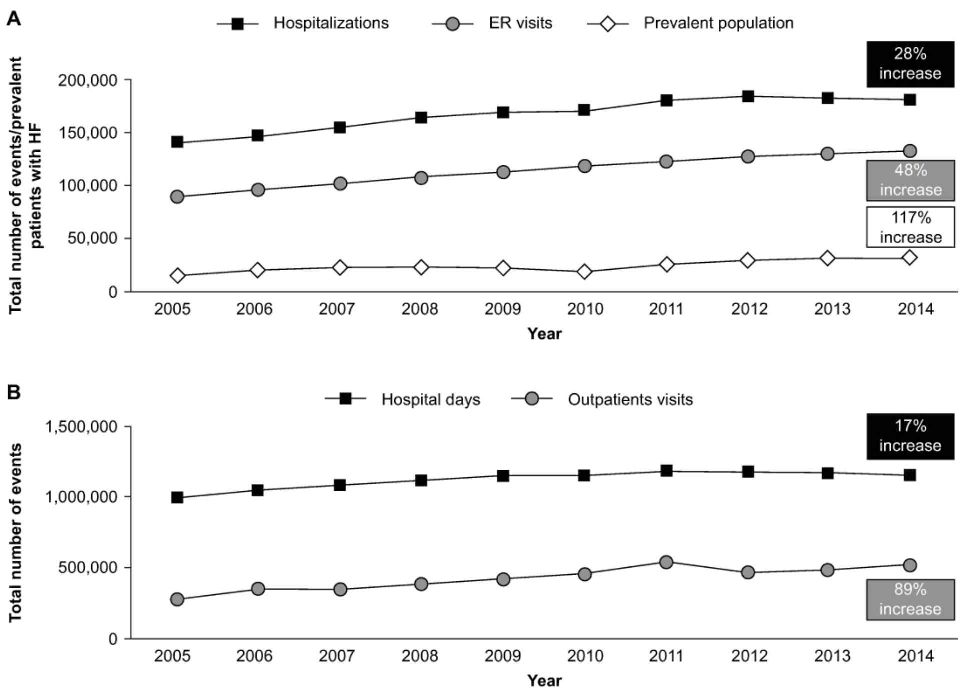

	2005	2006	2007	2008	2009	2010	2011	2012	2013	2014
Persons >18 years old in Sweden, n	7,113,513	7,179,337	7,251,275	7,331,508	7,419,589	7,496,476	7,563,649	7,627,772	7,692,386	7,762,073
Age-adjusted prevalence of HF in Sweden, % (CI)	1.30 (1.31–1.29)	1.37 (1.38–1.36)	1.44 (1.45–1.43)	1.51 (1.52–1.50)	1.56 (1.57–1.55)	1.61 (1.62–1.60)	1.66 (1.67–1.65)	1.69 (1.70–1.69)	1.71 (1.72–1.70)	1.72 (1.72–1.71)

Figure S2: Total number of (a) hospitalizations and ER visits and (b) hospital days and outpatient visits in relation to the absolute number of prevalent adult patients with HF extracted from the NPR, size of adult population in Sweden, and age-adjusted prevalence of HF in Sweden from 2005 to 2014. CI, confidence interval; ER, emergency room; HF, heart failure; NPR, National Patient Register.

171x134mm (600 x 600 DPI)

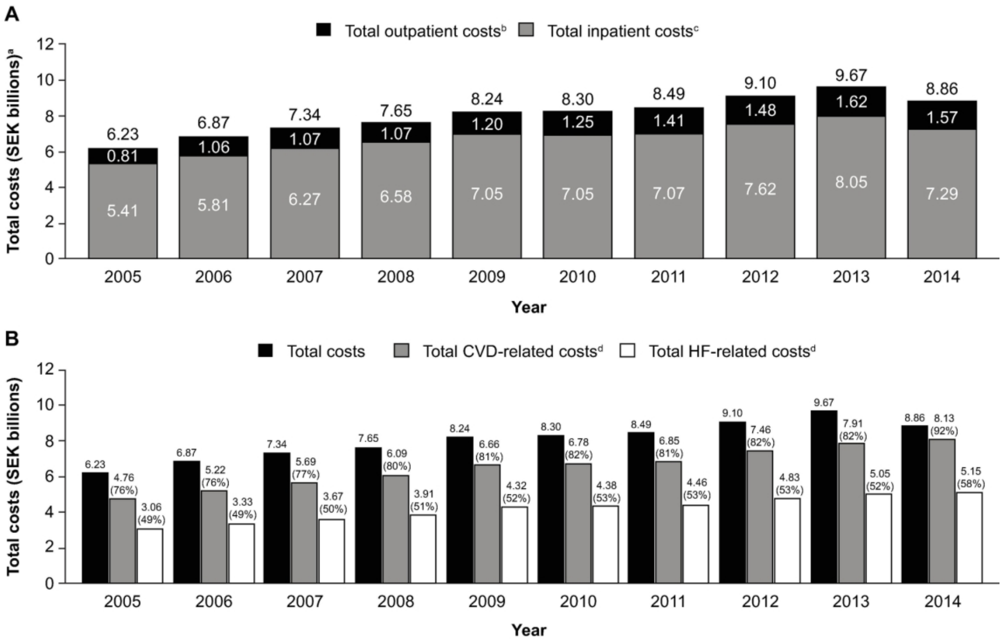

Figure S3 Costs associated with secondary care of prevalent patients with HF from 2005 to 2014: (a) total, inpatient, and outpatient costs and (b) total, CVD-related, and HF-related costs.
CVD, cardiovascular disease; ER, emergency room; HF, heart failure; SEK, Swedish krona.
Notes: aNumbers above bars indicate total costs (sum of inpatient and outpatient costs). bTotal inpatient costs correspond to the costs associated with hospitalizations. cTotal outpatient costs are the sum of the costs associated with outpatient and ER visits. dCVD-related and HF-related costs are not mutually exclusive; percentages shown in brackets represent the proportion of CVD-related and HF-related costs from the total costs.

169x108mm (300 x 300 DPI)

STROBE 2007 (v4) Statement—Checklist of items that should be included in reports of *cohort studies*

Section/Topic	Item #	Recommendation	Reported on page #
**Title and abstract**	1	(*a*) Indicate the study's design with a commonly used term in the title or the abstract	1
		(*b*) Provide in the abstract an informative and balanced summary of what was done and what was found	2
**Introduction**			
Background/rationale	2	Explain the scientific background and rationale for the investigation being reported	4
Objectives	3	State specific objectives, including any prespecified hypotheses	4
**Methods**			
Study design	4	Present key elements of study design early in the paper	4-5
Setting	5	Describe the setting, locations, and relevant dates, including periods of recruitment, exposure, follow-up, and data collection	4-5
Participants	6	(*a*) Give the eligibility criteria, and the sources and methods of selection of participants. Describe methods of follow-up	5
		(*b*) For matched studies, give matching criteria and number of exposed and unexposed	NA
Variables	7	Clearly define all outcomes, exposures, predictors, potential confounders, and effect modifiers. Give diagnostic criteria, if applicable	7-8
Data sources/ measurement	8*	For each variable of interest, give sources of data and details of methods of assessment (measurement). Describe comparability of assessment methods if there is more than one group	7-8
Bias	9	Describe any efforts to address potential sources of bias	6
Study size	10	Explain how the study size was arrived at	NA
Quantitative variables	11	Explain how quantitative variables were handled in the analyses. If applicable, describe which groupings were chosen and why	7-8
Statistical methods	12	(*a*) Describe all statistical methods, including those used to control for confounding	8
		(*b*) Describe any methods used to examine subgroups and interactions	7-8
		(*c*) Explain how missing data were addressed	NA
		(*d*) If applicable, explain how loss to follow-up was addressed	NA
		(*e*) Describe any sensitivity analyses	NA
**Results**			

For peer review only

Participants	13*	(a) Report numbers of individuals at each stage of study—eg numbers potentially eligible, examined for eligibility, confirmed eligible, included in the study, completing follow-up, and analysed	8-9
		(b) Give reasons for non-participation at each stage	NA
		(c) Consider use of a flow diagram	9 (Figure S1)
Descriptive data	14*	(a) Give characteristics of study participants (eg demographic, clinical, social) and information on exposures and potential confounders	9
		(b) Indicate number of participants with missing data for each variable of interest	NA
		(c) Summarise follow-up time (eg, average and total amount)	6
Outcome data	15*	Report numbers of outcome events or summary measures over time	8-12
Main results	16	(*a*) Give unadjusted estimates and, if applicable, confounder-adjusted estimates and their precision (eg, 95% confidence interval). Make clear which confounders were adjusted for and why they were included	8
		(*b*) Report category boundaries when continuous variables were categorized	NA
		(*c*) If relevant, consider translating estimates of relative risk into absolute risk for a meaningful time period	NA
Other analyses	17	Report other analyses done—eg analyses of subgroups and interactions, and sensitivity analyses	9
**Discussion**			
Key results	18	Summarise key results with reference to study objectives	12-15
**Limitations**			
Interpretation	20	Give a cautious overall interpretation of results considering objectives, limitations, multiplicity of analyses, results from similar studies, and other relevant evidence	14-15
Generalisability	21	Discuss the generalisability (external validity) of the study results	12-15
**Other information**			
Funding	22	Give the source of funding and the role of the funders for the present study and, if applicable, for the original study on which the present article is based	15

*Give information separately for cases and controls in case-control studies and, if applicable, for exposed and unexposed groups in cohort and cross-sectional studies.

Note: An Explanation and Elaboration article discusses each checklist item and gives methodological background and published examples of transparent reporting. The STROBE checklist is best used in conjunction with this article (freely available on the Web sites of PLoS Medicine at http://www.plosmedicine.org/, Annals of Internal Medicine at http://www.annals.org/, and Epidemiology at http://www.epidem.com/). Information on the STROBE Initiative is available at www.strobe-statement.org.

For peer review only
